# Supplementary material for: Single-cell transcriptome profiling of an adult human cell atlas of 15 major organs
Source: Genome Biol. 2020 Dec 7;21:294. doi: 10.1186/s13059-020-02210-0 (PMC7720616; doi:10.1186/s13059-020-02210-0)
Supplement: Supplementary file 12 — Additional file 12. Review history. [file 13059_2020_2210_MOESM12_ESM.docx]

Review History

**First round of review**

**Reviewer 1**

**Are you able to assess all statistics in the manuscript, including the appropriateness of statistical tests used?** Yes, and I have assessed the statistics in my report.

**Comments to author:**

In the manuscript, He et al performed scRNA-seq of 15 tissue of one healthy adult donor and generated an adult human cell atlas. The authors identified 234 subtypes of cells and performed trajectory and interaction analysis. While of potential interest, for example, identification of novel cell types in skin, the present manuscript suffers so many flaws. It is a negligent and sloppy work.

Major problems:

1)	The authors are short of basic immunological issues. I. gdT cells originate from DN T (CD4-CD8-) cells, instead of naïve T cells. So, gdT should be removed form trajectory results. II. They claimed that 'In addition, the clusters with different states showed an organ specific pattern', they should illustrate which clusters derive from marrow, and which from spleen? III. There were so many TCR/BCR contigs without 'cdr3' in Table S4/S5. I think those 'productive' contigs with 'cdr3' may be identical TCR/BCR sequences. The results of TCR/BCR and Table S4/S5 are problematic. IV. As shown in Figure 3G, many B clusters had no expansion and transition capacity, where those B cells come from? V. As shown in phylogenetic tree in Figure 4A, the differences between DC and Mac is smaller than differences among MAC, is it possible? VI. As shown in Figure 4F, the DC (8) developed to Mac (15), impossible!!! VI. The Langerhans cells are a subset of DC, instead of Mac.

2)	The authors claimed that 'The hierarchical clustering analysis revealed that all classical monocytes were closely related as a branch node, while the intermediate and non-classical monocytes were closely related to macrophages'. That's wrong!!! In this figure, the intermediate (cluster 10 and 5) and non-classical monocytes (cluster 3) were closely related to classical monocytes (cluster 6, 1, 11, and 9), it was obvious.

3)	What were the 'Mixed cells'? were they doublets (Figure S2)? The authors evaluate the doublets just using marker genes of different cell types, that is not enough. Some software in GitHub (such as, DoubletFinder, DoubletDetection, DoubletDecon, and scds) may be hulpful.

4)	The results of cell clustering are too rough. I. There was no CD4, CD8, B, and myocardial cells in heart, no any progenitor cells in marrow (Figure S2)? II. The authors should annotate each cluster in Figure S3B. III. What the different colours means in Figure 1C? IV. Cell cluster annotations were confused in Figure 1C, why several 'Epithelial cell' existed? V. In figure S5, I do not know what is 'B Cells 1', 'B Cells 2', 'B Cells 3', what is the difference among them? How they distribute in various tissues? The same to other cells (such as cells in Figure 5C). VI. There was no any Tfh or Th in Figure 2A? VII. There was no any mast cells in a healthy donor and no DC in the lymph node (Figure S2)?

5)	Authors claim to identify rare and novel cell populations (Langerhans cells, COCH+ fibroblast, and SCGB2A2+ epithelial cells), however, they did not show their tissue distribution. Did those three clusters exist in Figure S3B? And Langerhans cell is not a novel cell type. Where is the Langerhans cell in Figure 4A?

6)	Figure legends were careless and ambiguous. There was no annotation for colour in each heatmap and trajectory results, the simple annotations should exist in figures.

7)	The authors claimed that 'various TFs regulate the development of B cells to plasma cells.' That is lack of evidence, they should provide trajectory analyses just like CD4/CD8 T cells.

8)	The origin of Mac is ambiguous, the authors should claim which Mac come from monocytes and which from proliferation cells.

9)	The authors should provide a volcano figure for DEGs between two groups (13 digestion-related clusters vs. 20 non-digestion-related clusters) before Figure 5D.

10)	What are the 'FibSmo' cells? The authors should describe what they are, and are they doublets or novel type cells? If they are a new type of cells, the authors should confirm their existent with experiment.

11)	Problems in data analysis. I. The gene expression matrix was obtained with CellRanger (version 3.0.1), while TCR/BCR contigs were generated with CellRanger (version 3.1.0). Why the authors chose different version? II. The authors should use the 'NormalizeData' function before the 'ScaleData' function. III. When the authors identified contaminated genes, they just randomly sampled one time. They should be cautious to randomly sample and set the cutoff, because that 10X-dataset has high dropout ratio, especial for the genes with low expression. IV. The authors used the MHC II genes to measure the 'Presenting-antigen ability', how about the MHC I genes?

Minor problems:

1)	This manuscript is suspected of rubbing hot spots, what's the relationship of this manuscript and the COVID-19? There was no alveolar cells in their dataset.

2)	Are their countries different in address 1 and address 3? (P. R. China VS China)

3)	A figure should be used to show the sequencing saturation, instead of a table (Additional file 20: Table S2).

4)	Why the 'Number of Read Pairs' in Table S2 among tissue is so different (nearly 4-fold higher)?

5)	Authors claim to identify 91,393 cells after QC filter, what's the number of total cell before QC?

6)	The figure S1 should be separated into two parts to show 'UMI' and 'gene' respectively.

7)	What was the expression cutoff for 'genes with high expression', and how many genes used in Figure S4? I think correlation value should range from -1 to 1, instead of -4 to 4, and authors should provide the statistical P value for the correlation in Figure S4.

8)	The authors should provide cell number of each cluster in each tissue in Figure S6A.

9) The authors claimed that 'we preformed TCR clonal typing accompanied with transcriptome analysis (Fig. 1E).' where is the Figure 1E? they also claimed that 'we observed consistent results of TCR diversity using β chains (Additional file 8: Figure S7J-I).' I think it was Figure S7J-L.

10)	What did each row present in Figure S7H? what did the size mean in Fig. 2F and Fig. 3F?

11)	There was no legend for Figure 3B in the figure. (Just like Figure 5B)

12)	What is the cutoff for DEGs among B cells? There were so many gene with P > 0.05 in Table S5.

13)	There were many TFs in monocyte-macrophage development in this manuscript, such as NFIL3, RXRA, ELF2, NR1H3, SPIC and HDAC2. The expression of those genes should be presented with figures.

14)	It is an arbitrary decision to name the cell clusters, the column names of Figure S14 are different from cluster names (such as Figure S11D).

15)	In Figure 6C, each figure presents a tissue?

16)	In Figure S15, S16, and S17, the '-pvalue' is 1.5? maybe '-logP'.

17)	It is better to add interactions among various tissues.

**Reviewer 2**

**Are you able to assess all statistics in the manuscript, including the appropriateness of statistical tests used?** There are no statistics in the manuscript.

**Comments to author:**

GBIO-D-20-00356

Summary:

He et al have compiled a single-cell RNA-seq and VDJ-seq dataset including 15 different organs from a single individual. The authors describe cell types and patterns of B and T cell clone distributions across organs. While many of the findings are expected, the authors make some interesting observations, however, the lack of replicates and experimental validation weakens any conclusion regarding novel and/or rare cell types.

Major comments:

The provided figures were in illegible quality.

This study has profiled only one donor. Given the high biological variation in humans, the applicability of these findings to the broader population are under question.

Authors overinterpret the detection of higher nUMIs in skin and trachea samples as having "higher transcriptional activation". This is impossible to know without having technical replicates and unlikely to be the case for all cell types in one organ.

The identification of rare/novel cell types (e.g. Langerhan cells and COCH+ fibroblast subtypes) should be supported by replicates/validation.

The authors should provide a complete description of the tissue dissociation method. In addition, they should comment on how the use of different dissociation protocols could result in selective release of cells in different organs and/or induction of stress response.

Authors should provide the cell type labels instead of cluster numbers in Figure 2 D and throughout the manuscript.

The accuracy of cell type annotations throughout the manuscript is unclear. The expression of marker genes should be shown in a heatmap or dot plot in the main figure.

Automated doublet detection should be compared to manual exclusion of doublets.

What do authors refer to as "unassigned" cell types (e.g. "Clusters due to cell doublets or unassigned cell types were identified and excluded manually <..>")?

The pseudotime analysis of T cells and myeloid cells reflects transcriptional similarities. The authors overinterprete the connection of these trajectories to the developmental stage of cells, provide no new insights or lack of experimental validation or further supporting data. The analysis of gene expression dynamics along pseudotime could add value to this analysis. The exclusion of cell types from a different lineage could improve the trajectory analysis, for example, gamma delta T cells.

The transcription factor signature in T cells that comprised FOS, FOSB, JUN, JUND, etc is concerning since these have been previously shown to be upregulated in response to tissue dissociation-induced stress (Brink et al, 2017 Nature Methods). The authors should explain how they can exclude this is not an artifact of the tissue processing.

The claims related to COVID-19 are unjustified given the lack of biological replicates and the absence of data from key organs involved in the disease (upper and lower airways). Recently, researchers have used single-cell datasets to address this question in a thorough manner (Sungnak et al 2020, https://arxiv.org/abs/2003.06122; Ziegler et al., in revision, https://papers.ssrn.com/sol3/papers.cfm?abstract_id=3555145). "Our dataset revealed ACE2 expression in multiple cell types and organs, which overlapped with the suffered organs (lung, heart, trachea, stomach, small intestine, artery and muscle) of HCoV-19 infection[75]."

Authors use pseudotime trajectory inference on circulating monocytes and macrophages pooled from all tissues, however, they fail to take into account that most tissue-resident macrophages are not derived from circulating monocytes but from embryonic progenitors (e.g. self-renewing embryonic macrophages in the skin, liver or gut). As mentioned above, the consideration of the biological context is essential for a robust interpretation of trajectory analysis.

Epithelial cell analysis is focused on biology that has been previously described. The authors could use this dataset to further understand the core-similarities between epithelial cell subsets (e.g. secretory cells) across tissues as well as the key differences.

The purpose of the CellphoneDB interaction analysis is unclear. The authors should show ligand-receptors pairs in the context of the specific cell types expressing them. Furthermore, they should indicate how they calculated the frequency of any given interaction and should not interpret the meaning of this metric.

**Authors Response**

**Point-by-point responses to the reviewers’ comments:**

**Reviewer #1:**

In the manuscript, He et al performed scRNA-seq of 15 tissue of one healthy adult donor and generated an adult human cell atlas. The authors identified 234 subtypes of cells and performed trajectory and interaction analysis. While of potential interest, for example, identification of novel cell types in skin, the present manuscript suffers so many flaws. It is a negligent and sloppy work.

**Response:** We are very appreciative of the positive comments on our manuscript as well criticisms on some analyses and presentations, which are very helpful to improve our manuscript. In this revision, we have performed additional analyses and experimental validation, and presented our results with our best efforts, according to the Reviewers’ comments and suggestions.

**Major problems:**

1) The authors are short of basic immunological issues.

I. gdT cells originate from DN T (CD4-CD8-) cells, instead of naïve T cells. So, gdT should be removed form trajectory results.

**Response:** Thanks for the suggestions. Accordingly, we performed a new trajectory analysis by removing the γδ T cells from the CD8^+^ T cells in this revision. We observed consistent trend of development status in the trajectory tree, where T_N_ cells sprout into T_h1_, T_CM_, and T_RM_ branches for CD4^+^ T cells and T_EFF_, T_CM_, IEL, and T_RM_ branches for CD8^+^ T cells **(Fig. 2C**). We have updated the result in the main text. Please kindly check the **RESULTS** section in Line 2-4, Paragraph 3, Page 7.

II. They claimed that 'In addition, the clusters with different states showed an organ specific pattern', they should illustrate which clusters derive from marrow, and which from spleen?

**Response:** Thanks for the question. Previously, we observed that T_RM_ cells exhibited different states across different tissues. We had tried to track the origins of T cells in different states using TCR clonotypes. For CD4^+^ T cells, only two LINC00861_T_N_ T cells in the marrow and the blood shared common TCR clonotypes with two TNF_T_RM_ cells in the esophagus and the lymph node. For CD8^+^ T cells, only one KLF2_T_N_ T cell from the spleen shared a common TCR with five MITX_IEL cells in the rectum. As such, we had not included these observations based on few cells in our previous submission. Further study with sufficient number of T_RM_ cells would be more insightful to understanding their origins from marrow or spleen. In this revision, we acknowledged that “the T_RM_ clusters with different states exhibited an organ-specific pattern (**Additional file 10: Figure S7E, F**), while their origin from marrow or spleen is unclear due to the limited number of cells”. Please kindly check the **RESULTS** section in second last sentences, Paragraph 3, Page 7.

III. There were so many TCR/BCR contigs without 'cdr3' in Table S4/S5. I think those 'productive' contigs with 'cdr3' may be identical TCR/BCR sequences. The results of TCR/BCR and Table S4/S5 are problematic.

**Response:** Thanks for the thoughtful comments. We defined TCR and BCR clonotypes with criteria including 1) at least one paired α/β CDR3 chain for T cells and at least one paired H/L chain for B cells and 2) only clonotypes with high confidence based on “cellranger vdj". A productive VDJ contig was only considered if it met the requirements listed in the documentation of algorithm from 10X Genomics via [https://support.10xgenomics.com/single-cell-vdj/software/pipelines/latest/algorithms/ annotation](https://support.10xgenomics.com/single-cell-vdj/software/pipelines/latest/algorithms/%20annotation).

Bearing the Reviewer’s comment in mind, we revisited our analytic scripts for filtering and realized an inappropriate setting, which resulted in a few TCRs with only single α or β chain and BCRs with only L or H chain, as well as the exclusion of TCRs with high quality in the previous manuscript (previous **Tables S4-S5**). In this revision, we fixed the scripts and updated the two tables (**Additional file 9:** **Tables S26-S27** for TCR and **Additional file 12: Table S32** for BCR). Moreover, because the numbers of TCR and BCR were increased, we also updated the results in relation to TCR and BCR. Please kindly check the content in the **RESULTS** section in Paragraph 3, Page 8 and Paragraph 2, Page 11. We also updated the figures and tables, including **Fig. 2F-I, Figure S8A-L**, and **Table S26-27** for T cells (**Fig. 2F-I**, **Figure S7A-L** and **Table S4** in the previous submission) and **Fig. 3G, F, Figure S9G-K**, and **Table S32** for B cells (**Fig. 3G, F, Figure S8E-I** and **Table S5** in the previous submission).

Given that multiple productive TCR α and β chains (Schuldt *et al., J Immunol* 2019, 202:637-644) and more than one antibodies (Shi *at el., Cell Discov* 2019, 5:64) have been demonstrated in T and B cells, respectively, we note that the productive contigs with CDR3 are not identical TCR/BCR sequences. In addition, the scripts for the filtering were provided in GitHub together with all other scripts implemented in this manuscript.

IV. As shown in Figure 3G, many B clusters had no expansion and transition capacity, where those B cells come from?

**Response:** Thanks for the question. In our study, we observed various numbers of B cells with detectable BCRs or clonotypes among different organ tissues, ranging from 40 to 3,497 (below **Response Table 1**). Moreover, the proportions of clonal cells in the cells with clonotypes are variously below 9%, which is consistent with previous reports that the proportion of the clonal B cell varied across healthy individuals in the gut, liver, and peripheral blood (0.1%-18%; Chung *et al., Hepatol Commun* 2018, 2:956-967; Bashford-Rogers *et al., Genome Res* 2013, 23:1874-1884), suggesting the diversity and richness of B cell repertoire in human organs. Similarly, the numbers of clonal B cells were also various among different B cell clusters, and scarcely found in some clusters, such as TCL1A_ly_naive_B, TCL1A_naive_B and TNF_memory_B cell clusters (below **Response Table 2**). Therefore, considering the richness of diverse B cell repertoire but the limited number of clonal B cells detected, we could not observe obvious expansion and transition capacity of certain B cell subtypes, as shown in **Fig. 3G**, **Figure S9J-L** in this revision (**Fig. 3G** and **Figure S8H-I** in the previous submission, respectively).

**Response Table 1. Clonal B cells among different organ tissues.**

| Tissue | Cells with detectable clone types | Clonal cells / all cells with detectable clone types (%) |
| --- | --- | --- |
| Lymph node | 3497 | 0.06 |
| Spleen | 972 | 3.4 |
| Stomach | 814 | 8.48 |
| Rectum | 784 | 3.96 |
| Marrow | 441 | 0.68 |
| Blood | 208 | 0.96 |
| Trachea | 122 | 2.46 |
| Esophagus | 104 | 4.81 |
| Small intestine | 85 | 0 |
| Liver | 57 | 0 |
| Bladder | 40 | 0 |

**Response Table 2. Clonal B cells among different cell clusters.**

| Cluster | Cells with detectable clone types | | Clonal cells / all cells with detectable clone types (%) |
| --- | --- | --- | --- |
| TCL1A_ly_naive_B | | 1760 | 0 |
| HSPA8_memory_B | | 1132 | 0.18 |
| TCL1A_naive_B | | 676 | 0 |
| LTB_memory_B | | 612 | 0.33 |
| IGKV3-20_plasma | | 578 | 14.19 |
| IGLV1-40_plasma | | 521 | 18.81 |
| TNF_memory_B | | 403 | 0 |
| CRIP1_memory_B | | 321 | 0 |
| GADD45B_memory_B | | 316 | 0 |
| IGHG1_plasma | | 259 | 19.7 |
| HIST1H4C_plasma | | 239 | 30.13 |
| IGKV1-39_plasma | | 152 | 26.32 |
| FABP5_memory_B | | 75 | 0 |
| IGKV3D-15_plasma | | 60 | 25 |
| MT2A_memory_B | | 20 | 0 |

**Response Figure 1**. **Heatmap showed the pairwise transition-index scores for B cells clusters.** Transition-index scores were indicated as colors from blue to red for low to high scores.

With the limited BCR data, we traced the links among different B cell clusters (above **Response Figure 1**). We observed that most of memory B cells and plasma cells shared BCR, suggesting that those B cells with identical BCRs were from the same origin. CRIP1_memory_B cell cluster also shared a certain amount of BCRs with TCL1A_ly_naive_B, suggesting their origin from naive B cell (TCL1A_ly_naive_B). These observations are consistent with the previous findings that B cells developed in the adult bone marrow (BM) from hematopoietic precursor cells to immature B cells, followed by B-cell activation taking place in GCs in spleen and lymph nodes, which leads to the development of plasma and memory B cells (Kathrin Pieper *et al., Allergy Clin Immunol.* 2013, 131(4):959-71).

In this revision, we added the above information in the main text, as read “BCR comparisons revealed that CRIP1_memory_B cell cluster shared a certain amount of BCRs with TCL1A_ly_naive_B, suggesting their origin from naïve B cell; and most of memory B cells and plasma cells shared identical BCR, suggesting that they might have a same origin (**Additional file 12: Table S32**). Unlike T cells, clonal diversity was common for B cells among all organs, while clonal expansion of B cells was limited and restricted in the spleen, rectum, and stomach (**Additional file 13: Figure S9J**). Moreover, less sharing of BCR clonotypes between B and plasma cells was observed across organs compared to T cells (**Fig. 2F and Fig. 3F**). In addition, BCR analysis showed lower expansion and transition abilities of B cells compared to plasma cells and T cells (**Fig. 2H, Fig. 3G, and Additional file 13: Figure S9K, L**), which might be due to the richness of diverse B cell repertoire but the limited number of clonal B cells detected.” Please kindly check these in the **RESULTS** section in Line 7-17, Paragraph 2, Page 11.

V. As shown in phylogenetic tree in Figure 4A, the differences between DC and Mac is smaller than differences among MAC, is it possible?

**Response:** Thanks for the question. The phylogenetic tree was constructed based on the gene profiles of different cell clusters (**Fig. 4C** in this revision). As pointed out by the Reviewer, the CD1C_DC (cluster 8) was grouped closely to APOE_Mac (cluster 15), which was then clustered with most of the other macrophage clusters. Their close clustering reflects their similar expression profiles, suggesting that these two groups of cells have some common features. Below t-SNE plot showed heterogeneous subpopulations in CD1C_DC (**Response Figure 2**). It might be possible that a certain proportion of these cells are monocyte-derived. Considering that monocyte-derived cells exist in steady-state tissues (Collin *et al.,* *Immunology*. 2018, 154(1):3-20) and share similar expression profile with macrophages under the quiescent status in the donor without pathogen stimulation (Chaussabel *et al.,* *Blood*. 2003, 102(2):672-81; Hashimoto *et al., Immunity*. 2011, 35(3):323-35). Consistently, we performed phylogenetic analysis using the transcription data reported previously (Tang-Huau *et al., Nat Commun* 2018, 9:2570), and observed that a subset of DC is grouped closely to macrophage clusters, suggesting their similar transcriptional profiles (below **Response Figure 3)**. Finally, we note that the CD1C_DC cluster contains only 350 cells from all 15 organs, and thus the limited number of cells with heterogeneous transcriptional background might introduce uncertainty to the clustering in the phylogenetic analysis.

**Response Figure 2. t-SNE plots of CD1C_DC cells.** Subpopulations (0-4) were indicated as dots in different colors. Each dot represents one cell.

**Response Figure 3**. **Phylogenetic analysis of transcriptomes of monocytes (Mon), macrophages (Mac), and dendritic cells (DC).** Data was derived from Tang-Huau. *et al.*

VI. As shown in Figure 4F, the DC (8) developed to Mac (15), impossible!!!

**Response:** We are very grateful for pointing out this mistake. In this revision, we have removed the DC (clusters 8 and 16) from the trajectory analysis, which revealed that the classical monocytes had initial state at the root, sprouting into branches with more developed states monocytes and then macrophages either directly or via the two intermediate monocytes (**Fig. 4E**; **Fig. 4F** in previous submission). We note that the updated result is consistent with our previous observation regarding the development of macrophages from monocytes. We also revised the related figures in the supplementary (**Figure S10B, C** in the **Additional file 14**). Please kindly check in Line 5-7, Paragraph 2, Page 12, in the **RESULTS** section, **Fig. 4E**, and **Figure S10B, C** in the **Additional file 14** in this revision.

VII. The Langerhans cells are a subset of DC, instead of Mac.

**Response:** Thanks for the comment. We initially defined skin Langerhans cells as a type of macrophages following a previous report (Davies *et al.，Nat Immunol* 2013, 14:986-995). In light of the Reviewer’s comment and accumulating evidence from later literatures, we now define the Langerhans cells as a subset of DC, namely CD207_DC in this revision. We have updated the name in relevant text and figures.

2) The authors claimed that 'The hierarchical clustering analysis revealed that all classical monocytes were closely related as a branch node, while the intermediate and non-classical monocytes were closely related to macrophages'. That's wrong!!! In this figure, the intermediate (cluster 10 and 5) and non-classical monocytes (cluster 3) were closely related to classical monocytes (cluster 6, 1, 11, and 9), it was obvious.

**Response:** Thanks for sharing the thoughtful viewpoint at different angle. Yes, we agreed with the Reviewer that overall the intermediate (clusters 10 and 5) and non-classical monocytes (cluster 3) were closely related to classical monocytes (clusters 6, 1, 11, and 9). Previously, we focused on the close relations between intermediate monocytes (clusters 10 and 5) and a subset of macrophages (cluster 13) as well as non-classical monocytes (cluster 3) and another subset of macrophages (cluster 2). In this revision, we described our observations as read “the hierarchical clustering analysis revealed that all classical monocytes were closely related as a branch node, as were non-classical monocyte and intermediate monocytes. Moreover, all macrophages were grouped together and separated from monocytes, except that two subsets of macrophages (clusters 2 and 13) were closely related to intermediate and non-classical monocytes (**Fig. 4C**).” Please check these in the **RESULTS** section in Line 4-8, Paragraph 1, Page 12.

3) What were the 'Mixed cells'? were they doublets (Figure S2)? The authors evaluate the doublets just using marker genes of different cell types, that is not enough. Some software in GitHub (such as, DoubletFinder, DoubletDetection, DoubletDecon, and scds) may be helpful.

**Response:** Thanks for the comments. Yes, the “Mixed cells” was referred to doublets. We agreed with the Reviewer that DoubletFinder would be a useful software to detect doublets in single cell sequencing dataset. Initially, we had applied DoubletFinder to our data. We observed that only a few cells were inferred as doublets and some proportion of cells were still self-clustered and deviated from other cells, meaning that these are likely doublets overlooked by the automated software. Therefore, we applied a more stringent method, which was based on the similarity of the markers and gene expression profiles of cell types to determine doublets manually. Below we used two tissues (muscle and heart) to compare the results from the manual method and the DoubletFinder.

Based on the expression of marker genes for cell types and the clustering of expression profiles, 15.76% and 10.8% of cells were identified as doublets manually in muscle and heart samples, respectively (**Response Figure 4A-C and Figure 5A-C**). These doublets could be clustered in a few clusters and had overlapping gene signatures of specific cell types (**Response Figure 4A and 5A**, red boxes). Moreover, each of the doublet-clusters highly expressed marker genes of various cell types (**Response Figure 4B and 5B**, red boxes). By contrast, 5% of cells were inferred as doublets for both samples using DoubletFinder (**Response Figure 4D and Figure 5D**), which is consistent with the documentation from 10X Genomics that more than 94% cells would be recovered as single cells when 14,000 cells were loaded to the channel. Specifically, 1,123 and 356 doublets were identified in the muscle tissue by the manual method and DoubletFinder, respectively; and that were 936 and 459 for the heart tissue. Moreover, the doublets identified manually cover 47.06% (216 out of 459) and 65.73% (234 out of 356) doublets identified by DoubletFinder in the muscle and heart tissues, respectively.

In order to minimize the potential impact of these doublets on the downstream analyses, we have applied the manual method as a more conservative way to remove doublets in our dataset. We have provided the comparison of automated and manual methods to remove doublets in the **Supplementary Methods** (**Additional file 30**).

**Response Figure 4. Doublets identification in muscle tissue.**

**A.** Heatmap of scaled expression for the top 10 differentially expressed genes of each cluster as numbered on top (based on log fold change) in the muscle. The clusters highlighted with red box showed co-expression of signatures of multiple cell types, which were indicated with red arrows. Doublet clusters were listed on the right.

**B.** Violin plot showed the normalized expression of marker gene of immune cell (*PTPRC*), endothelial cell (*PECAM1*), fibroblast (*MMP2*), smooth muscle cell (*ACTA2*), and satellite cell (*PAX7*) in different cell clusters labeled at the bottom. The highlighted clusters with red box indicated doublet cell clusters with co-expression of multiple cell type markers.

**C**. t-SNE plot showed the doublet clusters manually identified based on A and B. The doublets (mixed cells) were highlighted with black circles.

**D.** t-SNE plot showed the doublets identified using DoubletFinder, colored according to different confidence of doublets (red present a high confidence and blue are low). Singlet was marked in blue. The table on the right listed the numbers of singlets, doublets with a high and low confidence.

**Response Figure 5. Doublets identification in heart tissue.**

**A.** Heatmap of scaled expression for the top 10 differentially expressed genes of each cluster as numbered on top (based on log fold change) in the heart. The clusters highlighted with red box showed co-expression of signatures of multiple cell types, which were indicated with red arrows. Doublet clusters were listed on the right.

**B.** Violin plot showed the normalized expression of marker gene of T/NK (*CD3E*), endothelial cell (*PECAM1*), fibroblast (*MMP2*), and smooth muscle cell (*ACTA2*) in the different cell clusters labeled at the bottom. The highlighted clusters with red box indicated doublet cell clusters with co-expression of multiple cell type markers.

**C**. t-SNE plot showed the doublet clusters manually identified based on A and B. The doublets (mixed cells) were highlighted with black circles.

**D.** t-SNE plot showed the doublets identified using DoubletFinder, colored according to different confidence of doublets (red present a high confidence and blue are low). Singlet was marked in blue. The table on the right listed the numbers of singlets, doublets with a high and low confidence.

4) The results of cell clustering are too rough.

I. There was no CD4, CD8, B, and myocardial cells in heart, no any progenitor cells in marrow (Figure S2)?

**Response:** Thanks for the question. We defined a total of 276 cells as an NK/T cluster in the heart samples, which contain T cells and NK cells. However, subsets of CD4^+^, CD8^+^ T and NK cells were not shown separately in the t-SNE plot due to their small numbers. Below violin plot of gene expression for each cell clusters in heart showed the presence of CD4^+^ and CD8^+^ T cells in the NK/T cluster (**Response Figure 6**). We note that the expression of CD4^+^ marker is underrepresented as known in 10X Genomic platform, and thus the proportion of CD4^+^ cells should be much higher than what is shown in the violin plot. Only a few scattered B cells were identified in heart sample, which might be due to the low B cell content in the sampling anatomic position of heart and/or low representation of immune cells in our data. A previous study demonstrated only 3.1 ± 0.4% T cells (Pinto *et al., Circ Res* 2016, 118:400-409) and about 8% of B cells (about 8%) among CD45^+^ lymphocytes in the heart of human adult. In addition, a recent study didn’t capture any B cells from heart samples in two donors using single-cell sequencing (Han *et al., Nature* 2020, 581:303-309).

We were not able to obtain myocardial cells in heart likely because of two reasons as following. First, the availability and dissociation of viable myocyte cells from fresh tissue, particularly human, continues to hinder single-cell sequencing studies in the cardiac research area. Moreover, adult cardiomyocytes are too large to fit into most commercial single-cell microfluidic chambers, and do not generally well tolerate fluorescence activated cell sorting procedures. Second, we did not perform enrichment of myocardial cells, due to the limited sample from heart (apical) and beyond the scope of our present study. Similarly, we did not observe progenitor cells in the bone marrow sample, likely due to its rarity in bone marrow, which is reportedly less than 0.1% in bone marrow ([Alexey](javascript:;) *et al.，Blood* 2003, 102:94-101). Our future studies would focus on specific organ tissues, whereby we will have sufficient starting cells to reveal the heterogeneity of specific organs as well as spatial expression. We acknowledge our limitation in the **DISCUSSION** section, as read “we obtained around 5,000 cells in average for each organ, which might limit our ability to identify rare cell types and thus underestimate the heterogeneity of intercell interactions in organs.” Please kindly check in the last sentence in Page 22 to first sentence in Page 23.

**Response Figure 6.** **Violin plots showed the normalized expression of maker genes in different cell clusters in heart.** The marker genes from bottom to top are *CD14* (myeloid cells), *MS4A1* (B), *CD8A* (CD8^+^ T), *CD4* (CD4^+^ T), and *CD3E*(T/NK).

II. The authors should annotate each cluster in Figure S3B.

**Response:** Thanks for the suggestion. As suggested, we have annotated all the clusters identified in **Additional file 6: Figure S4B** (**Figure S3B** in the previous submission) in this revision. The corresponding markers and references were shown in the **Additional file 2: Table S3** in this revision.

III. What the different colors means in Figure 1C?

**Response:** Thanks for the question. In our previous submission, we had described in the figure legend that the colors of Figure 1C indicated the corresponding organs in Figure 1B. To make it clearer, we added the legend beside the **Fig. 1B** in this revision.

IV. Cell cluster annotations were confused in Figure 1C, why several 'Epithelial cell' existed?

**Response:** Thanks for the question. There are several different epithelial cell types from different organs, which were not specified in the previous submission. In this revision, we reannotated all the clusters and provided more precise cell type names for the clusters, of which the corresponding marker genes and references were shown in the **Addition file 2: Table S3** in this revision. We revised the labels of different cell clusters, including basal epithelial cell mainly in the trachea, squamous epithelial cells mainly in esophagus, and keratinocytes in the skin (**Fig. 1B**).

V. In figure S5, I do not know what is 'B Cells 1', 'B Cells 2', 'B Cells 3', what is the difference among them? How they distribute in various tissues? The same to other cells (such as cells in Figure 5C).

**Response:** Thanks for the questions. Sorry that we had not described the definition clearly. These B cells clusters are of different subtypes. In this revision, we reannotated B cell clusters and named different B cell subtypes as “B cell XXX_high”, where XXX indicates a marker gene with the most specifically high expression in the B cell subtype (**Additional file 8:** **Figure S6**). These subtypes now include “B Cell CD79A_high”, “B Cell HLA-DRA_high”, “B Cell MS4A1_high”, and “B Cell VPREB3_high”. “B Cell CD79A_high” and “B Cell MS4A1_high” contained B cells from multiple organs all over the body, while “B Cell HLA-DRA_high” and “B Cell VPREB3_high” were found mainly in the trachea and small intestine, respectively. Similarly, we have also annotated other cells and their distribution among tissues were provided as a supplementary Table (**Additional file 2: Table S20**), which is reproduced as below **Response Table 3**. For the cell clusters in **Fig. 5C**, we named the clusters with additional suffix representing their organ of origin. Their distribution among tissues were also provided as a supplementary Table (**Additional file 17: Table S36**), which is reproduced as below **Response Table 4**.

Please kindly check the relevant changes in **Additional file 8: Figure S6**, **Additional file 2: Table S20** and **Fig. 5C**, **Additional file 17: Table S36** in this revision).

**Response Table 3. Cell counts in each organ for each cluster indicated in Figure S6.**

| **Cell organ**  **cluster** | **Bladder** | **Blood** | **Common bile duct** | **Esophagus** | **Heart** | **Liver** | **Lymph.node** | **Marrow** | **Muscle** | **Rectum** | **Skin** | **Small intestine** | **Spleen** | **Stomach** | **Trachea** |
| --- | --- | --- | --- | --- | --- | --- | --- | --- | --- | --- | --- | --- | --- | --- | --- |
| **Absorptive Cell** | 0 | 0 | 0 | 0 | 0 | 0 | 0 | 0 | 0 | 2 | 0 | 268 | 0 | 0 | 0 |
| **B Cell CD79A_high** | 69 | 195 | 8 | 19 | 0 | 55 | 4837 | 469 | 7 | 576 | 10 | 19 | 1210 | 1050 | 69 |
| **B Cell HLA-DRA_high** | 0 | 0 | 0 | 0 | 0 | 0 | 0 | 0 | 0 | 0 | 0 | 0 | 0 | 0 | 142 |
| **B Cell MS4A1_high** | 2 | 2 | 0 | 1 | 0 | 3 | 157 | 54 | 0 | 19 | 0 | 1 | 59 | 48 | 4 |
| **B Cell VPREB3_high** | 0 | 0 | 0 | 0 | 0 | 0 | 0 | 0 | 0 | 0 | 0 | 75 | 0 | 0 | 0 |
| **Basal Cell KRT17_high** | 0 | 0 | 0 | 8 | 0 | 0 | 0 | 0 | 0 | 0 | 0 | 0 | 0 | 0 | 3235 |
| **Cholangiocyte** | 0 | 0 | 869 | 0 | 0 | 56 | 0 | 0 | 0 | 0 | 0 | 0 | 0 | 0 | 0 |
| **Endothelial Cell ACKR1_high** | 253 | 0 | 100 | 721 | 694 | 25 | 37 | 0 | 199 | 48 | 1121 | 4 | 0 | 17 | 820 |
| **Endothelial Cell FABP4_high** | 66 | 0 | 21 | 154 | 1205 | 57 | 8 | 0 | 1023 | 37 | 434 | 1 | 4 | 6 | 100 |
| **Enterocyte APOA1_high** | 0 | 0 | 0 | 0 | 0 | 0 | 0 | 0 | 0 | 0 | 0 | 1911 | 0 | 0 | 11 |
| **Enterocyte RBP2_high** | 0 | 0 | 0 | 0 | 0 | 0 | 0 | 0 | 0 | 0 | 0 | 1131 | 0 | 0 | 2 |
| **Epithelial Cell KRT5_high** | 0 | 0 | 0 | 400 | 0 | 0 | 0 | 0 | 0 | 0 | 194 | 0 | 0 | 0 | 1 |
| **Epithelial Cell TFF3_high** | 0 | 0 | 1 | 0 | 0 | 0 | 0 | 0 | 0 | 817 | 0 | 20 | 0 | 5 | 1 |
| **Erythrocyte/Mast/Langerhans Cell** | 64 | 12 | 8 | 8 | 8 | 11 | 16 | 91 | 5 | 28 | 77 | 1 | 40 | 13 | 33 |
| **Fibroblast APOD_high** | 0 | 0 | 0 | 11 | 0 | 0 | 0 | 0 | 853 | 0 | 3 | 0 | 0 | 0 | 0 |
| **Fibroblast PLA2G2A_high** | 173 | 0 | 43 | 68 | 15 | 0 | 0 | 0 | 0 | 60 | 8 | 0 | 0 | 11 | 641 |
| **Fibroblast PTGDS_high** | 216 | 0 | 36 | 927 | 56 | 1 | 2 | 0 | 12 | 61 | 697 | 0 | 0 | 17 | 58 |
| **Fibroblast PTN_high** | 5 | 0 | 0 | 12 | 4015 | 0 | 0 | 0 | 0 | 0 | 1 | 0 | 0 | 0 | 0 |
| **Fibroblast SFRP2_high** | 1894 | 0 | 11 | 2069 | 43 | 0 | 4 | 1 | 22 | 404 | 45 | 1 | 0 | 55 | 17 |
| **FibSmo PLAT_high** | 1730 | 0 | 6 | 1 | 0 | 0 | 0 | 0 | 0 | 44 | 0 | 0 | 0 | 24 | 0 |
| **FibSmo RGS5_high** | 0 | 0 | 0 | 0 | 141 | 0 | 0 | 0 | 2 | 0 | 0 | 0 | 0 | 0 | 0 |
| **FibSmo THBS4_high** | 12 | 0 | 0 | 13 | 0 | 0 | 0 | 0 | 0 | 724 | 0 | 0 | 0 | 3 | 0 |
| **Keratinocyte KRT1_high** | 0 | 0 | 0 | 0 | 0 | 0 | 0 | 0 | 0 | 0 | 2596 | 0 | 0 | 0 | 0 |
| **Keratinocyte KRT14_high** | 0 | 0 | 0 | 7 | 0 | 0 | 0 | 0 | 0 | 0 | 1915 | 0 | 0 | 0 | 0 |
| **Macrophage C1QB_high** | 416 | 2 | 91 | 267 | 497 | 436 | 5 | 36 | 46 | 674 | 46 | 26 | 111 | 13 | 91 |
| **Macrophage FCN3_high** | 13 | 0 | 4 | 23 | 225 | 292 | 6 | 0 | 40 | 10 | 3 | 0 | 0 | 0 | 8 |
| **Melanocyte** | 0 | 0 | 0 | 0 | 0 | 0 | 0 | 0 | 0 | 0 | 405 | 0 | 0 | 0 | 0 |
| **Doublets** | 75 | 0 | 0 | 91 | 268 | 0 | 0 | 0 | 17 | 19 | 15 | 0 | 0 | 0 | 7 |
| **Monocyte** | 343 | 100 | 418 | 19 | 52 | 725 | 10 | 113 | 99 | 340 | 34 | 3 | 213 | 80 | 244 |
| **Mucosal Epithelial Cell** | 128 | 2 | 2 | 4 | 0 | 3 | 0 | 1 | 0 | 2 | 66 | 0 | 0 | 626 | 69 |
| **Plasma Cell** | 6 | 88 | 1 | 99 | 2 | 18 | 27 | 45 | 3 | 713 | 1 | 28 | 373 | 574 | 29 |
| **Satellite Cell** | 0 | 0 | 0 | 0 | 0 | 0 | 0 | 0 | 2498 | 0 | 0 | 0 | 0 | 0 | 0 |
| **Schwann Cell** | 70 | 0 | 40 | 10 | 18 | 2 | 1 | 0 | 1 | 197 | 5 | 0 | 0 | 0 | 3 |
| **Secretory Cell** | 0 | 0 | 0 | 16 | 0 | 0 | 0 | 0 | 0 | 0 | 0 | 0 | 0 | 0 | 364 |
| **Smooth Muscle Cell** | 325 | 0 | 62 | 378 | 913 | 36 | 23 | 0 | 629 | 177 | 580 | 0 | 0 | 11 | 211 |
| **Squamous Epithelial Cell KRT13_high** | 0 | 0 | 0 | 2102 | 0 | 0 | 0 | 0 | 0 | 0 | 0 | 0 | 0 | 0 | 0 |
| **Squamous Epithelial Cell KRT15_high** | 0 | 0 | 0 | 2107 | 0 | 0 | 0 | 0 | 0 | 0 | 6 | 0 | 0 | 0 | 3 |
| **T Cell CCL4_high** | 1111 | 67 | 1174 | 45 | 43 | 769 | 434 | 1187 | 157 | 365 | 27 | 7 | 964 | 279 | 162 |
| **T Cell CCL5_high** | 0 | 0 | 0 | 0 | 0 | 1 | 0 | 0 | 0 | 0 | 0 | 0 | 0 | 1 | 1365 |
| **T Cell DLK1_high** | 0 | 0 | 0 | 0 | 0 | 0 | 0 | 0 | 32 | 0 | 0 | 0 | 0 | 0 | 0 |
| **T Cell GNLY_high** | 100 | 26 | 73 | 3 | 8 | 315 | 4 | 605 | 307 | 21 | 14 | 1 | 422 | 37 | 98 |
| **T Cell GZMA_high** | 27 | 0 | 70 | 3 | 0 | 13 | 16 | 12 | 0 | 577 | 2 | 39 | 60 | 2223 | 60 |
| **T Cell IL7R_high** | 489 | 987 | 288 | 11 | 36 | 170 | 2603 | 786 | 49 | 775 | 24 | 14 | 1294 | 500 | 68 |
| **T Cell RGS1_high** | 0 | 0 | 0 | 0 | 0 | 0 | 0 | 0 | 0 | 0 | 0 | 748 | 0 | 1 | 2 |
| **Tuft Cell** | 0 | 0 | 0 | 0 | 0 | 0 | 0 | 0 | 0 | 5 | 0 | 241 | 0 | 4 | 0 |

**Response Table 4. Cell counts in each organ for each cluster indicated in Fig. 5C.**

| **Cell organ**  **cluster** | **Bladder** | **Common bile duct** | **Esophagus** | **Liver** | **Rectum** | **Skin** | **Small intestine** | **Stomach** | **Trachea** |
| --- | --- | --- | --- | --- | --- | --- | --- | --- | --- |
| **Epithelial Cell S100P_high Bladder** | 117 | 1 | 0 | 1 | 0 | 0 | 0 | 0 | 1 |
| **Cholangiocyte FXYD2_high Common.bile.duct** | 0 | 722 | 0 | 6 | 0 | 0 | 0 | 0 | 0 |
| **Cholangiocyte MUC5B_high Common.bile.duct** | 0 | 112 | 0 | 0 | 0 | 0 | 0 | 0 | 0 |
| **Squamous Epithelial Cell DST_high Esophagus** | 0 | 0 | 1102 | 0 | 0 | 0 | 0 | 0 | 0 |
| **Squamous Epithelial Cell KRT13_high Esophagus** | 0 | 0 | 949 | 0 | 0 | 0 | 0 | 0 | 0 |
| **Squamous Epithelial Cell FABP5_high Esophagus** | 0 | 0 | 945 | 0 | 0 | 2 | 0 | 0 | 0 |
| **Squamous Epithelial Cell KRT4_high Esophagus** | 0 | 0 | 700 | 0 | 0 | 0 | 0 | 0 | 0 |
| **High Proliferate Epithelial Cell Esophagus** | 0 | 0 | 445 | 0 | 0 | 0 | 0 | 0 | 0 |
| **Epithelial Cell ALB_high Liver_Common.bile.duct** | 0 | 22 | 0 | 49 | 0 | 0 | 0 | 0 | 0 |
| **Simple Epithelial Cell Rectum** | 0 | 0 | 0 | 0 | 534 | 0 | 0 | 3 | 0 |
| **Goblet Cell Rectum** | 0 | 0 | 0 | 0 | 222 | 0 | 26 | 1 | 1 |
| **Spinous Epithelial Cell KRT1_high Skin** | 0 | 0 | 0 | 0 | 0 | 987 | 0 | 0 | 0 |
| **Spinous Epithelial Cell KRT10_high Skin** | 0 | 0 | 0 | 0 | 0 | 856 | 0 | 0 | 0 |
| **Follicular Epithelial Cell Skin** | 0 | 0 | 1 | 0 | 0 | 640 | 0 | 0 | 0 |
| **Basal Epithelial Cell KRT14_high Skin** | 0 | 0 | 0 | 0 | 0 | 633 | 0 | 0 | 0 |
| **Basal Epithelial Cell APOE_high Skin** | 0 | 0 | 4 | 0 | 0 | 525 | 0 | 0 | 0 |
| **Spinous Epithelial Cell KRTDAP_high Skin** | 0 | 0 | 0 | 0 | 0 | 401 | 0 | 0 | 0 |
| **High Proliferate Epithelial Cell Skin** | 0 | 0 | 0 | 0 | 0 | 265 | 0 | 1 | 0 |
| **Granular Epithelial Cell SKin** | 0 | 0 | 0 | 0 | 0 | 127 | 0 | 0 | 0 |
| **Epithelial Cell DCD_high Skin_Esophagus** | 0 | 0 | 40 | 0 | 0 | 72 | 0 | 0 | 0 |
| **Spinous Epithelial Cell RORA_high Skin** | 0 | 0 | 0 | 0 | 0 | 75 | 0 | 0 | 0 |
| **Enterocyte RBP2_high Small.intestine** | 0 | 0 | 0 | 0 | 0 | 0 | 1169 | 0 | 3 |
| **Enterocyte APOA1_high Small.intestine** | 0 | 0 | 0 | 0 | 0 | 0 | 1156 | 0 | 5 |
| **Enterocyte PCK1_high Small.intestine** | 0 | 0 | 0 | 0 | 0 | 0 | 656 | 0 | 5 |
| **Tuft Cell Small.intestine** | 0 | 0 | 0 | 0 | 2 | 0 | 249 | 4 | 0 |
| **Absorptive Cell Small.intestine** | 0 | 0 | 0 | 0 | 0 | 0 | 180 | 0 | 0 |
| **Stem Cell Small.intestine** | 0 | 0 | 0 | 0 | 0 | 0 | 69 | 0 | 0 |
| **Pit Mucosal Epithelial Cell Stomach** | 0 | 0 | 0 | 0 | 0 | 0 | 0 | 420 | 0 |
| **Chief Cell Stomach** | 0 | 1 | 0 | 0 | 0 | 0 | 0 | 39 | 0 |
| **Basal Epithelial Cell MMP10_high Trachea** | 0 | 1 | 1 | 0 | 0 | 0 | 0 | 0 | 2215 |
| **Basal Epithelial Cell SERPINB3_high Trachea** | 0 | 0 | 0 | 0 | 0 | 0 | 0 | 0 | 857 |
| **Secretory Epithelial Cell Trachea** | 0 | 0 | 17 | 0 | 0 | 0 | 0 | 0 | 360 |
| **Ionocyte Trachea_Small.intestine** | 0 | 0 | 0 | 0 | 2 | 0 | 23 | 9 | 59 |

VI. There was no any Tfh or Th in Figure 2A?

**Response:** Thanks for the question. T follicular helper (Tfh) cells are marked by the expression of *CXCR5*, *BCL6* and *PDCD1*, providing requisite help to B cells in the germinal centers (GC) of lymphoid tissue (Vella *et al., J Clin Invest* 2019, 129:3185-3200). We did not observe *CXCR5*^+^*BCL6*^+^*PDCD1*^+^ CD4^+^ T cells cluster in our study (**Response Figure 7**). We suspected that the recovery rate of Tfh cells was very low because Tfh cells are susceptible to nicotinamide adenine dinucleotide (NAD)-induced cell death (NICD) during isolation (Künzli *et al., Sci Immunol. 2020, 5(45): eaay5552*). According to the previous report, additional injection of NICD protector before organ harvest is critical to improve the recovery rate of Tfh cells.

We identified a small group of effect CD4^+^ T cells, NKG7_T_EFF_ (10) in our previous submission. The cluster of cells were highly enriched with several TFs including *TBX21*, *IFR1* and *STAT1* (**Fig. 2D**), which are Th1 cell markers (Zhu *et al., Annu Rev Immunol* 2010, 28:445-489). Thus, we inferred NKG7_T_EFF_ (10) as Th1 cells in this revision. We did not observe Th2 and Th17 cells in our data, which is likely due to the low number of the T_EFF_ CD4^+^ T cells (146) in our study and low abundance of Th2 (1.82%) and Th17 (0.52%) in CD4^+^ T cells according to the previous report (Chen *et al., Mol Med Rep* 2015, 12:6105-6111).

**Response Figure 7. Violin plot showed the normalized expression of marker genes in T cell clusters.** T follicular helper cell (Tfh: *CXCR5* and *PDCD1; GRS10, FOS, HIF1A* and *CD27,* by Künzli *et al.*); T_N_/T_CM_ (*SELL*, *TCF7*, *CCR7*, and *LEF1*).

VII. There was no any mast cells in a healthy donor and no DC in the lymph node (Figure S2)?

**Response:** Thanks for the question. We only observed 19 mast cells clustered in one group in the trachea (**Additional file 4: Figure S2 continued)**. We did not observe DC cell populations in the lymph node. Future study with sufficient number of cells from lymph node would be necessary in order to capture a considerable number of DC, since DC cells comprise about 0.2% of all CD45^+^ cell in the lymph node (Granot *et al., Immunity* 2017, 46:504-515).

5) Authors claim to identify rare and novel cell populations (Langerhans cells, COCH+ fibroblast, and SCGB2A2+ epithelial cells), however, they did not show their tissue distribution. Did those three clusters exist in Figure S3B? And Langerhans cell is not a novel cell type. Where is the Langerhans cell in Figure 4A?

**Response:** Thanks for the questions. We defined those cell populations with poor characterization or low abundance in the previous literatures as rare and/or novel cell types. To address the Reviewer’s comment, we added a figure in the **Additional file 5: Figure S3B** in this revision to show the tissue distribution of Langerhans cells, *COCH*^+^ fibroblasts, *SCGB2A2*^+^ (sweat gland) epithelial cells, and FibSmo cells. Please note that we have specified the *SCGB2A2*^+^ epithelial cells as “sweat gland epithelial cell” in this revision, as the cells expressed a marker gene (*DCD*) of sweat gland epithelial cells. We agree with the Reviewer that Langerhans cell is not a novel cell type, which is a rare cell type in the skin and plays an important role in antimicrobial immunity (Merad *et al., Nat Rev Immunol* 2008, 8:935-947; Clayton *et al., Front Immunol* 2017, 8:1676). The other three cell types are yet to be characterized. *COCH*^+^ fibroblasts and novel FibSmo cells were found in multiple organs, with various proportions (**Additional file 5: Figure S3B**). By contrast, Langerhans and *SCGB2A2*+ sweat gland epithelial cells were found specifically in skin (**Additional file 5: Figure S3B**).

In **Additional file 6: Figure S4B** (corresponding to previous **Figure S3B**), Langerhans cell, *COCH*^+^ fibroblasts, and sweat gland epithelial cells were grouped in the cluster 34 “Erythrocyte/Mast/Langerhans cell”, the cluster 17 “Fibroblast PTGDS_high”, and the cluster 36 “Secretory Cell”, respectively. For the FibSmo cells, three clusters were found, including “FibSmo PLAT_high (22)”, “FibSmo THBS4_high (30)”, and “FibSmo RGS5_high (43)”. Langerhans cell is the cluster 16 skin cells in **Fig. 4A**, and now assigned as CD207_DC as in **Fig. 4C**.

We have also updated the relevant descriptions in the main text and Figure legends. Please kindly check in Paragraph 2, Page 6 in the **RESULTS** section, and the figure legend of **Figure S4**.

6) Figure legends were careless and ambiguous. There was no annotation for color in each heatmap and trajectory results, the simple annotations should exist in figures.

**Response:** Sorry for that. In this revision, we added annotations for colors in each figure and corresponding legends for better understanding the results.

7) The authors claimed that 'various TFs regulate the development of B cells to plasma cells.' That is lack of evidence, they should provide trajectory analyses just like CD4/CD8 T cells.

**Response:** Thanks for the suggestion. In the beginning of our study, we had tried trajectory analyses for B and plasma cells using monocle 2.99, with different key parameter combinations, including “mean expression” and “dispersion ration”. However, we did not observe a link of the trajectories between B cell and plasma cells, which are separated into two trajectory networks for B cell and one for plasma cell instead (Below **Response Figure 8**). A possible explanation might be that cells with transitional state between memory B cells and terminal plasma cells were not well captured. Nevertheless, the key TFs in the development of plasma cell have been well characterized previously, such as *PRDM1*, *XBP1*, *MYC* and *REL* (Shinnakasu R *et al., Curr Opin Immunol* 2017, 45:126-131). Consistently, we also observed these TFs in the development of B cells to plasma cells in our study (**Fig. 3E**), as well as in the analysis using a recent single cell data (Han *et al., Nature* 2020, 581:303-309) in **Additional file 13: Figure S9F** in this revision. Taken together, these observations suggest that these TFs might regulate the development of B cells to plasma cells.

We have updated the relevant results in the main text. Please check in Paragraph 1, Page 11 in the **RESULTS** section.

**Response Figure 8. Pseudo-time trajectory analysis of all B and plasma cells with high variable genes.** **A.** trajectory paths in cell clusters in different colors; **B.** t-SNE plot at each plot shows each cell with a pseudo-time score from dark blue to yellow, indicating early and terminal states, respectively. Each dot represents one cell.

8) The origin of Mac is ambiguous; the authors should claim which Mac come from monocytes and which from proliferation cells.

**Response:** Thanks for the suggestion. First, we observed the coexistence of macrophages and monocytes in same organs (**Fig. 4A, B, Additional file 14: Figure S10A**). Second, trajectory analysis revealed developmental trajectory from monocytes to macrophages either directly or via the two intermediate monocytes (**Fig. 4E and Additional file 14: Figure S10B, C**). Lastly, high expression of proliferation markers including *MKI67*, *PCNA* and *TYMS* were exclusively detected in the C12 macrophages in bladder, common bile duct, esophagus, heart, and rectum (**Additional file 14: Figure S10D**). Taken together, these suggest that circulating monocytes might give rise to macrophages in all tested organs and the local microenvironment also contributes to the expansion and proliferation of different macrophage populations, especially tissue-resident macrophages, such as in the bladder, common bile duct, esophagus, heart, and rectum.

We have updated the information in the main text. Please kindly check from Paragraph 2, Page 12 to Paragraph 1, Page 13 in the **RESULTS** section.

9) The authors should provide a volcano figure for DEGs between two groups (13 digestion-related clusters vs. 20 non-digestion-related clusters) before Figure 5D.

**Response:** Thanks for the suggestion. As suggested, we added a volcano plot for the DEGs between 13 digestion-related clusters and 20 non-digestion-related clusters in **Fig. 5D** in this revision. Significant DEGs were determined between the two groups, including 472 upregulated genes with fold change larger than two (268 in the digestion-related clusters and 204 in the non-digestion-related clusters). The relevant results were added to the main text. Please kindly check in Line 6-8, Paragraph 1, Page 15 in the **RESULTS** section.

10) What are the 'FibSmo' cells? The authors should describe what they are, and are they doublets or novel type cells? If they are a new type of cells, the authors should confirm their existent with experiment.

**Response:** Thanks for the thoughtful suggestions. FibSmo is a novel type of stromal cells identified in multiple organs, with co-expression of *MMP2* and *ACTA2* (**Additional file 20: Figure S14A, B**). There are 3,182 FibSmo cells, distributing mainly in rectum, bladder, and heart (**Additional file 5: Figure S3B** and **Additional file 20: Figure S14A, B**). As suggested, we confirmed the presence of FibSmo cells in the bladder, heart and rectum from two independent donors, using immunofluorescence staining with marker genes MMP2 and ACTA2 (**Additional file 6:** **Figure S4A**).

We now update the new data in our revision. Please kindly check in Line 8-18, Paragraph 2, Page 6, and Line 11-15, Paragraph 2, Page 17, in the **RESULTS** section.

11) Problems in data analysis.

I. The gene expression matrix was obtained with CellRanger (version 3.0.1), while TCR/BCR contigs were generated with CellRanger (version 3.1.0). Why the authors chose different version?

**Response:** Thanks for the question. We initially performed all analyses using CellRanger version 3.0.1, including generating the digital expression counts matrix and TCR/BCR clone identification. Recently, we realized that a new version 3.1.0 was released, with substantial changes of algorithms to improve the identification of TCR/BCR clonotypes (“cellrancger vdj” module). Therefore, we used version 3.1.0 for TCR/BCR identification in our study, which we noted in the **METHODS** section. For gene expression, we used version 3.0.1, because we had generated the results and the change to version 3.1.0 has minimal impact on the gene expression according to the Release Notes ([https://support.10xgenomics.com/single-cellgeneexpression/ software/pipelines/ latest/release-notes](https://support.10xgenomics.com/single-cellgeneexpression/%20software/pipelines/%20latest/release-notes)).

II. The authors should use the 'NormalizeData' function before the 'ScaleData' function.

**Response:** Thanks for the suggestion. In fact, we had performed the 'NormalizeData step' before the 'ScaleData' function but did not describe this in the previous submission. In this revision, we provided a more detailed summary of the methods, as read “From the remaining cells, gene expression counts data for each sample was subtracted and normalized with “NormalizedData” function, followed by scaling to regress UMIs and mitochondrial content using “ScaleData” function (negative binomial model)” Please kindly check the **METHODS** section in the last sentence in Page 25. In addition, the relevant R scripts were uploaded to GitHub via <https://github.com/bei-lab/scRNA-AHCA>, where the clustering R scripts was in “3. Clustering” folder.

III. When the authors identified contaminated genes, they just randomly sampled one time. They should be cautious to randomly sample and set the cutoff, because that 10X-dataset has high dropout ratio, especial for the genes with low expression.

**Response:** Thanks for the suggestion. We agreed with the Reviewer that 10X-dataset has high dropout ratio. As suggested, we performed four independent calculations to identify contaminated genes in this revision, with seed numbers 1 to 4 in the ‘FindMarker’ function to randomly sample the cells from each organ. A same gene set was detected in each sampling, likely because all the contaminated genes were those with the highest expression in each organ. The genes were listed in **Additional file 25: Table S52-56**. The detailed R script named as “Remove_the_contaminated _genes.R” could be found at GitHub (<https://github.com/bei-lab/scRNA-AHCA>). We have updated these in the **METHODS** section. Please kindly check in Line 5-8, Paragraph 1, Page 27.

IV. The authors used the MHC II genes to measure the 'Presenting-antigen ability', how about the MHC I genes?

**Response:** Thanks for the question. In the previous submission, we focused on MHC II genes, considering the expression of MHC II genes in non-antigen-presenting cell types and the detailed heterogeneity of antigen-presenting ability in subpopulation of different major cell types are relatively unclear compared to MHC I genes. Following the suggestion, we analyzed the 'Presenting-antigen ability' using the MHC I genes in the term of ‘REACTOME_CLASS_I_MHC_MEDIATED_ANTIGEN_PROCESSING_ PRESENTATION’ for different cell types.

We observed heterogeneous antigen-presenting abilities for intracellular (MHC I) and extracellular antigens (MHC II) among different cell types (Below **Response Figure 9 A-D**). For professional antigen-presenting cells including myeloid and B cells, most of cell clusters had higher antigen-presenting score for extracellular antigens (MHC II) (**Response Figure 9A-B**). By contrast, most non-professional antigen-presenting cells had higher score for presenting intracellular antigens (MHC I) (**Response Figure 9C-D**). Interestingly, cells from same lineage in different development sate had different antigen-presenting ability. B cells had higher score to present extracellular antigens (MHC II) than plasma cells, while plasma cells had higher ability to present intracellular antigens (MHC I; **Response Figure 9A**).

We have included the results in our manuscript. Please kindly check in **RESULTS** section: Line 1-6, Paragraph 2, Page 10; Line 2-5, Paragraph 3, Page 13; Line 1-3, Paragraph 1, Page 14; the last sentence of Paragraph 2, Page 15; and Line 4-8 Paragraph 1, Page 17.

**Response Figure 9.** **Boxplot showed the antigen-presenting ability scores for intra- or extracellular antigen of major cell types including B and plasma cell (A), myeloid cell (B), epithelial cell (C), and endothelial cell (D).** Score was calculated using intra- or extracellular antigen-presenting associated genes in the REACTOME database ‘REACTOME_CLASS_I_MHC_MEDIATED_ANTIGEN_PROCESSING_ PRESENTATION’ and ‘MHC CLASS II ANTIGEN PRESENTATION’ pathway, respectively. The red box labeled as MHC I presented scores for presenting intracellular antigens, and the blue one labeled as MHC II was for extracellular antigens scores.

Minor problems:

1) This manuscript is suspected of rubbing hot spots, what's the relationship of this manuscript and the COVID-19? There were no alveolar cells in their dataset.

**Response:** Thanks for the comment. We now delete this section in the revision. We fully understand that our results related to COVID-19 are very preliminary. However, we want to assure that we do not have any intention to attract the spotlight of COVID-19. It is an intrinsic responsibility as scientists to contribute any kinds of supports that might potentially help contain the pandemic COVID-19. Indeed, we started this project before the outbreak of COVID-19. During the outbreak, we noticed more and more publications regarding the receptor of coronavirus and the complications especially the acute cardiac injury, therefore, we thought that it might be important to provide a landscape of the ACE2 expression in human organs, especially heart tissue. We are pleased to see that many related findings have been published recently and thus we would like to remove this section.

2) Are their countries different in address 1 and address 3? (P. R. China VS China)

**Response:** Thanks a lot for pointing out this overlooked mistake. Both addresses are from P.R. China. We now provide the correct expression for all addresses in this revision.

3) A figure should be used to show the sequencing saturation, instead of a table (Additional file 20: Table S2).

**Response:** Thanks for the suggestion. We now present a figure to show the sequencing saturation as suggested. Please kindly check **Figure S1 A** in this revision.

4) Why the 'Number of Read Pairs' in Table S2 among tissue is so different (nearly 4-fold higher)?

**Response:** Thanks for the question. These are read pairs for TCR and BCR in each organ sample. We used a pooling strategy to prepare sequencing library, whereby TCR, BCR and 5’-cDNA libraries were pooled together and subjected for sequencing in one HiSeq lane. We aimed to obtain 7.5G TCR, 4.5G BCR, and 108G 5’-cDNA data for 5,000 T cells, 3,000 B cells and 8,000 all cells, respectively in one lane with 110Gb data. T cells have higher proportions than B cells in most organs, except for spleen (Wong *et al.,* *Immunity* 2016, 45:442-456). For spleen sample, we aimed at 6G data for 4,000 B and T cells. However, variance would be introduced during pooling the libraries, especially in the case that BCR and TCR data only compose 5-10% of a single HiSeq XTen lane. Therefore, we obtained various TCR and BCR reads beyond our expectation. Nevertheless, we obtained sufficient TCR and BCR data, with an average of 5,000 paired reads for a T or B cell, and thus the various read pairs had no impact on the downstream TCR and BCR analyses. Please note that we have reordered the previous **Table S2** to **Additional file 2:** **Table S1** in this revision.

5) Authors claim to identify 91,393 cells after QC filter, what's the number of total cells before QC?

**Response:** Thanks for the question. Initially, we loaded 14,000 cells in each channel for each organ, targeting at a recovery of 5,000-10,000 cells for each organ. Output cells for each organ were forced to 13,000 under ‘cellranger count’ module before QC. These added up to 195,000 inferred cells in 15 organs before QC. For QC, we removed cells with low quality (UMI < 1,000, gene number < 500, and mitochondrial genome fragments ≥ 0.25) for each organ, resulting in 91,393 for all organs as shown in **Additional file 1: Figure S1B** in this revision. We have added the information in **METHODS** section. Please kindly check Line 1-3, Paragraph 1, Page 25 and Line 4-6 Paragraph 3, Page 25.

6) The figure S1 should be separated into two parts to show 'UMI' and 'gene' respectively.

**Response:** Thanks for the suggestion. As suggested, we presented UMI and genes in each organ using two separated bar plots in **Additional file 1: Figure S1C-D** in this revision (**Figure S1** in the previous submission).

7) What was the expression cutoff for 'genes with high expression', and how many genes used in Figure S4? I think correlation value should range from -1 to 1, instead of -4 to 4, and authors should provide the statistical P value for the correlation in Figure S4.

**Response:** Thanks for the questions. In **Figure S4** in the previous submission, which is now **Additional file 7: Figure S5** in this revision, we intended to provide an overall view of the cell numbers for each cell type in different organs, using a similar method described previously (The Tabula Muris Consortium *et al., Nature* 2018, 562:367-372). The x and y axes indicated cell types and specific cell type in organs, respectively. The heatmap indicated the log2 (cell number) in this revision, which was normalized cell number of each row in previous submission. We did not compare the gene expression for these cells here.

To avoid confusion, we indicated the variable “log2(cell number+1)” and value range from 0-12 in the heatmap scale at the bottom of **Figure S5**, as well as relevant description in the figure legend.

8) The authors should provide cell number of each cluster in each tissue in Figure S6A.

**Response:** Thanks for the suggestion. As suggested, we provided cell numbers of each cell cluster (CD4/CD8) in each organ tissue in **Additional file 10: Figure S7A** in this revision (**Figure S6A** in the previous submission). In addition, we also provided such information for other similar figures throughout the manuscript in this revision, including **Fig. 2A, B, Fig. 3A, Fig. 4A, Fig. 5A, Figure S9E, Figure S11B, Figure S14A, and Figure S15A**.

9) The authors claimed that 'we preformed TCR clonal typing accompanied with transcriptome analysis (Fig. 1E).' where is the Figure 1E? they also claimed that 'we observed consistent results of TCR diversity using β chains (Additional file 8: Figure S7J-I).' I think it was Figure S7J-L.

**Response:** Thanks a lot for pointing out the typos. We are sorry for the overlook typos. The **Fig. 1E** should be **Fig. 1A**. **Figure S7J-I** should be **Figure S7J-L** as mentioned by the Reviewer. We have corrected these typos in this revision. Please note that **Figure S7 J-L** has been reordered as **Additional file 11: Figure S8 J-L** in this revision.

10) What did each row present in Figure S7H? what did the size mean in Fig. 2F and Fig. 3F?

**Response:** Thanks for the questions. Each row in the **Figure S7H** (reordered as **Additional file 10: Figure S8H** in this revision) presented a CD4^+^ T cell cluster, which was added in the figure in this revision. For **Fig. 2F** and **3F**, the size of the circles represented the exact count of the cell clones in indicated organ. Please note that we used logarithm to the base 2 of the count in this revision. We have updated the information in the corresponding figure legend.

11) There was no legend for Figure 3B in the figure. (Just like Figure 5B)

**Response:** Thanks for pointing out this overlook. We provided the legend for **Fig. 3B**. However, the space around **Fig. 5B** is not enough for additional legend, thus we used the same figure legend with **Fig. 5A** in this revision. We have updated that in the figure legends.

12) What is the cutoff for DEGs among B cells? There were so many gene with P > 0.05 in Table S5.

**Response:** Thanks for the question. In the previous submission, we kept DEGs among B cells if any of them met ln(fold change) greater than 0.25. In this revision, we further filtered the remaining DEGs with *p.adjust* value less than 0.05. Please note that the new table is now **Additional file 12: Table S28**.

13) There were many TFs in monocyte-macrophage development in this manuscript, such as NFIL3, RXRA, ELF2, NR1H3, SPIC and HDAC2. The expression of those genes should be presented with figures.

**Response:** Thanks for the suggestion. We provided the expression of some key TFs in different monocyte-macrophage development state in **Fig. 4G** in this revision, including *RXRA*, *ELF2*, and *NFIL3* for classical monocyte, *STAT1* and *TCF7L2* for both non-classical monocyte and LIPA_Mac macrophage, *JUN* and *PRDM1* for most macrophage clusters.

14) It is an arbitrary decision to name the cell clusters, the column names of Figure S14 are different from cluster names (such as Figure S11D).

**Response:** Thanks for the comment. We aimed to identify the interaction pairs of molecules between two cell types. In order to present our results better, we reduced pairwise combinations by grouping the specified cell clusters into major cell types, including CD4^+^ T cell (T_N_, T_CM_, Treg, T_EM_, T_h1_, and T_RM_), CD8^+^ T cell (T_N_, T_CM_, T_EM_, T_EFF_, T_RM_, IEL, γδ, and MAIT), B cell (naïve and memory B cells), NK cell, plasma cell, myeloid cell (monocyte: Mon, macrophage: Mac, and dendritic cell: DC), endothelial cell (BEC and IEC), fibroblasts (Fib), smooth muscle cell (Smo), and FibSmo cell (FibSmo). As such, we used different column names of **Figure S14** in the previous revision (now **Additional file 26: Figure S18** in this revision). We provided relevant description in the **METHODS** section. Please kindly check in Lines 1-8, Paragraph 1, Page 29.

15) In Figure 6C, each figure presents a tissue?

**Response:** Thanks for the question. No, each figure did not present a tissue. Each figure presents a major cell type as indicated in the subtitles and the number of significant ligand-receptor interactions between the major cell type and other major cell types as indicated in the colored circles. These descriptions were provided in the figure legend. Please note that we moved the figure to supplementary as **Additional file 24**: **Figure S17A-C** in this revision.

16) In Figure S15, S16, and S17, the '-pvalue' is 1.5? maybe '-logP'.

**Response:** Thanks for pointing out this mistake. In this revision, we corrected “-pvalue” as “-log10*P-value*” in **Figure S18-21** (**Figure S14-18** in the previous submission).

17) It is better to add interactions among various tissues.

**Response:** Thanks for the suggestion. We have performed interaction analysis among all major cell types across different organs. We observed broad interactions with different density among various organ tissues (**Fig. 6D, E**, **Additional file 25:** **Table S48**). We note that cells in small intestine had high density of interaction pairs with multiple tissues including heart, esophagus and lymph node, suggesting potential regulatory communications across these organs. Interestingly, myeloid, CD8^+^ T, and epithelial cells were the core nodes of cell-cell interactions, which had the most interacting pairs and enhanced pairwise communications (**Fig. 6E**, **Additional file 25:** **Table S48**).

**Reviewer #2:**

Summary:
He et al have compiled a single-cell RNA-seq and VDJ-seq dataset including 15 different organs from a single individual. The authors describe cell types and patterns of B and T cell clone distributions across organs. While many of the findings are expected, the authors make some interesting observations, however, the lack of replicates and experimental validation weakens any conclusion regarding novel and/or rare cell types.

**Response:** Thanks for the positive comment. In this revision, we have provided additional analyses and experimental validation for the identification of novel and/or rare cell types, which could be found below as to address the Reviewer’s comments.

Major comments:

1) The provided figures were in illegible quality.

**Response:** We are sorry for that. We assure that the resolution of all figures in this revision should meet sufficient quality. If there is any case that the submission system reduces file size by lowering down the quality of a figure in a page, we should see a link- “Click here to access/download” at the top right of the page. We would be able to download the original file that were uploaded to the system for the figure.

2) This study has profiled only one donor. Given the high biological variation in humans, the applicability of these findings to the broader population are under question.

**Response:** Thanks for the pertinent comment. We fully understand the Reviewer’s concern if our findings could be applied to broader population in the context of biological variations among individuals. In our study, thousands of cells from multiple organs were analyzed, which enables us to understand the heterogeneous composition of cells in human organs and the development links among cell types, and to identify important molecules and their networking. Such as, similar with the report by The Tabula Muris Consortium *et al.* (*Nature* 2018, 562:367-372), we observed close clustering of cells from different organs (more than seven organs) for major cell types, including T cells, B cells, NK cells, fibroblasts, endothelial cells, smooth muscle cells, macrophages, and monocytes. We also identified several well characterized TFs regulating the different cell development, including *TCF7*, *SELE*, *MYC*, and *KLF2* for T_N_ cells, *TBX21*, *STAT1*, and *IRF1* for T_EFF_ cells, and *PRDM1*, *XBP1*, *FOS*, and *IRF4* for plasma cells. The consistencies between our findings and previous findings suggest that our study based on large scale transcriptomes at single cell level provides common biological understanding at higher resolution.

Moreover, we used an independent set of single cell sequencing data derived from human organs, which was published very recently (Han *et al. Nature* 2020:581, 303-309), and we managed to replicate our findings. First, we identified important transcription factors regulating plasma cell development, such as *XBP1*, *MYC*, etc., which have similar activation patterns in both datasets (**Fig. 3E** and **Figure S9F**). Second, epithelial cells from different organs showed strong heterogeneity, while epithelial cells within the same tissue had similar gene expression profile and clustered together, in both datasets (**Fig. 5A** and **Figure S11B**). Third, we found that both digestive and non-digested tissues showed similar transcriptional factor activities in both datasets (**Fig. 5G and Additional file 19: Figure S13B**). In addition, we note that we only selected B cells and epithelial cells for replication analyses, because the recent published data has low sequencing depth and extensive contamination between different cell types (Han *et al., Nature* 2020:581, 303-309).

Furthermore, we validated our discovery of novel and/rare cell types using experiment as suggested by the Reviewer #2, including sweat gland epithelial cell in skin, *COCH*^+^ fibroblast in the skin and heart, and FibSmo in the bladder, heart, and rectum in independent samples (**Figure S3C** and **Figure S4A**). Please check more details regarding this at below Reviewer’s comment point 4 (**Response Page 38**).

Taken together, although our data was derived from one donor, some important findings based on large number of cells and multiple organs might be generally applicable. However, we do acknowledge that further investigations with more individual samples will solidify our findings and discover more novel cell types, key molecules, and interaction network.

We have added the replication analyses and experimental results as well as limitation in this revision. Please kindly check in the **RESULTS** section in Paragraph 2, Page 6; Paragraph 1, Page 11; Line 4-7, Paragraph 2, Page 14; Line 8-13 Paragraph 3, Page 14; Line 3-6, Paragraph 1, Page 15; Line 6-12, Paragraph 2, Page 15; and Line 5-9, Paragraph 1, Page 16.

3) Authors overinterpret the detection of higher nUMIs in skin and trachea samples as having "higher transcriptional activation". This is impossible to know without having technical replicates and unlikely to be the case for all cell types in one organ.

**Response:** Thanks for the comment. We agreed with the Reviewer’s opinion and revised the result, as read “Higher number of UMIs and more transcribed genes were observed in the skin and trachea samples (with median UMIs of 4,022.5 and 4,100.5 and genes of 1,528 and 1,653, respectively) compared to the other organs **(****Additional file 1: Figure S1C, D, and Additional file 2: Table S2**). Please kindly check in the **RESULTS** section in Line 12-15, Paragraph 3, Page 5.

4) The identification of rare/novel cell types (e.g. Langerhans cells and COCH+ fibroblast subtypes) should be supported by replicates/validation.

**Response:** Thanks for the comment. To address the comment, we performed immunofluorescence staining assays to confirm the presence of rare/novel cell types identified in our study. We managed to locate sweat gland epithelial cell in two independent skin samples, which were stained with makers KRT19, SCGB2A2, and PIP (**Additional file 5: Figure S3C**). Similarly, we validated the novel fibroblast subtype by staining markers COCH and MMP2 in the skin and heart samples from two independent individual donors (**Figure S3C**). Importantly, we confirmed the novel cell type FibSmo with markers MMP2 and ACTA2 in the bladder, heart, and rectum samples from two independent individual donors (**Figure S4A**). Please note that we don’t examine the presence of Langerhans cell in tissue samples, because this cell type has been widely reported (Merad *et al., Nat Rev Immunol* 2008, 8:935-947; Clayton *et al., Front Immunol* 2017, 8:1676).

We have added the above results in this revision. Please kindly check in Line 15-18, Paragraph 2, Page 6 in the **RESULTS** section.

5) The authors should provide a complete description of the tissue dissociation method. In addition, they should comment on how the use of different dissociation protocols could result in selective release of cells in different organs and/or induction of stress response.

**Response:** Thanks for the suggestions. As suggested, we now provide a detailed description of tissue dissociation protocols in **Additional file 30**, which includes the procedures as well as the use of enzymes and treatment durations for each organ. We note that these disassociation protocols were established based on the previous publications and rounds of in-house tests on different pig and human tissues until we could achieve high cell viability, less digestive time and less residual tissue.

As pointed out by the Reviewer, cellular dissociation was a prerequisite technical manipulation for single cell studies, although dissociation procedures would cause disturbance of transcriptome at various degrees (Brink *et al.,* *Nat Methods* 2017, 14:935-936). Enzymes and duration of dissociation procedures might be two important factors (Harris *et al., Hippocampus* 2019, 29:876-882; Brink *et al.,* *Nat Methods* 2017, 14:935-936). A gentle enzyme and short time might help to minimize the disturbance of transcriptome or induction of stress response. Moreover, an organ consists different cell types and extra cellar matrix, which need different enzymes to dissociate them into single cells. It’s unlikely that one enzyme could fit for all organs or cell types. Long duration and high concentration of enzymes could have a higher degree of dissociation. However, it comes with higher level of damage on cells and disturbance of transcription. Collagenase could only degrade the triple-helical native collagen fibrils; Dispase cleaving fibronectin, type IV collagen and type V collagen are gentle and have limited damage to cells; and Trypsin could cleave polypeptide chain, but could damage the integrity of cell membranes and reduce cell viability when treating tissues with a long duration. Considering the properties of different enzymes and the heterogeneous cell types in organs, we have optimized the protocols to achieve better dissociation and higher cell viability for each organ. We observed well clustering of the major cell types independently, which derived from different organs, suggesting that the dissociation procedures had minimal effects on the transcriptional profiles for the majority of cells. For more evidence, please refer to our response to the below comment Point 12 (**Response Pages 48-52**). However, we could not rule out the possibility that the dissociation procedures might have impact on some certain cell types, which await further investigations.

As suggested, we made comments in the **DISCUSSION** section. Please kindly check in Paragraph 2, Page 22. We also provided detailed information for the dissociation procedures and the evaluation of potential effects of dissociation-induced stress on the transcriptome as Supplementary Methods (**Additional file 30**).

6) Authors should provide the cell type labels instead of cluster numbers in Figure 2 D and throughout the manuscript.

**Response:** Thanks for the suggestion. In this revision, we labeled all cell clusters with corresponding cell types, except that there is insufficient space to present.

7) The accuracy of cell type annotations throughout the manuscript is unclear.

**Response:** Thanks for the comment. First, we handled our data and annotated cell types with analytic software and pipeline that have been applied in multiple studies (The Tabula Muris Consortium *et al., Nature* 2018, 562:367-372; Han *et al., Nature* 2020:581, 303-309). Second, we annotated all cell clusters with marker genes for each cell type, which are well-known or have been published previously (please kindly check the gene list and corresponding references in the **Additional file 2: Table S3** in this revision). Specifically, for a subtype of major cell type, we named it “major cell XXX_high” based on the most specifically highly expressed marker gene XXX in the cluster in **Additional file 2: Table S4-S19** in this revision (**Table S3** in the previous version). Third, the paired TCR and BCR information confirmed the accuracy of T cell and B cell annotations, respectively, and the developmental trajectory coupled with TCR/BCR information further confirmed the accuracy of annotation for T/B cell subtypes. Fourth, our trajectory analyses revealed developmental relationship for immune cells, such as monocyte-macrophage, that are consistent with the previous understanding but at a higher single-cell resolution. Fifth, we observed that our findings are consistent with a very recent study in human organ samples (Han *et al., Nature* 2020:581, 303-309), in terms of the gene expression profiles of B cells and epithelial cells and the transcriptional factor activity between both digestive and non-digested tissues. Sixth, we identified multiple TFs for the regulation of cell development between cell types, of which some are well-known. For example, *SPI1* are involved in the development of monocytes to macrophages. Moreover, some TFs for human B cells and plasma cells could be consistently found in a very recent study (Han *et al. Nature* 2020:581, 303-309). Lastly, as suggested by the Reviewer #2, we have also validated our discovery of novel/rare cell types using immunofluorescence staining assays. Taken together, these strongly suggest that the analytic procedures and the cell type annotations in our study are robust.

8) The expression of marker genes should be shown in a heatmap or dot plot in the main figure.

**Response:** Thanks for the suggestion. As suggested, we added t-SNE plots to show the expression of marker genes for major cell types in the main figure (**Fig. 1C**).

9) Automated doublet detection should be compared to manual exclusion of doublets. What do authors refer to as "unassigned" cell types (e.g. "Clusters due to cell doublets or unassigned cell types were identified and excluded manually <..>")?

**Response:** Thanks for the comments. Initially, we had applied DoubletFinder to our data. We observed that only a few cells were inferred as doublets and some proportion of cells were still self-clustered and deviated from other cells, meaning that these are likely doublets overlooked by the automated software. Therefore, we applied a more stringent method, which was based on the similarity of the markers and gene expression profiles of cell types to determine doublets manually. As suggested, we compared two methods for the removal of doublets, namely the automated detection and manual exclusion, using muscle and heart as examples. We found that 15.76% and 10.8% of cells were identified as doublets manually in muscle and heart samples, respectively. By contrast, 5% of cells were inferred as doublets for both samples using the automated DoubletFinder. Moreover, the doublets identified manually cover 47.06% (216 out of 459) and 65.73% (234 out of 356) doublets identified by DoubletFinder in the muscle and heart tissues, respectively. In order to minimize the potential impact of these doublets on the downstream analyses, we have applied the manual method as a more conservative way to remove doublets in our dataset. For more detailed comparison results including figures, please kindly refer to our response to the comment raised by the Reviewer #1 (point 3 in **Response Page 9-13**). In this revision, we have added above descriptions in **Supplementary Methods (Additional file 30).**

We defined doublets as any cell with 1) expression of multiple markers of different cell types, 2) a gene expression profile overlapping with multiple cell types, and 3) more than twice median numbers of UMIs and genes compared to major cell types within each organ, which were removed manually. The “unassigned cells” were those with less confidence to define as doublets, meeting only one or two of the above three criteria. The “unassigned cells” formed clusters in the bladder, lymph node, muscle, skin and the merged data, independently. DoubletFinder could not identify these cells as normal or doublets. Therefore, we applied a conservative way by defining both mixed cells and "unassigned cells” as doublets and removing them from subsequent analyses. In this revision, we have updated the definition in the **METHODS** section. Please kindly check in the last sentence in Paragraph 1, Page 26.

10) The pseudotime analysis of T cells and myeloid cells reflects transcriptional similarities. The authors overinterprete the connection of these trajectories to the developmental stage of cells, provide no new insights or lack of experimental validation or further supporting data. The analysis of gene expression dynamics along pseudotime could add value to this analysis.

**Response:** Thanks for the suggestion. Initially, we had tried to analyze gene expression dynamics along pseudotime using monocle 2. We observed multiple branches and nodes in the trajectory trees for T cells and myeloid cells, however, we did not observe a clear dynamic change of genes related to the development. These might be explained by that monocle 2 could not handle cells with too many subtypes (**Response Figure 10 A-C**). The new version of monocle (monocle 3 alpha and 3) could handle more complicated trajectory analysis with more different developmental states of cells (http://cole-trapnell-lab.github.io/monocle-release/monocle3/). However, too many developmental nodes identified by monocle3 alpha make it hard to resolve gene expression dynamics along pseudotime like in monocle 2 with limited nodes.

Alternatively, we presented the expression of genes that are known to regulate the corresponding cells in the pseudotime plots as in **Fig. 2C, D** and **Additional file 10: Figure S7G-H** in this revision. In general, we observed consistency between the development of cells and the expression of marker genes regulating the developmental process. For examples, *TCF7* and *LEF1* are Wnt pathway effectors and were reportedly expressed at the highest levels in naïve and central memory cells and lower levels in transitional and effector memory cells (Billingsley *et al., PLoS Pathog* 2015, 11:e1004740). We observed high activity and expression of *TCF7* and *LEF1* in CD4^+^, CD8^+^ T_N_, and T_CM_ cell clusters (**Fig. 2D, E**, and **Figure S7G, H)**. *FOXP3* is a specific marker of Treg cells and necessary for their suppressive activity on T cells (Hori *et al., Science* 2003, 299:1057-1061). We observed that *FOXP3* was only enriched in CD4^+^ Treg cluster in the pseudotime plot and had higher activity in this cluster (**Fig. 2D**). Similarly, we observed that *SPI1* had higher activity in monocytes and had higher expression level in corresponding monocyte clusters (**Fig. 4F, G**).

We have updated the results in the main text. Please kindly check in the **RESULTS** section in Paragraph 2, Page 8; **Additional file 10: Figure S7G, H**; Paragraph 2, Page 13 and **Fig. 4G**.

**Response Figure 10. Pseudo-time of CD4^+^, CD8^+^ T cells, and monocytes/macrophages**

**A.** Pseudo-time of CD4^+^ inferred by Monocle2 (left panel). Each point corresponds to a single cell. Clusters information was shown at the right panel. Selected genes expression along the psedutime were shown in the right panel. *TCF7* and *LEF1* for T_CM_ and T_N_, *STAT1* and *IRF1* for T_EFF_, *FOXP3* for T_reg_, and *FOS* for T_RM_.

**B.** Pseudo-time of CD8^+^ inferred by Monocle2 (left panel). Each point corresponds to a single cell. Clusters information was shown at the right panel. Selected genes expression along the psedutime were shown in the right panel. *TCF7* and *LEF1* for T_CM_ and T_N_, *STAT1* and *TBX21* for T_EFF_, and *FOS* and *ATF3* for T_RM_.

**C.** Pseudo-time of monocytes and macrophages, inferred by Monocle2 (left panel). Each point corresponds to a single cell. Clusters information was shown at the right panel. Selected genes expression along the psedutime were shown in the right panel. *RXRN* and *ELF2* for classical monocytes, *TCF7L2* and *STAT1* for non-classical monocytes and LIPA_macrophage, and *JUN* and *PRDM1* for most of non-classical monocyte clusters.

11) The exclusion of cell types from a different lineage could improve the trajectory analysis, for example, gamma delta T cells.

**Response:** Thanks for the suggestion. As suggested, we performed a new trajectory analysis by removing the γδ T cells from CD8^+^ T cells in this revision. We observed consistent trend of development status in the trajectory tree, where T_N_ cells sprout into T_h1_, T_CM_, and T_RM_ branches for CD4^+^ T cells and T_CM_, T_EFF_, IEL, and T_RM_ branches for CD8^+^ T cells (**Fig. 2C**). We updated the result in the main text. Please kindly check the **RESULTS** section in in Lines 2-4, Paragraph 3, Page 7.

12) The transcription factor signature in T cells that comprised FOS, FOSB, JUN, JUND, etc is concerning since these have been previously shown to be upregulated in response to tissue dissociation-induced stress (Brink et al, 2017 Nature Methods). The authors should explain how they can exclude this is not an artifact of the tissue processing.

**Response:** Thanks for the suggestion. We fully understand the Reviewer’s concern about the potential effect of dissociation-induced stress on cells, which should be minimal on overall transcriptome. First, we evaluated the effect of tissue processing on cells in our data, using a method described previously (the Tabula Muris consortium *et al.,* *Nature 2018, 562:367-372*). We observed that most organs had higher density of low total dissociation score, meaning that dissociation-related genes were not significantly induced in the majority of cells (**Response Figure 11**). Second, the typical dissociation-related satellite-cell genes (van den Brink SC *et al., Nat Methods* 2017, 14:935-936), such *FOS*, *HSPA1A* and *SOCS3*, were not consistently expressed in all cell types in muscle (**Response Figure 12**). *HSPA1A* had lower expression level in both two satellite-cell clusters than other clusters. SOCS3 only slightly expressed in one of two satellite-cell clusters. Third, dissociation-related genes showed different expression pattern in a same organ where all cells were treated with a same dissociation procedure (**Response Figure 13**). For example, in the small intestine and stomach, *FOS*, *JUN*, and *JUNB* broadly expressed in different cell clusters with various degree, while *SOCS3* and *HSPA1A* only specifically highly expressed in adoptive cell, macrophage in the small intestine, and enriched in monocyte, fibroblast, and chief cell in the stomach. Fourth, we observed that same cell types from different organs treated with different dissociation procedures had similar expression pattern of the dissociation-related genes. Taking T, B, and plasma cells in the small intestine and stomach for example, each of them had similar expression pattern of those dissociation-related genes. Moreover, each major cell type from different organs was clustered together in t-SNE plot, suggesting similar transcription profiles in spite of different dissociation treatments (**Fig. 1B**).

Taken together, the above observations suggest that dissociation processing in our study might have a minimal impact on the transcriptome of majority of cells in our dataset. Nevertheless, we could not rule out the possibility that the dissociation procedures might have impact on some certain cell types, which await further investigations. Please kindly check at Paragraph 2, Page 22 in the **DISSCUSSION** section. We also provided detailed information for the dissociation procedures and the evaluation of potential effects of dissociation-induced stress on the transcriptome as Supplementary Methods (**Additional file 30**).

**Response Figure 11. Dissociation scores for each organ**. A score represents the magnitude of the first principal component of principal component analysis based on the expression of 140 human dissociation-associated genes of each organ (van den Brink SC *et al., Nat Methods* 2017, 14:935-936). The y axis shows the probability density of the normalized histogram.

**Response Figure 12**. **Violin plot showed the normalized expression of typical dissociation-related genes in the muscle sample.** Shown were *SOCS3*, *FOS,* and *HAPA1A1*.

**Response Figure 13. Violin plot showed the normalized expression of typical dissociation-related genes in the small intestine and stomach samples**. Shown were *FOS*, *FOSB*, *JUN*, *SOCS3*, and *HAPA1A*.

13) The claims related to COVID-19 are unjustified given the lack of biological replicates and the absence of data from key organs involved in the disease (upper and lower airways). Recently, researchers have used single-cell datasets to address this question in a thorough manner (Sungnak et al 2020, https://arxiv.org/abs/2003.06122; Ziegler et al., in revision, <https://papers.ssrn.com/sol3/papers.cfm>? abstract_id=3555145). "Our dataset revealed ACE2 expression in multiple cell types and organs, which overlapped with the suffered organs (lung, heart, trachea, stomach, small intestine, artery and muscle) of HCoV-19 infection[75]."

**Response:** Thanks for the comment. In the outbreak of COVID-19, many scientists have been tried their bests to provide any potential supports to contain the spreading and find clues for better treatments as well as develop effective vaccines. We started this study before the outbreak and were keen to provide potential hints for clinical trials using our data when the complications of COVID-19 especially the acute cardiac injury as frequently reported. Therefore, we thought that it might be important to provide a landscape of the ACE2 expression in human organs, especially heart tissue. As mentioned by the Reviewer, many researchers have reported much thorough results regarding this topic. Considering that our finding related to COVID-19 is yet to be conclusive, we would like to remove this section in this revision.

14) Authors use pseudotime trajectory inference on circulating monocytes and macrophages pooled from all tissues, however, they fail to take into account that most tissue-resident macrophages are not derived from circulating monocytes but from embryonic progenitors (e.g. self-renewing embryonic macrophages in the skin, liver or gut). As mentioned above, the consideration of the biological context is essential for a robust interpretation of trajectory analysis.

**Response:** Thanks for the thoughtful comments. We fully agree with the Reviewer that most tissue-resident macrophages are derived from embryonic progenitors and undergo self-maintenance via local expansion in the steady human organ, with minimal contribution from circulating monocytes (Hashimoto *et al., Immunity* 2013, 38:792-804; Jakubzick *et al., Immunity* 2013, 39:599-610; Sieweke MH *et al., Science* 2013, 342:1242974).

In our study, we performed trajectory analysis to investigate the potential developmental relationship between monocytes and macrophages. The developmental trajectory path from classical monocytes to tissue-resident macrophages suggests that classical monocytes could be a protentional origin of adult tissue-resident macrophages (**Fig. 4E**). One the other hand, high expression of proliferation markers including *MKI67*, *PCNA*, and *TYMS* were exclusively detected in the C12 macrophages (including those in bladder, common bile duct, esophagus, heart, and rectum). Moreover, GSEA analysis also revealed that most of the macrophage clusters had higher enrichment score of G2M checkpoint and E2F targets pathways (**Additional file 14: Figure S10E**). These observations suggest local expansion potential of tissue-resident macrophages. Therefore, our findings suggest tissue-resident macrophages in steady-state organs might have two origins, one from classical monocytes and another from self-expansion. However, with present data, it is hard to tell that self-expansion alone or slightly together with circulating monocytes contributes to the development of tissue-resident macrophages. Further investigations using cell labeling (Chen *et al., Nat Commun* 2018, 9:5065) or samples from donor who received bone marrow transplantation (Thomas *et al., Science* 1976, 192:1016-1018) may help address the question.

In this revision, although our observations suggest potential development relationship between circulating monocytes and tissue-resident macrophages, we acknowledge that “however, further investigations are awaited to address whether self-expansion alone or slightly together with circulating monocytes contributes to the development of tissue-resident macrophages, considering that most tissue-resident macrophages have been demonstrated originating from embryonic progenitor cells (Hashimoto *et al., Immunity* 2013, 38:792-804; Jakubzick *et al., Immunity* 2013, 39:599-610).” Please check in Lines 10-14, paragraph 2, Page 20 in the **DISCUSSION** section.

15) Epithelial cell analysis is focused on biology that has been previously described. The authors could use this dataset to further understand the core-similarities between epithelial cell subsets (e.g. secretory cells) across tissues as well as the key differences.

**Response:** Thanks for the suggestion. As suggested, we performed additional analyses to examine the similarities of epithelial cells across different organs. First, we compared TFs in epithelial cell clusters, which revealed similar activation of TFs in most non-digestive clusters, as well as in digestive clusters (**Fig. 5G**). This was further confirmed using a recent published dataset (**Additional file 19:** **Figure S13B**). Second, pathway analysis revealed that multiple metabolic pathways were enriched in digestive clusters other than non-digestive cluster, suggesting the enhanced metabolic activity of digestive epithelial cells (**Additional file 18:** **Figure S12C, D**). Lastly, we performed ligand-receptor analysis for epithelial cells across different organs, which revealed that epithelial cells had the most frequent intra-cell interactions among all cell types, suggesting that epithelial cells could interact with each other intensively to regulate their biology functions in each organ (**Additional file 24: Figure S17C** and **Additional file 19: Figure S21**).

In this revision, we added the new results in the main text. Please kindly check in in Line 4-6, Paragraph 2, Page 14; Line 8-11, Paragraph 3, Page 14; Line 3-5, Paragraph 1, Page 15; Line 6-11, Paragraph 2, Page 15; Line 5-9, Paragraph 1, Page 16; Line 9-11, Paragraph 1, Page 21; and Line 16-18, Paragraph 2, Page 21.

16) The purpose of the CellphoneDB interaction analysis is unclear.

**Response:** Thanks for pointing out the overlook. CellphoneDB interaction analysis was conducted to explore cell-cell crosstalk of different cell types in various organs based on the repository of ligands, receptors and their interactions, which mediate cell-cell communication critical to coordinating diverse biological processes (Efremova et al., *Nat Protoc*. 2020, 15(4):1484-1506). We observed high frequency of ligation-receptor pairs, suggesting their mediating for intensive communications between cells (**Fig. 6A-C**). We also observed different interacting frequency between different major cell types, suggesting different activity of cell-cell interaction among different cell types (**Fig. 6E**). Moreover, as suggested by the Reviewer’s comment below (point 17), we also showed the ligand-receptor pairs in the context of the specific cell types expressing them, focusing on the major cell types, which revealed potential biological function of the most frequent interacting pairs (**Figure S18-21**). Taken together, these observations revealed the complex and broad intercellular communication networks within tissues.

In this revision, we added a brief description regarding the purpose of the CellphoneDB interaction analysis in the main text, as read “CellphoneDB interaction analysis was conducted to explore cell-cell crosstalk of different cell types in various organs based on the repository of ligands, receptors and their interactions, which mediate cell-cell communication critical to coordinating diverse biological processes (Efremova *et al.,* *Nat Protoc*. 2020, 15(4):1484-1506)”. Please check in Line 2-6, paragraph 2, Page 18.

17) The authors should show ligand-receptors pairs in the context of the specific cell types expressing them. Furthermore, they should indicate how they calculated the frequency of any given interaction and should not interpret the meaning of this metric.

**Response:** Thanks for the suggestions. In this revision, we presented the most frequent ligand-receptors pairs in the context of the specific cell types expressing them as main **Fig. 6**, while the less frequent ones were remained in the supplementary figures (**Figure S18-21** in this revision). We also provided a description on how we calculate the frequency of any given interaction pairs in the main text and figure legends of **Fig. 6C**, as read “The counts of interactions between any two major cell types was the number of the unique significant interacting pairs between them (mean expression > 0 and *P*-value < 0.05)”. In addition, the expression levels of interacting ligand and receptor in cell types 1 and 2, respectively, were indicated as color temperatures in the **Figure S18-21.**

**Second round of review**

**Reviewer 1**

I have carefully read their response, I think the well revised manuscript is suitable for GB, no concern left to be address.

**Reviewer 2**

Major comments:

Regarding response to 1)

Ok, thank you.

Regarding response to 2)

The points mentioned above are based on well-established human cellular biology, e.g. XBP1 in plasma cell differentiation. This reviewer was rather referring to validating novel findings in either newly generated or existing datasets. The incorporation of the Han et al HCL dataset is a valuable addition, however, the fact that this had to be restricted to only 2 cell types is sub-optimal. There are other available datasets of higher quality that could be used for validation of the COCH+ fibroblasts (e.g. heart datasets: Tucker et al. 2020 Circulation; Litvinukova et al. 2020 biorxiv).

Regarding response to 3)

Ok, thank you.

Regarding response to 4)

The immunofluorescence results in S3C and S4A show only one of the two donors and some of the staining are dubious since they look like background signal, e.g. SCGB2A2). In addition, the genes chosen as specific to rare populations instead seem to be expressed across many cells imaged. Did the authors perform appropriate IF controls? Did the authors also report the results of the second donor? Did the authors also detect these novel cell types in the Human Cell Landscape from Han et al 2020 or other datasets? Do the authors have any hypothesis as to which could be the function of the novel fibroblast subtypes?

Regarding response to 5)

Thank you for adding the detailed protocols. They could be summarized in a table as there are high similarities across organs. The table can display the exact conditions for each organ. Indicate the concentration of dispase, not just the volume. Correct "disassociation protocols" for "dissociation" and "HAPA1A" for "HSPA1A".

The assessment of stress-induced transcriptomic effects is valuable and the fact that the identified clusters are not tissue-specific or dissociation-dependent is convincing. Nonetheless, the second and third points raised in pages 9 and 10 of the additional file 10 ("…FOS, HSPA1A and SOCS3, were not consistently expressed in all cell types in muscle…" and "dissociation-related genes showed different expression pattern in a same organ where all cells were treated with a same dissociation procedure") are not conclusive as to whether there is a stress-induced gene expression effect or not and it may be confusing to keep those points in the text.

Regarding response to 6)

Ok, thank you.

Regarding response to 7)

See response below in point 8.

Regarding response to 8)

This comment goes together with the one above (point 7) and the original suggestion was to show markers for each identified population in the form of a heatmap or dot plot. The authors have opted for showing feature plots of a subset of markers. Showing the expression of marker genes for all populations would be a more complete and transparent way to present the data.

Regarding response to 9)

The authors of DoubletFinder recommend users to threshold doublet calling by calculating the mean-variance-normalized bimodality coefficient of pANN distributions produced during pN-pK parameter sweeps of the data. BCMVN can be used to identify the pK that separates singlets and doublets effectively. Have the authors optimized the threshold for this specific dataset? This may be contributing to the low doublet detection by DoubletFinder.

The authors mention that "the doublets identified manually cover 47.06% (216 out of 459) and 65.73% (234 out of 356) doublets identified by DoubletFinder in the muscle and heart tissues". Since there is limited overlap between the two methods, the authors may want to combine the doublets detected by DoubletFinder with the manually detected ones.

Correct "comparation" for "comparison".

Regarding response to 10)

Validating known markers is not the purpose of trajectory analysis. As it stands now, this trajectory analysis does not lead to new biological insights.

The authors should also take into account that gdT are not CD8 T cells, Tcm are CCR7+ and MAIT are thought to develop via a different route.

Could the HSP1A1A CD8+ population be a stress-induced artefact?

Regarding response to 11)

Ok, thank you.

Regarding response to 12)

Response above in point 5.

Regarding response to 13)

Ok, thank you.

Regarding response to 14)

Although authors have softened the language and incorporated the possibility that tissue-macrophages may be derived from embryonic cycling macrophages, the authors could have incorporated some comparisons with published fetal data, such as gut macrophages from Gao et al., 2018 (doi: 10.1038/s41556-018-0105-4). At minimum, the authors could provide novel pathways that change in monocyte-macrophage differentiation trajectory.

Regarding response to 15)

The authors should clarify which tissues do they classify as digestive tissue and what are the implications of this observation.

Regarding response to 16)

Ok, thank you.

Regarding response to 17)

Thanks for the addition of this data.

**Authors Response**

**Point-by-point responses to the reviewers’ comments:**

**Please note that all comments from the Reviewer #1 had been fully addressed and we only keep the comments from the Reviewer #2 yet to be fully addressed, together with the original comment from the Reviewer #2 (in black), our *previous Response(R1)* (in *black* and *Italic*) in the first revision, additional comments from the Reviewer #2 (in underlined and black), and our new response (in Blue), for better following-up.

**Reviewer #2:**

Summary:
He et al have compiled a single-cell RNA-seq and VDJ-seq dataset including 15 different organs from a single individual. The authors describe cell types and patterns of B and T cell clone distributions across organs. While many of the findings are expected, the authors make some interesting observations, however, the lack of replicates and experimental validation weakens any conclusion regarding novel and/or rare cell types.

***Response(R1)****: Thanks for the positive comment. In this revision, we have provided additional analyses and experimental validation for the identification of novel and/or rare cell types, which could be found below as to address the Reviewer’s comments.*

Overall, the authors have addressed many of the issues raised by the reviewers and the manuscript has improved significantly upon revision, however, there are still major concerns that preclude its publication following Genome Biology standards. Language and terminology should be carefully revised throughout the manuscript. See below for further details.

**Response:** Once again, we have been grateful to the Reviewer for thoughtful suggestions that helped improve our manuscript significantly. We fully understand that *Genome Biology* is one of the top journals in research areas of biology and biomedicine and we have been trying our best to present this work to a broader readership so that it would contribute useful information to the scientific community.

In this revision, we have improved further our manuscript with additional analyses and validation experiments according to the Reviewer’s suggestions. Moreover, we have carefully revised the manuscript, with language editing by a native English speaker and terminology confirmation/revision.

Major comments:

2) This study has profiled only one donor. Given the high biological variation in humans, the applicability of these findings to the broader population are under question.

***Response(R1):*** *Thanks for the pertinent comment. We fully understand the Reviewer’s concern if our findings could be applied to broader population in the context of biological variations among individuals. In our study, thousands of cells from multiple organs were analyzed, which enables us to understand the heterogeneous composition of cells in human organs and the development links among cell types, and to identify important molecules and their networking. Such as, similar with the report by The Tabula Muris Consortium et al. (Nature 2018, 562:367-372), we observed close clustering of cells from different organs (more than seven organs) for major cell types, including T cells, B cells, NK cells, fibroblasts, endothelial cells, smooth muscle cells, macrophages, and monocytes. We also identified several well characterized TFs regulating the different cell development, including TCF7, SELE, MYC, and KLF2 for T_N_ cells, TBX21, STAT1, and IRF1 for T_EFF_ cells, and PRDM1, XBP1, FOS, and IRF4 for plasma cells. The consistencies between our findings and previous findings suggest that our study based on large scale transcriptomes at single cell level provides common biological understanding at higher resolution.*

*Moreover, we used an independent set of single cell sequencing data derived from human organs, which was published very recently (Han et al. Nature 2020:581, 303-309), and we managed to replicate our findings. First, we identified important transcription factors regulating plasma cell development, such as XBP1, MYC, etc., which have similar activation patterns in both datasets (Fig. 3E and Figure S9F). Second, epithelial cells from different organs showed strong heterogeneity, while epithelial cells within the same tissue had similar gene expression profile and clustered together, in both datasets (Fig. 5A and Figure S11B). Third, we found that both digestive and non-digested tissues showed similar transcriptional factor activities in both datasets (Fig. 5G and Additional file 19: Figure S13B). In addition, we note that we only selected B cells and epithelial cells for replication analyses, because the recent published data has low sequencing depth and extensive contamination between different cell types (Han et al., Nature 2020:581, 303-309).*

*Furthermore, we validated our discovery of novel and/rare cell types using experiment as suggested by the Reviewer #2, including sweat gland epithelial cell in skin, COCH^+^ fibroblast in the skin and heart, and FibSmo in the bladder, heart, and rectum in independent samples (Figure S3C and Figure S4A). Please check more details regarding this at below Reviewer’s comment point 4 (Response(R1) Page 38).*

*Taken together, although our data was derived from one donor, some important findings based on large number of cells and multiple organs might be generally applicable. However, we do acknowledge that further investigations with more individual samples will solidify our findings and discover more novel cell types, key molecules, and interaction network.*

*We have added the replication analyses and experimental results as well as limitation in this revision. Please kindly check in the RESULTS section in Paragraph 2, Page 6; Paragraph 1, Page 11; Line 4-7, Paragraph 2, Page 14; Line 8-13 Paragraph 3, Page 14; Line 3-6, Paragraph 1, Page 15; Line 6-12, Paragraph 2, Page 15; and Line 5-9, Paragraph 1, Page 16.*

The points mentioned above are based on well-established human cellular biology, e.g. XBP1 in plasma cell differentiation. This reviewer was rather referring to validating novel findings in either newly generated or existing datasets. The incorporation of the Han et al HCL dataset is a valuable addition, however, the fact that this had to be restricted to only 2 cell types is sub-optimal. There are other available datasets of higher quality that could be used for validation of the COCH+ fibroblasts (e.g. heart datasets: Tucker et al. 2020 Circulation; Litvinukova et al. 2020 biorxiv).

Please, indicate what does FibSmo stand for.

**Response:** Thanks for the suggestions. As suggested, we performed additional analyses using several existing public datasets and multiplex immunofluorescence staining in more human tissue samples to validate our novel findings.

First, we validated the presence of *COCH*^+^ fibroblasts in multiple skin and heart tissues (**Response Figure 1**), using two skin datasets (Solé-Boldo *et al., Communications Biology* 2020, 3:188 and He *et al., Journal of Allergy and Clinical Immunology* 2020, 145:1615-1628 and) and one heart dataset (Tucker *et at., Circulation* 2020) that are publicly available. Please note that we were not able to access the suggested dataset from Litvinukova *et al*. We observed a clear clustering of *COCH*^+^ fibroblasts with co-expression of *MMP2* and *COCH* genes in the skin dataset of Solé-Boldo *et al.*’s (cluster 30; **Response Figure 1A:** left top and **B:** left). A total of 144 *COCH*^+^ fibroblasts (out of 7,298 fibroblasts and 7,879 other cells in the skin) were identified in 5 skin samples in the dataset, with maximum 103 cells for one sample. We also observed a small group of *COCH*^+^ fibroblasts with high expression of both *COCH* and *MMP2* in the skin dataset of He *et al.*’s (cluster 18; **Response Figure 1A:** right and **B:** middle). A total of 125 *COCH*^+^ fibroblasts (out of 5,657 fibroblasts and 19,669 other cells in the skin) were identified in 14 skin samples in the dataset, with maximum 31 cells for one sample. By contrast, 454 *COCH*^+^ fibroblasts cells scattered among a total of 62,292 fibroblasts from the 26 heart samples in Tucker *et al*.’s dataset, with maximum 45 cells for one sample (clusters 1, 2, 4, 6, 7, 10; **Response Figure 1A:** left bottom and **B:** right). Noteworthily, we further validated the presence of *COCH*^+^ fibroblasts in more human tissue samples using multiplex immunofluorescence staining assays (Please kindly refer to Reviewer #2’s comment 4 below).

**Response Figure 1.** Expression of *COCH* and *MMP2* in cells from the skin and heart samples.

**A**. Violin plots showed the normalized expression of *COCH* and *MMP2* in cells of different clusters from the skin (top) and heart (left bottom) tissues retrieved from public datasets as indicated. Clusters were numbered at x-axis, and the expression level was shown in y-axis. Each dot represents a single cell.

**B**. Feature plots showed the normalized expression of *COCH* and *MMP2* in cells from skin (left and middle) and heart (right) tissues retrieved from public datasets as indicated at the bottom.

Next, we observed similar gene signatures of *COCH^+^* fibroblasts between our AHCA dataset and Solé-Boldo *et al.*’s dataset, compared to all other cells in respective dataset (**Response Figure 2**). 31 out of 50 upregulated genes in *COCH^+^* fibroblasts were shared between the two datasets, among which *COCH* and *ASPN* are the two most significant ones (**Response Figure 2**). Both *COCH* and *ASPN* are secretory proteins in the extracellular matrix and have been involved in extracellular matrix remolding (Cho *et al., J Mol Med (Berl)* 2012, 90:1321-1331; Goel *et al., PLoS One* 2011, 6:e23070; Ikegawa, *Curr Med Chem* 2008, 15:724-728), suggesting the important role of *COCH^+^* fibroblasts in maintaining tissue homeostasis.

Second, we validated the presence of *SCGB2A2*^+^ sweat gland cells in multiple skin samples from the above two datasets (**Response Figure 3**). We observed a clear clustering of *SCGB2A2*^+^ sweat gland cells with co-expression of *DCD*, *SCGB2A2*, *PIP*, *KRT19*, and *MUCL1* genes in the skin dataset of He *et al.*’s (cluster 25; **Response Figure 3A** and **B**: left). A total of 346 *SCGB2A2*^+^ sweat gland cells (out of 25,326 cells in the skin) were identified in 14 skin samples in the dataset, ranging from 5 to 102 cells for each individual sample. By contrast, we only observed one *SCGB2A2*^+^ sweat gland cell with co-expression of *DCD*, *SCGB2A2*, *PIP*, *KRT19*, and *MUCL1* genes in Solé-Boldo *et al*.’s dataset (cluster 28; **Response Figure 3A** and **B:** right panel), as limited epithelial cells (ranging from 99 to 969) were collected from each donor in the study.

**Response Figure 2.** Gene signature of *COCH*^+^ fibroblasts in skin.

**A, B**. Scatter plots showed the differentially expressed genes compared to other cells in skin samples from our AHCA or Solé-Boldo *et al*.’s datasets. Selected genes were labeled if ln(fold-change) greater than 4 and 3 for upregulation (in red), or smaller than 0.25 and 0.33 for downregulation (blue) in the AHCA and Solé-Boldo *et al*.’s datasets, respectively.

**C.** Venn diagram showed the sharing of upregulated genes in *COCH^+^* fibroblasts in comparison to all other cells.

**Response Figure 3.** Expression of *DCD*, *SCGB2A2*, *PIP*, *KRT19* and *MUCL1* genes in cells from skin samples.

**A**. Violin plots showed the normalized expression of *DCD*, *SCGB2A2*, *PIP*, *KRT19*, and *MUCL1* in cells of different clusters from the skin tissues retrieved from public datasets as indicated. Clusters were numbered at x-axis, and the expression level was shown in y-axis. Each dot represents a single cell. The clusters for *SCGB2A2*^+^ sweat gland cells were boxed.

**B**. Feature plots showed the normalized expression of *DCD*, *SCGB2A2*, *PIP*, *KRT19*, and *MUCL1* in cells from skin tissues retrieved from public datasets as indicated at the bottom.

Third, we validated the presence of *MMP2*^+^*ACTA2*^+^ cells (assigned as FibSmo cells) in multiple bladder samples from two independent public datasets (Yu *et al., J Am Soc Nephrol* 2019, 30:2159-2176; Han *et al., Nature* 2020, 581:303-309; **Response Figure 4**). We observed a clear clustering of FibSmo cells with co-expression of *MMP2* and *ACTA2* genes in both datasets. A total of 1,447 FibSmo cells (out of 13,490 cells in the bladder) were identified in 3 bladder samples in the Yu *et al*.’s dataset, ranging from 42 to 709 cells for each individual sample (**Response Figure 4A** and **B:** left). Similarly, a total of 178 FibSmo (out of 1,193 stromal cells in the bladder) were identified in 2 bladder samples in the Han *et al.*’s dataset, with 24 and 154 cells in each sample (**Response Figure 4A** and **B:** right). Moreover, we confirmed the presence of FibSmo cells in multiple human samples using immunofluorescence staining assays (Please kindly refer to the Reviewer’s comment 4 below).

In summary, we confirmed the presence of *COCH*^+^ fibroblasts, *SCGB2A2*^+^ sweat gland cells, and FibSmo cells in multiple human tissue samples using additional existing datasets and immunofluorescence staining assays.

We named FibSmo cells as a novel cell type with co-expression of *MMP2* and *ACTA2*, which are marker genes for fibroblast and smooth muscle cells, respectively. Although FibSmo cells share marker genes with the two cell types, they had distinct gene signatures (**Response Figure 5A** and **B**). FibSmo cells exhibit high and specific expression of *PLAT* and *ID1,* which were related to clotting and angiogenesis (Carmeliet *et al., Nature* 1994*,* 368:419-424; Volpert *et al., Cancer Cell* 2002, 2:473-483), and higher expression of *COL3A1* and *COL1A1* compared to other stromal cells, which have been involved in wound healing (Crane *et al., Wound Repair Regen* 2010, 18:409-416). In addition, the enrichment of BMP signal genes *BMP4* and *BMP5* was also observed in the FibSmo cells (**Response Figure 5B**). GO analysis revealed that FibSmo cells shared common stromal functions with fibroblasts and smooth muscle cells, such as *extracellular structure organization* and *tissue remolding*, while FibSmo cells had enhanced biological functions in *response to wounding* and *response to growth factor*, which is consistent with the functions of their highly expressed signature genes above (**Response Figure 5C**).

In this revision, we have added the above validation results and a more detailed description of FibSmo cells in the main text and the Appendix. We also updated the related Methods. Please kindly check at locations (to be updated).

**Response Figure 4**. Expression of *MMP2 and ACTA2* genes in cells from bladder samples.

**A**. Violin plots showed the normalized expression of *MMP2 and ACTA2* in cells of different clusters from the bladder tissues retrieved from public datasets as indicated. Clusters were numbered at x-axis, and the expression level was shown in y-axis. Each dot represents a single cell. The clusters for FibSmo cells were boxed.

**B**. Feature plots showed the normalized expression of *MMP2 and ACTA* in cells from skin tissues retrieved from public datasets as indicated at the bottom.

**Response Figure 5.** Gene expression and estimated functions of fibroblast, FibSmo, and smooth muscle cells.

**A**. Heatmap showed the average expression level of top 150 genes enriched in fibroblast, FibSmo, and smooth muscle cells as indicated in rows. Each column line represents one gene and the expression level from high to low was coded as red to blue, respectively.

**B**. Violin plots showed the expression of marker genes of FibSmo in the three cell types as indicated at the bottom.

**C**. Bar plots showed the selected biological functions enriched in fibroblast, FibSmo, and smooth muscle cells. Key genes were listed for enhanced GO term of different cell types.

4) The identification of rare/novel cell types (e.g. Langerhans cells and COCH+ fibroblast subtypes) should be supported by replicates/validation.

***Response(R1):*** *Thanks for the comment. To address the comment, we performed immunofluorescence staining assays to confirm the presence of rare/novel cell types identified in our study. We managed to locate sweat gland epithelial cell in two independent skin samples, which were stained with makers KRT19, SCGB2A2, and PIP (Additional file 5: Figure S3C). Similarly, we validated the novel fibroblast subtype by staining markers COCH and MMP2 in the skin and heart samples from two independent individual donors (Figure S3C). Importantly, we confirmed the novel cell type FibSmo with markers MMP2 and ACTA2 in the bladder, heart, and rectum samples from two independent individual donors (Figure S4A). Please note that we don’t examine the presence of Langerhans cell in tissue samples, because this cell type has been widely reported (Merad et al., Nat Rev Immunol 2008, 8:935-947; Clayton et al., Front Immunol 2017, 8:1676).*

*We have added the above results in this revision. Please kindly check in Line 15-18, Paragraph 2, Page 6 in the RESULTS section.*

The immunofluorescence results in S3C and S4A show only one of the two donors and some of the stainings are dubious since they look like background signal, e.g. SCGB2A2). In addition, the genes chosen as specific to rare populations instead seem to be expressed across many cells imaged.

Did the authors perform appropriate IF controls? Did the authors also report the results of the second donor? Did the authors also detect these novel cell types in the Human Cell Landscape from Han et al 2020 or other datasets? Do the authors have any hypothesis as to which could be the function of the novel fibroblast subtypes?

**Response:** Thanks for the suggestions. Yes, we previously provided representative immunofluorescence (IF) images for only one donor in the previous figure S3C and S4A, although the IF assay was done in samples from two donors. To address the Reviewer’s comments, we have performed IF assays with more samples. All the results from multiple donors will be provided as supplementary Figures.

For sweat gland epithelial cell, we repeated multiplex IF assays in the two donors (donor 1 and 2) as in our previous manuscript. Again, co-localization of SCGB2A2, KRT19, and PIP proteins were identified for the *SCGB2A2*^+^ sweat gland epithelial cells (**Response Figure 6**). We note that the expression of SCGB2A2 is profound in skin cells but is limited to a certain cell type. This is consistent with the observation in another skin sample (donor 3), which was stained with only SCGB2A2 as a positive control (**Response Figure 6**).

For FibSmo cells, we performed multiplex IF assays in additional samples and presented the representative IF images for all samples. We observed co-localization of MMP2 and ACTA2 proteins in numbers of FibSmo cells in bladder (4 donors), heart (2 donors), and rectum samples (2 donors; **Response Figure 7**). FibSmo shared common stromal functions with fibroblasts and smooth muscle cells, such as *extracellular structure organization* and *tissue remolding*, while FibSmo cells had enhanced biological functions in *response to wounding* and *response to growth factor*, which is consistent with the functions of their highly expressed signature genes (**Response Figure 5C**). For more information regarding the gene expression signatures and functions, please kindly refer to the Reviewer’s comment 2 above.

For *COCH*^+^ fibroblasts, we also repeated the multiplex IF analysis in the previous two donors, which revealed co-localization of COCH and MMP2 staining in the 4 skin and heart samples (**Response Figure 8**).

In summary, the multiplex IF assays in additional samples confirmed the presence of *SCGB2A2*^+^ sweat gland epithelial cells, FibSmo cells, and *COCH*^+^ fibroblasts in human. Consistently, the presence of such cell types was also validated in human samples with publicly available single cell sequencing data. Please kindly refer to our response to the Reviewer’s point 2 above. We note that the identifications of these cell types were based on co-expression of multiple cell markers, which is not specifically expressed for a certain cell type. Therefore, it is plausible that we might observe a broad expression of one of these markers across different cell types. We acknowledge that further investigations could be done to identify more specific markers for these cell types.

**Response Figure 6**. Representative images of immunofluorescence staining in skin samples for sweat gland epithelial cells. Proteins used in the assays are indicated at the left, and the samples from different donors (1-3) were listed as columns. The red arrows indicated the co-expression of SCGB2A2, PIP, and KRT19 proteins in cells in donor 1 and 2. Donor 3 was severed as positive control of SCGB2A2 staining. The red arrows indicated the specifically expression of SCGB2A2 in certain cell types in the skin in donor 3.

**Response Figure 7**. Representative images of immunofluorescence staining for FibSmo cells in bladder, rectum, and heart tissues. Proteins used in the assays are indicated on top, and the bladder, heart, and rectum samples from different donors (1-4) were listed as rows. The red arrows indicated the co-expression of ACTA2 and MMP2 proteins in tissue cells.

**Response Figure 8**. Representative images of immunofluorescence staining for *COCH*^+^ fibroblast cells in skin and heart tissues. Proteins used in the assays are indicated on top, and the skin and heart samples from different donors (1-2) were listed as rows. The red arrows indicated the co-expression of COCH and MMP2 proteins in cells of samples as indicated at the left.

5) The authors should provide a complete description of the tissue dissociation method. In addition, they should comment on how the use of different dissociation protocols could result in selective release of cells in different organs and/or induction of stress response.

***Response(R1):*** *Thanks for the suggestions. As suggested, we now provide a detailed description of tissue dissociation protocols in Additional file 30, which includes the procedures as well as the use of enzymes and treatment durations for each organ. We note that these disassociation protocols were established based on the previous publications and rounds of in-house tests on different pig and human tissues until we could achieve high cell viability, less digestive time and less residual tissue.*

*As pointed out by the Reviewer, cellular dissociation was a prerequisite technical manipulation for single cell studies, although dissociation procedures would cause disturbance of transcriptome at various degrees (Brink et al., Nat Methods 2017, 14:935-936). Enzymes and duration of dissociation procedures might be two important factors (Harris et al., Hippocampus 2019, 29:876-882; Brink et al., Nat Methods 2017, 14:935-936). A gentle enzyme and short time might help to minimize the disturbance of transcriptome or induction of stress response. Moreover, an organ consists different cell types and extra cellar matrix, which need different enzymes to dissociate them into single cells. It’s unlikely that one enzyme could fit for all organs or cell types. Long duration and high concentration of enzymes could have a higher degree of dissociation. However, it comes with higher level of damage on cells and disturbance of transcription. Collagenase could only degrade the triple-helical native collagen fibrils; Dispase cleaving fibronectin, type IV collagen and type V collagen are gentle and have limited damage to cells; and Trypsin could cleave polypeptide chain, but could damage the integrity of cell membranes and reduce cell viability when treating tissues with a long duration. Considering the properties of different enzymes and the heterogeneous cell types in organs, we have optimized the protocols to achieve better dissociation and higher cell viability for each organ. We observed well clustering of the major cell types independently, which derived from different organs, suggesting that the dissociation procedures had minimal effects on the transcriptional profiles for the majority of cells. For more evidence, please refer to our response to the below comment Point 12 (Response(R1) Pages 48-52). However, we could not rule out the possibility that the dissociation procedures might have impact on some certain cell types, which await further investigations.*

*As suggested, we made comments in the DISCUSSION section. Please kindly check in Paragraph 2, Page 22. We also provided detailed information for the dissociation procedures and the evaluation of potential effects of dissociation-induced stress on the transcriptome as Supplementary Methods (Additional file 30).*

Thank you for adding the detailed protocols. They could be summarized in a table as there are high similarities across organs. The table can display the exact conditions for each organ. Indicate the concentration of dispase, not just the volume. Correct “disassociation protocols” for “dissociation” and “HAPA1A” for “HSPA1A”.

**Response:** Thanks for the suggestions and pointing out the typos. As suggested, we provided a supplementary table to summarize dissociation protocols for each organ, including primary dissociation reagents, addition of CaCl_2_, dissociation buffer, incubation condition and extra dissociation. Please kindly check at Supplementary Table (numbering and location to be updated), which was reproduced as below. In addition, we have corrected the typos in this revision.

**Response Table 1**. The digestion protocols for each organ.

| **Tissue** | **Primary dissociation reagents** | **Addition of CaCl_2_** | **Dissociation buffer** | **Incubation condition** | **Extra dissociation (optional)** |
| --- | --- | --- | --- | --- | --- |
| **Blood** | 0.8% NH_4_Cl red blood cells lysis buffer | NO | RBC buffer | 10 min on ice | NO |
| **Bone marrow** |  |  |  |  |  |
| **Bladder** | 125 U Dispase/5 ml + 1 mg/ml Collagenase IV + 1 mg/ml Collagenase II + 50 ug/ml DNase I | 5 mM | DMEM + 10%FBS | 1 h + 10 min | 0.25% trypsin + EDTA 20 min for residue |
| **Common bile duct** |  |  |  | 40 min + 10 min |  |
| **Esophagus** |  |  |  | 1 h + 10 min |  |
| **Heart** | 50 U Dispase/5 ml + 1 mg/ml Collagenase IV + 1 mg/ml Collagenase II + 50 ug/ml DNase I | 5 mM | DMEM + 10%FBS | 40 min + 10 min |  |
| **Lymph node** |  |  |  |  |  |
| **Muscle (leg)** |  |  |  |  |  |
| **Spleen** | 0.5 mg/ml Collagenase IV + 0.5 mg/ml Collagenase II + 50 ug/ml DNase I | 5 mM | DMEM + 10%FBS | 30 min + 10 min |  |
| **Small intestine** | 0.05% Trypsin + EDTA+ 0.5 mg/ml Collagenase IV + 0.5 mg/ml Collagenase II + 50 ug/ml DNase I | 5 mM added after 20 min | DMEM | 1 h + 10 min |  |
| **Rectum** | 0.05% Trypsin + EDTA+ 1 mg/ml Collagenase IV + 1 mg/ml Collagenase II + 50 ug/ml DNase I | 5 mM added after 20 min | DMEM | 1 h + 10 min |  |
| **Stomach** |  |  |  |  |  |
| **Liver** | Step 1: 1 mM EGTA + 1xDPBS, Step 2: 1 mg/ml Collagenase II + 1 mg/ml Collagenase IV + 50 ug/ml DNase I | 5 mM added after 10 min | Step 1: 1x DPBS, Step 2: DMEM + 10%FBS | Step 1: 10 min, Step 2: 30 min + 10 min |  |
| **Skin** | Step 1: 0.25% Trypsin + EDTA, Step 2: 125 U Dispase/5 ml + 1 mg/ml Collagenase I + 1 mg/ml Collagenase V + 50 ug/ml DNase I | 5 mM added after 20 min | DMEM | Step 1: 20 min, Step 2: 1 h + 10 min |  |
| **Trachea** | Step1: 0.25% Trypsin + EDTA, Step 2: 125 U Dispase/5 ml + 1 mg/ml Collagenase IV + 1 mg/ml Collagenase II + 50 ug/ml DNase I | 5 mM added after 20 min | DMEM | Step 1: 20 min, Step 2: 1 h + 10 min |  |

The assessment of stress-induced transcriptomic effects is valuable and the fact that the identified clusters are not tissue-specific or dissociation-dependent is convincing. Nonetheless, the second and third points raised in pages 9 and 10 of the additional file 10 (“…FOS, HSPA1A and SOCS3, were not consistently expressed in all cell types in muscle…” and “dissociation-related genes showed different expression pattern in a same organ where all cells were treated with a same dissociation procedure”) are not conclusive as to whether there is a stress-induced gene expression effect or not and it may be confusing to keep those points in the text.

**Response:** Thanks for the suggestion. In this revision, we have removed those points from the text.

7) The accuracy of cell type annotations throughout the manuscript is unclear.

***Response(R1):*** *Thanks for the comment. First, we handled our data and annotated cell* *types with analytic software and pipeline that have been applied in multiple studies (The Tabula Muris Consortium et al., Nature 2018, 562:367-372; Han et al., Nature 2020:581, 303-309). Second, we annotated all cell clusters with marker genes for each cell type, which are well-known or have been published previously (please kindly check the gene list and corresponding references in the Additional file 2: Table S3 in this revision). Specifically, for a subtype of major cell type, we named it “major cell XXX_high” based on the most specifically highly expressed marker gene XXX in the cluster in Additional file 2: Table S4-S19 in this revision (Table S3 in the previous version). Third, the paired TCR and BCR information confirmed the accuracy of T cell and B cell annotations, respectively, and the developmental trajectory coupled with TCR/BCR information further confirmed the accuracy of annotation for T/B cell subtypes. Fourth, our trajectory analyses revealed developmental relationship for immune cells, such as monocyte-macrophage, that are consistent with the previous understanding but at a higher single-cell resolution. Fifth, we observed that our findings are consistent with a very recent study in human organ samples (Han et al., Nature 2020:581, 303-309), in terms of the gene expression profiles of B cells and epithelial cells and the transcriptional factor activity between both digestive and non-digested tissues. Sixth, we identified multiple TFs for the regulation of cell development between cell types, of which some are well-known. For example, SPI1 are involved in the development of monocytes to macrophages. Moreover, some TFs for human B cells and plasma cells could be consistently found in a very recent study (Han et al. Nature 2020:581, 303-309). Lastly, as suggested by the Reviewer #2, we have also validated our discovery of novel/rare cell types using immunofluorescence staining assays. Taken together, these strongly suggest that the analytic procedures and the cell type annotations in our study are robust.*

See response below in point 8.

**Response:** Thanks.

8) The expression of marker genes should be shown in a heatmap or dot plot in the main figure.

***Response(R1):*** *Thanks for the suggestion. As suggested, we added t-SNE plots to show the expression of marker genes for major cell types in the main figure (Fig. 1C).*

This comment goes together with the one above (point 7) and the original suggestion was to show markers for each identified population in the form of a heatmap or dot plot. The authors have opted for showing feature plots of a subset of markers for some annotations, however, for other annotations it remains unclear which markers are they based on, e.g. which marker were used to distinguish central memory from naïve T cells or effectors from resident memory? Showing the expression of canonical marker genes for known populations in a transparent way is required. Showing the expression of marker genes for all populations would be a more complete and transparent way to present the data.

**Response:** Thanks for the suggestions. As suggested, we have provided marker genes and their expression for each cell types in this revision, with dot plots or violin plots. Please kindly check at locations (to be updated). For example, in the manuscript **Figure 1** (**B** and **C**), reproduced as below **Response Figure 9**, we presented a dot plot of gene expression for a subset of markers for major cell types (**Response Figure 9B**).

We also added violin plots to show the canonical markers of CD4^+^ and CD8^+^ cells (Zhang *et al., Nature* 2018, 564:268-272; **Response Figures 10** and **11**). We note that *CD4* gene was only significantly detectable in several CD4^+^ T cell clusters, which might be due to the low presentation of 10X Genomics technology (**Response Figure 10B**), while *CD8A* and *CD8B* were broadly expressed in all CD8^+^ T cell clusters (**Response Figure 11B**). We were not able to distinguish TN from TCM using CD45RA^+^CD45RO^-^CCR7^+^ and CD45RA^-^CD45RO^+^CCR7^+^ for the two cell types, respectively (Schmueck-Henneresse *et al., J Immunol* 2015,194(11):5559-67), since our data generated using 10X Genomics is not feasible for the determination of different *CD45* isoforms. Therefore, in our study, we used *CCR7*, *SELL*, *TCF7*, and *LEF1* as markers for TN (naive T cell) and TCM (central memory T cell) according to previous literatures (Hashimoto *et al*., *Proc Natl Acad Sci USA* 2019, 116:24242-24251). Cell clusters with high expression of both *CCR7* and *SELL* (lnFC greater than 0.7 for CD4^+^ and 1.0 for CD8^+^) and top 10 ranking of *TCF7* and *LEF1* were assigned as TN cells*.* Otherwise, clusters with expression of *CCR7* and *SELL* were assigned as TCM cells.

**Response Figure 9**. Cells distribution across different organs and the marker genes of each cluster.

**A**. t-SNE showed the distribution of cells from different tissues coloured according to their origins of organ. Each dot represents a cell. The labeled cell types were the predominant cell types in each cluster.

**B**. Dot plots showed the most highly expressed marker genes (x axis) of major cell types (y axis) in **A**. The depth of colours from white to blue and the size of dot represent the average expression from low to high and the percentages of cells expressing the gene, respectively.

We observed different expression of marker genes between effector T cells (TEFF) and resident memory T cells (TRM; **Response Figure 11**). *FGFBP2* and *TXB21* were common markers for CD4^+^ and CD8^+^ TEFF cells*,* with additional specific markers *GNLY* and *FCGR3A* for CD4^+^ and CD8^+^ TEFF cells, respectively; *MYADM*, *NR4A1*, *CXCR6*, and *ITGAE* were markers for CD4^+^ TRM cells; and *ITGAE*, *XCL1*, and *XCL2* were markers for CD8*^+^* TRM cells (**Response Figure 11**). Moreover, TEFF from TRM cells also showed distinct expression signatures (**Response Figure 12**). Only TEFF cells (Th1_NKG7, TEFF_TRBV4-2, TEFF_MT1E, and TEFF_GNLY) exhibited simultaneously strong expression of effector or cytotoxic genes including *GZMA*, *GZMB*, *PRF1*, *GZMH*, and *NKG7* (Zhang *et al., Nature* 2018, 564:268-272; **Response Figure 12A** and **B**). In addition, we didn’t use *CD69* in combination of *ITGAE* to classify TRM, because *CD69* was detected widely in both CD4^+^ and CD8^+^ T clusters.

In this revision, we have also added the above results in the manuscript. Please check at locations (to be updated).

 

**Response Figure 10**. Cell clusters and expression of canonical markers for each CD4^+^ T cells.

**A**. t-SNE plot of 7,006 CD4^+^ T from 15 organ tissues. Each point represents a cell, coloured according to their origins of organ (top panel) or cluster (bottom panel) as indicated on the right legend.

**B**. Violin plots showed the normalized expression of marker genes for each CD4^+^ T cell cluster as indicated at the bottom. For each panel, y-axis shows the normalized expression level for a marker gene as indicated at the left. Marker genes were also grouped according to functional cell types.

**Response Figure 11**. Cell clusters and expression of canonical markers for each CD8^+^ T cells.

**A**. t-SNE plot of 11,286 CD8^+^ T from 15 organ tissues. Each point represented a cell, coloured according to their origins of organ (top panel) and cluster (bottom panel) as indicated on the right legend.

**B**. Violin plots showed the normalized expression of marker genes for each CD8^+^ T cell cluster as indicated at the bottom. For each panel, y-axis shows the normalized expression level for a marker gene as indicated at the left. Marker genes were also grouped according to functional cell types.

**Response Figure 12**. Normalized expression of effector molecules in CD4^+^ and CD8^+^ T cell clusters.

**A. B**. Violin plots showed the normalized expression of effector molecules for each CD4^+^ and CD8^+^ T cell cluster as indicated at the bottom. For each panel, y-axis shows the normalized expression level for an effector molecule as indicated at the left.

9) Automated doublet detection should be compared to manual exclusion of doublets. What do authors refer to as "unassigned" cell types (e.g. "Clusters due to cell doublets or unassigned cell types were identified and excluded manually <..>")?

***Response(R1):*** *Thanks for the comments. Initially, we had applied DoubletFinder to our data. We observed that only a few cells were inferred as doublets and some proportion of cells were still self-clustered and deviated from other cells, meaning that these are likely doublets overlooked by the automated software. Therefore, we applied a more stringent method, which was based on the similarity of the markers and gene expression profiles of cell types to determine doublets manually. As suggested, we compared two methods for the removal of doublets, namely the automated detection and manual exclusion, using muscle and heart as examples. We found that 15.76% and 10.8% of cells were identified as doublets manually in muscle and heart samples, respectively. By contrast, 5% of cells were inferred as doublets for both samples using the automated DoubletFinder. Moreover, the doublets identified manually cover 47.06% (216 out of 459) and 65.73% (234 out of 356) doublets identified by DoubletFinder in the muscle and heart tissues, respectively. In order to minimize the potential impact of these doublets on the downstream analyses, we have applied the manual method as a more conservative way to remove doublets in our dataset. For more detailed comparison results including figures, please kindly refer to our response to the comment raised by the Reviewer #1 (point 3 in Response(R1) Page 9-13). In this revision, we have added above descriptions in Supplementary Methods (Additional file 30).*

*We defined doublets as any cell with 1) expression of multiple markers of different cell types, 2) a gene expression profile overlapping with multiple cell types, and 3) more than twice median numbers of UMIs and genes compared to major cell types within each organ, which were removed manually. The “unassigned cells” were those with less confidence to define as doublets, meeting only one or two of the above three criteria. The “unassigned cells” formed clusters in the bladder, lymph node, muscle, skin and the merged data, independently. DoubletFinder could not identify these cells as normal or doublets. Therefore, we applied a conservative way by defining both mixed cells and "unassigned cells” as doublets and removing them from subsequent analyses. In this revision, we have updated the definition in the METHODS section. Please kindly check in the last sentence in Paragraph 1, Page 26.*

The authors of DoubletFinder recommend users to threshold doublet calling by calculating the mean-variance-normalized bimodality coefficient of pANN distributions produced during pN-pK parameter sweeps of the data. BCMVN can be used to identify the pK that separates singlets and doublets effectively. Have the authors optimized the threshold for this specific dataset? This may be contributing to the low doublet detection by DoubletFinder.

The authors mention that “the doublets identified manually cover 47.06% (216 out of 459) and 65.73% (234 out of 356) doublets identified by DoubletFinder in the muscle and heart tissues”. Since there is limited overlap between the two methods, the authors may want to combine the doublets detected by DoubletFinder with the manually detected ones. Correct “comparation” for “comparison”.

**Response:** Thanks for the suggestions. As suggested, we combined both “DoubletFinder” and then manual detection methods to identify and remove the doublets in each organ. For “DoubletFinder”, we set an estimated proportion of doublets as 5% and an optimized pK using BCMVN for each organ (**Response Table 2**). We then manually removed the potential doublets clusters in each organ as we mentioned in the previous submission. After that, we rerun all the downstream analyses and updated the results, related figures and tables in this revision. Compared to the previous submission, additional 4,275 ‘cells’ were determined as doublets and removed from the analyses, resulting in a total of 84,363 cells. We note that the removal of new doublets had minimal impact on our findings. In addition, we corrected the typo ‘comparation’ to ‘comparison’. We sincerely apologized for this overlook.

**Response Table 2**. Optimal pK values for each organ.

| **Tissue** | **Optimal pK** |
| --- | --- |
| Bladder | 0.005 |
| Blood | 0.02 |
| Common bile duct | 0.29 |
| Esophagus | 0.005 |
| Heart | 0.005 |
| Liver | 0.005 |
| Lymph node | 0.005 |
| Marrow | 0.02 |
| Muscle | 0.01 |
| Rectum | 0.005 |
| Skin | 0.28 |
| Small intestine | 0.005 |
| Spleen | 0.01 |
| Stomach | 0.01 |
| Trachea | 0.005 |

10) The pseudotime analysis of T cells and myeloid cells reflects transcriptional similarities. The authors overinterpret the connection of these trajectories to the developmental stage of cells, provide no new insights or lack of experimental validation or further supporting data. The analysis of gene expression dynamics along pseudotime could add value to this analysis.

***Response(R1):*** *Thanks for the suggestion. Initially, we had tried to analyze gene expression dynamics along pseudotime using monocle 2. We observed multiple branches and nodes in the trajectory trees for T cells and myeloid cells, however, we did not observe a clear dynamic change of genes related to the development. These might be explained by that monocle 2 could not handle cells with too many subtypes (Response(R1) Figure 10 A-C). The new version of monocle (monocle 3 alpha and 3) could handle more complicated trajectory analysis with more different developmental states of cells (http://cole-trapnell-lab.github.io/monocle-release/monocle3/). However, too many developmental nodes identified by monocle3 alpha make it hard to resolve gene expression dynamics along pseudotime like in monocle 2 with limited nodes.*

*Alternatively, we presented the expression of genes that are known to regulate the corresponding cells in the pseudotime plots as in Fig. 2C, D and Additional file 10: Figure S7G-H in this revision. In general, we observed consistency between the development of cells and the expression of marker genes regulating the developmental process. For examples, TCF7 and LEF1 are Wnt pathway effectors and were reportedly expressed at the highest levels in naïve and central memory cells and lower levels in transitional and effector memory cells (Billingsley et al., PLoS Pathog 2015, 11:e1004740). We observed high activity and expression of TCF7 and LEF1 in CD4^+^, CD8^+^ T_N_, and T_CM_ cell clusters (Fig. 2D, E, and Figure S7G, H). FOXP3 is a specific marker of Treg cells and necessary for their suppressive activity on T cells (Hori et al., Science 2003, 299:1057-1061). We observed that FOXP3 was only enriched in CD4^+^ Treg cluster in the pseudotime plot and had higher activity in this cluster (Fig. 2D). Similarly, we observed that SPI1 had higher activity in monocytes and had higher expression level in corresponding monocyte clusters (Fig. 4F, G).*

*We have updated the results in the main text. Please kindly check in the RESULTS section in Paragraph 2, Page 8; Additional file 10: Figure S7G, H; Paragraph 2, Page 13 and Fig. 4G.*

*Response(R1) Figure 10. Pseudo-time of CD4^+^, CD8^+^ T cells, and monocytes/macrophages*

*A. Pseudo-time of CD4^+^ inferred by Monocle2 (left panel). Each point corresponds to a single cell. Clusters information was shown at the right panel. Selected genes expression along the psedutime were shown in the right panel. TCF7 and LEF1 for T_CM_ and T_N_, STAT1 and IRF1 for T_EFF_, FOXP3 for T_reg_, and FOS for T_RM_.*

*B. Pseudo-time of CD8^+^ inferred by Monocle2 (left panel). Each point corresponds to a single cell. Clusters information was shown at the right panel. Selected genes expression along the psedutime were shown in the right panel. TCF7 and LEF1 for T_CM_ and T_N_, STAT1 and TBX21 for T_EFF_, and FOS and ATF3 for T_RM_.*

*C. Pseudo-time of monocytes and macrophages, inferred by Monocle2 (left panel). Each point corresponds to a single cell. Clusters information was shown at the right panel. Selected genes expression along the psedutime were shown in the right panel. RXRN and ELF2 for classical monocytes, TCF7L2 and STAT1 for non-classical monocytes and LIPA_macrophage, and JUN and PRDM1 for most of non-classical monocyte clusters.*

Validating known markers, such as FOXP3 in Tregs, is not the purpose of trajectory analysis. As it stands now, this trajectory analysis does not lead to new biological insights and it may lead to confusion. Regulatory T cells (8) in Fig. 2C are standing next to Tcm(3). Do the authors think this makes biological sense? What is their explanation for this? Also, the central memory CD4 T cells in cluster (1) are at the end of the trajectory. What is the authors’ hypothesis regarding this feature of their trajectory?

**Response:** Thanks for the inspirational questions and suggestions. As pointed out by the Reviewer, our trajectory analysis revealed the branching out of Treg_CTLA4 (previous Treg (8)) cells next to TCM_KLF2 (previous Tcm (3)) cells along the development trajectory from TN cells to TRM cells (**Response Figure 13**). We note a similar observation reported most recently in T cells (Cano-Gamez *et al., Nat Commun* 2020, 11:1801), where human naive T cells developed towards central memory, then effector and finally highly effector memory T cells, with natural Treg cells branching out from central memory T cells. Please kindly refer to the Figure 3C in the original paper by Cano-Gamez *et a.*, which was reproduced as below **Response Figure 14** for a better reference. These observations might suggest the conversion/differentiation potential from central memory T cells to Treg cells, which has been demonstrated previously using *in vitro* models with TGF-β stimulation (Zhang *et al., PLoS One* 2013, 8(10):e77322).

**Response Figure 13**. Pseudo-time trajectory analysis of CD4^+^ T cells with high variable genes. Each dot represents a single cell and is coloured according to their clustering in the right legend. The inlet t-SNE plot shows each cell with a pseudo-time score from dark blue to yellow, indicating early and terminal states, respectively.

**Response Figure 14**. Branched pseudotime trajectory, each cell is coloured by its pseudotime value (left panel) or its cluster label (right panel). *For the purpose of quick reference, the figure was reproduced from Figure 3C in the original paper by Cano-Gamez *et al*.

Also, as pointed out by the Reviewer, a clear central memory T cell cluster (TCM_STMN1, previous Tcm (1)) is at the end of the trajectory, likely differentiated from tissue resident memory T cells (TRM_TNF, previous TRM (2)) according to the pseudotime scores. Together with that TCM_STMN1 cells were collected from lymph nodes, this observation provides a clue to solve the puzzle whether tissue-homing memory T cells further differentiate or can migrate back to the lymphoid compartment (Lees *et al., Immunology*. 2010, 130(4): 463-470). Supportively, a very recent study using mice model demonstrated that TRM cells in the skin could differentiate into TCM and TEM cells upon local reactivation, which then rejoined the circulation (Fonseca R *et al., Nat Immunol* 2020, 21:412-421). Taken together, these observations suggest that TRM cells have developmental plasticity rather than representing a terminal stage of differentiation.

Compared to other TCM clusters, TCM_STMN1 was at terminal development stage expressing mediate level of *FOS*/*JUN* and high level of *CTLA4*, an active marker of CD4^+^ T cells. To further investigate the development of the TCM_STMN1 cells, we performed trajectory analysis with TRM_TNF and TCM_STMN1 clusters, which revealed that TRM_TNF from two branches with early state gradually progressed towards TCM_STMN1 with terminal state (**Response Figure 15**). TRM cells at the beginning of the trajectory expressed high levels of TRM markers, such as *RUNX3*, *NR4A1*, chemokines (*CCL5*), and other TRM associated genes, including *ID2*. By contrast, TCM cells at the end of the trajectory was marked with expression of well-known TCM molecules, such as *SELL* and *CCR7.* In addition, some other genes that are poorly characterized were also enriched in TCM cluster, including genes encoding ribosomal proteins and *TXNIP*.

In this revision, we have added the above information in the **Discussion** section. Please check at locations (to be updated).

The authors should note that Tgd are not CD8 T cells and MAIT are thought to develop via a different route in the thymus. Please, correct the data accordingly.

The authors should also take into account that gdT are not CD8 T cells, Tcm are CCR7+ and MAIT are thought to develop via a different route.

**Response:** Thanks for the suggestions. As suggested, we had excluded the γδ T cells in the previous revision. We further excluded MAIT cells from the trajectory analysis in this revision, which revealed similar differentiation trajectories for CD8^+^ T cells compared to that in previous submission. We observed that the trajectory tree rooted from TN cells and sprouted out towards TCM, TEM, and TRM branches for CD8^+^ T cell (**Response Figure 16**). TEFF, TRM, and IEL CD8^+^ T cells showed higher pseudo-time scores, suggesting their terminal development state (**Response Figure 16**).

In this revision, we have updated the above results accordingly. In addition, we used *CCR7*, *SELL*, *TCF7*, and *LEF1* as markers for TN (naive T cell) and TCM (central memory T cell). Please kindly refer to our response to the Reviewer’s point 8 above.

**Response Figure 15**. Pseudotime trajectory analysis and gene expression profiles of TRM_TNF and TCM_STMN1.

**A**. Branched pseudotime trajectory with TRM_TNF and TCM_STMN1. Each cell as dot is coloured according to a cluster label (left panel) or pseudotime value (right panel).

**B**. Expression heatmap of selected variable genes along the pseudotime trajectory (from Monocle). Each row represents a gene as indicated at the right; column bars represent cells ordered by pseudotime (from left to right) and different colours correspond to the scaled (Z-scored) expression of each gene in each cell.

**Response Figure 16**. Pseudo-time trajectory analysis of CD8^+^ T cells with high variable genes. Each dot represents a single cell and is coloured according to their clustering in the right legend. The inlet t-SNE plot showed each cell with a pseudo-time score from dark blue to yellow, indicating early and terminal states, respectively.

Could the HSPA1A CD8+ population be a stress-induced artefact? Similar question for the Trm populations expressing FOS/JUN. How could the authors support the idea that these are not stress-induced genes?

**Response:** Thanks. We don’t think that *HSPA1A* CD8^+^ T population and the TRM populations expressing *FOS*/*JUN* are stress-induced artefacts, although the upregulation of *HSPA1A*, *FOS*, and *JUN* has been reported in certain scenarios with stress stimulations such as enzyme digestion during dissociation process (van den Brink *et al., Nat Methods* 2017, 14:935-936).

First, we had provided lines of evidence to show that dissociation processing in our study might have minimal impacts on the expression of *HSPA1A*, *FOS*, and *JUN* as well as the transcriptome of majority of cells in our dataset. Please kindly refer to our previous ***Response(R1)*** to the Reviewer’s point 12 below. The Reviewer commented that “The assessment of stress-induced transcriptomic effects is valuable and the fact that the identified clusters are not tissue-specific or dissociation-dependent is convincing.” Please kindly refer to the **Reviewer’s comment at point 5** above.

Second, the majority of *HSPA1A* CD8^+^ T cells (86.2%; 1,072 out of 1,244) were detected in the common bile duct, showing a tissue-specific distribution as reported previously in a mouse study (Beura *et al.,* *Immunity* 2018, 48:327-338 e325). The study demonstrated that *Hspa1a* was only enriched in the female reproductive tract (FRT) CD8^+^ TRM, but not in the spleen TRM and TCM cells. Moreover, although similar dissociation conditions were applied to multiple organs (**Response** **Table 1**), high expression of *HSPA1A* was observed specifically in TRM_HSPA1A and a few other cell clusters (**Response Figure 17**). Supportively, exclusively high expression of *Hspa1a* was observed in only one of two T follicular helper (TFH) like cell clusters but no other T cells types in the lung of mouse (Swarnalekha *et al., 2020 BioRxiv;* https://doi.org/10.1101/2020.02.28.963280). These observations suggest *HSPA1A* to be one of the specific markers for T cell subtypes.

Third, we suspected that high expression of *FOS* and *JUN* in CD8^+^ TRM populations (**Response Figure 17**) might reflect their role in maintaining the cells rather than in response to the dissociation-related stress. *FOS* and *JUN* are members of AP-1 transcription factor, of which dimerization has been shown to play numerous roles in a wide range of cellular process, cell growth, and proliferation, etc. We did observe the upregulation and the high regulon’s activity of many AP-1 dimerization partners (such as *FOS*, *JUN*, *JUNB*, *FOSL2*, and et) for CD4^+^ (**Response Figure 18A** and **B**) and CD8^+^ TRM cells (**Response Figure 17**, **Response Figure 18C**) in our dataset. It has been reported recently that AP-1 dimerization partners such as *Fosl2* and *Junb* were highly expressed in TRM cells, and the knockdown of *Junb* resulted in impaired TRM cell differentiation, confirming the key role of AP-1 dimerization regulating TRM differentiation (Kurd *et at., 2020* BioRxiv; <https://doi.org/10.1101/2020.03.02.973578>). Given that *Fosl2* is able to regulate *Smad3* (Ciofani *et al., Cell* 2012, 151:289-303), a key component of the TGF*β* signaling pathway, *Fosl2* may also promote TRM cell differentiation through effects on TGF*β* signaling. We note that high expression of *FOS* and *JUN* was only observed in most of TRM cells, but not non-TRM cells (**Response Figure 17, Response Figure 18A**) from the same organs that were undergone dissociation treatments with similar conditions. This observation, together with our previous observation that most organs had higher density of low total dissociation score (*Response(R1) Figure 11*), suggests that dissociation-related genes were not significantly induced in the majority of cells. Please also kindly refer to our previous response (***Response(R1))*** to the Reviewer’s point 12 below.

**Response Figure 17.** The violin plots showed the normalized expression of *FOS*, *JUN*, and *HSPA1A* in CD8^+^ T cell clusters.

Lastly and importantly, we performed additional immunofluorescence assay with double staining of CD8 and FOS in multiple organs including common bile duct, liver, and small intestine from multiple donors, and confirmed the high expression of FOS in the CD8 cells before tissue dissociation (**Response Figure 19**).

In summary, these observations suggest that *HSPA1A* CD8^+^ T population and the TRM populations expressing *FOS*/*JUN* are unlikely stress-induced artefacts. We have updated the results in this revision. Please kindly check at locations (to be updated).

**Response Figure 18**. Gene expression and cluster-specific regulon activity of T cells.

**A**. Normalized expression of *FOS* and *JUN* in different CD4^+^ T cell clusters.

**B, C**. Heatmap of the active scores of T cell subtypes (**B** for CD4^+^ T, **C** for CD8^+^ T cells, respectively), as estimated using SCENIC analysis of transcription factors (TFs). Shown on the right are the top 10 TFs with the highest difference in the expression regulation estimates between each cluster (indicated on top) and all other cells, tested with a Wilcoxon rank-sum test. Filled colours from blue to red represent scaled TF activities from low to high.

**Response Figure 19**. Representative images of immunofluorescence staining of FOS and CD8 in small intestine, common bile duct, and liver tissues. Markers used are indicated on top. Samples were derived from donor as indicated at the left. Red arrows indicated the co-expression of proteins FOS and CD8.

12) The transcription factor signature in T cells that comprised FOS, FOSB, JUN, JUND, etc is concerning since these have been previously shown to be upregulated in response to tissue dissociation-induced stress (Brink et al, 2017 Nature Methods). The authors should explain how they can exclude this is not an artifact of the tissue processing.

***Response(R1):*** *Thanks for the suggestion. We fully understand the Reviewer’s concern about the potential effect of dissociation-induced stress on cells, which should be minimal on overall transcriptome. First, we evaluated the effect of tissue processing on cells in our data, using a method described previously (the Tabula Muris consortium et al., Nature 2018, 562:367-372). We observed that most organs had higher density of low total dissociation score, meaning that dissociation-related genes were not significantly induced in the majority of cells (Response(R1) Figure 11). Second, the typical dissociation-related satellite-cell genes (van den Brink SC et al., Nat Methods 2017, 14:935-936), such FOS, HSPA1A and SOCS3, were not consistently expressed in all cell types in muscle (Response(R1) Figure 12). HSPA1A had lower expression level in both two satellite-cell clusters than other clusters. SOCS3 only slightly expressed in one of two satellite-cell clusters. Third, dissociation-related genes showed different expression pattern in a same organ where all cells were treated with a same dissociation procedure (Response(R1) Figure 13). For example, in the small intestine and stomach, FOS, JUN, and JUNB broadly expressed in different cell clusters with various degree, while SOCS3 and HSPA1A only specifically highly expressed in adoptive cell, macrophage in the small intestine, and enriched in monocyte, fibroblast, and chief cell in the stomach. Fourth, we observed that same cell types from different organs treated with different dissociation procedures had similar expression pattern of the dissociation-related genes. Taking T, B, and plasma cells in the small intestine and stomach for example, each of them had similar expression pattern of those dissociation-related genes. Moreover, each major cell type from different organs was clustered together in t-SNE plot, suggesting similar transcription profiles in spite of different dissociation treatments (Fig. 1B).*

*Taken together, the above observations suggest that dissociation processing in our study might have a minimal impact on the transcriptome of majority of cells in our dataset. Nevertheless, we could not rule out the possibility that the dissociation procedures might have impact on some certain cell types, which await further investigations. Please kindly check at Paragraph 2, Page 22 in the DISSCUSSION section. We also provided detailed information for the dissociation procedures and the evaluation of potential effects of dissociation-induced stress on the transcriptome as Supplementary Methods (Additional file 30).*

*Response(R1) Figure 11. Dissociation scores for each organ. A score represents the magnitude of the first principal component of principal component analysis based on the expression of 140 human dissociation-associated genes of each organ (van den Brink SC et al., Nat Methods 2017, 14:935-936). The y axis shows the probability density of the normalized histogram.*

*Response(R1) Figure 12. Violin plot showed the normalized expression of typical dissociation-related genes in the muscle sample. Shown were SOCS3, FOS, and HAPA1A1.*

*Response(R1) Figure 13. Violin plot showed the normalized expression of typical dissociation-related genes in the small intestine and stomach samples. Shown were FOS, FOSB, JUN, SOCS3, and HAPA1A.*

Response above in point 5.

**Response:** Thanks. Please kindly refer to point 5 above.

14) Authors use pseudotime trajectory inference on circulating monocytes and macrophages pooled from all tissues, however, they fail to take into account that most tissue-resident macrophages are not derived from circulating monocytes but from embryonic progenitors (e.g. self-renewing embryonic macrophages in the skin, liver or gut). As mentioned above, the consideration of the biological context is essential for a robust interpretation of trajectory analysis.

***Response(R1):*** *Thanks for the thoughtful comments. We fully agree with the Reviewer that most tissue-resident macrophages are derived from embryonic progenitors and undergo self-maintenance via local expansion in the steady human organ, with minimal contribution from circulating monocytes (Hashimoto et al., Immunity 2013, 38:792-804; Jakubzick et al., Immunity 2013, 39:599-610; Sieweke MH et al., Science 2013, 342:1242974).*

*In our study, we performed trajectory analysis to investigate the potential developmental relationship between monocytes and macrophages. The developmental trajectory path from classical monocytes to tissue-resident macrophages suggests that classical monocytes could be a protentional origin of adult tissue-resident macrophages (Fig. 4E). One the other hand, high expression of proliferation markers including MKI67, PCNA, and TYMS were exclusively detected in the C12 macrophages (including those in bladder, common bile duct, esophagus, heart, and rectum). Moreover, GSEA analysis also revealed that most of the macrophage clusters had higher enrichment score of G2M checkpoint and E2F targets pathways (Additional file 14: Figure S10E). These observations suggest local expansion potential of tissue-resident macrophages. Therefore, our findings suggest tissue-resident macrophages in steady-state organs might have two origins, one from classical monocytes and another from self-expansion. However, with present data, it is hard to tell that self-expansion alone or slightly together with circulating monocytes contributes to the development of tissue-resident macrophages. Further investigations using cell labeling (Chen et al., Nat Commun 2018, 9:5065) or samples from donor who received bone marrow transplantation (Thomas et al., Science 1976, 192:1016-1018) may help address the question.*

*In this revision, although our observations suggest potential development relationship between circulating monocytes and tissue-resident macrophages, we acknowledge that “however, further investigations are awaited to address whether self-expansion alone or slightly together with circulating monocytes contributes to the development of tissue-resident macrophages, considering that most tissue-resident macrophages have been demonstrated originating from embryonic progenitor cells (Hashimoto et al., Immunity 2013, 38:792-804; Jakubzick et al., Immunity 2013, 39:599-610).” Please check in Lines 10-14, paragraph 2, Page 20 in the DISCUSSION section.*

Although authors have softened the language and incorporated the possibility that tissue-macrophages may be derived from embryonic cycling macrophages, the authors could have incorporated some comparisons with published fetal data, such as gut macrophages from Gao et al., 2018 (doi: 10.1038/s41556-018-0105-4). At minimum, the authors could provide novel pathways that change in monocyte-macrophage differentiation trajectory.

Thanks for revising this part. Still, the claim should be tone down and the header of the section should exclude the “origin of macrophages” part given that this analysis cannot reliably determine the origin of these cells but simply infer transcriptional similarities among those cells.

**Response:** Thanks for the suggestions. As suggested, we obtained the raw data from Gao *et al*.’s study (*Nature Cell Biology* 2018, 20:721–734) and identified 50 embryonic macrophages in 121 immune cells from fetal large-intestine samples. We further performed trajectory analysis inclusive the embryonic macrophages and 828 cells of monocytes and macrophages from rectum sample in our AHCA data (**Response Figure 20A**). Pseudotime analysis revealed that both embryonic and adult macrophages were at terminal state (**Response Figure 20A**). Moreover, we observed a clear differentiation trajectory of the macrophages in adult rectum from intermediate monocytes (Intermediate_Mon_CCL20) to tissue macrophages. Interestingly, the embryonic macrophages (Mac_embryo) were found alongside the differentiation trajectory from the intermediate monocyte (Intermediate_Mon_FN1) to the tissue-macrophages (Mac_HIST1H4C), suggesting that embryonic macrophage may contribute to the tissue-macrophage through local expansion. Consistently, high expression of proliferation marker gene *PCNA* were detected in both the terminal macrophages (**Response Figure 20B**).

In this revision, we have added the above comparisons in the **Results** and **Discussion** sections as well as the related methods in the **Methods** section. Please kindly check at locations (to be updated). In addition, we removed “origin of macrophages“ from the header as suggested.

**Response Figure 20**. Development trajectory of monocytes and macrophages in the intestine tissues.

**A**. Pseudo-time trajectory analysis of myeloid cells from rectum tissue in our AHCA dataset and macrophages from embryo large intestine in Gao *et al*.’s data. Each dot represents a single cell and is coloured according to their cluster. Only cell clusters with large than 50 cells in AHCA were included. The inlet t-SNE plot shows each cell with a pseudo-time score from dark blue to yellow, indicating early and terminal states, respectively.

**B**. Violin plots showed the normalized expression of proliferation marker genes (*MKI67* and *PCAN*) in each myeloid cell cluster as indicated at the x axis in the combined data of AHCA and Gao *et al*.’s samples.

15) Epithelial cell analysis is focused on biology that has been previously described. The authors could use this dataset to further understand the core-similarities between epithelial cell subsets (e.g. secretory cells) across tissues as well as the key differences.

***Response(R1):*** *Thanks for the suggestion. As suggested, we performed additional analyses to examine the similarities of epithelial cells across different organs. First, we compared TFs in epithelial cell clusters, which revealed similar activation of TFs in most non-digestive clusters, as well as in digestive clusters (Fig. 5G). This was further confirmed using a recent published dataset (Additional file 19: Figure S13B). Second, pathway analysis revealed that multiple metabolic pathways were enriched in digestive clusters other than non-digestive cluster, suggesting the enhanced metabolic activity of digestive epithelial cells (Additional file 18: Figure S12C, D). Lastly, we performed ligand-receptor analysis for epithelial cells across different organs, which revealed that epithelial cells had the most frequent intra-cell interactions among all cell types, suggesting that epithelial cells could interact with each other intensively to regulate their biology functions in each organ (Additional file 24: Figure S17C and Additional file 19: Figure S21).*

*In this revision, we added the new results in the main text. Please kindly check in in Line 4-6, Paragraph 2, Page 14; Line 8-11, Paragraph 3, Page 14; Line 3-5, Paragraph 1, Page 15; Line 6-11, Paragraph 2, Page 15; Line 5-9, Paragraph 1, Page 16; Line 9-11, Paragraph 1, Page 21; and Line 16-18, Paragraph 2, Page 21.*

The authors should clarify which tissues do they classify as digestive tissue and what are the implications of this observation.

The detection of a “humoral immune response” GO term in epithelial cells suggests that there is a contamination in these cells. One possibility could be that ambient mRNA derived from lysed plasma cells led to the detection of plasma cell transcripts in these epithelial cells. Please, revise this point.

**Response:** Thanks for the thoughtful suggestions. According to the presence of digestive-related functions, stomach, small intestine, rectum, liver, and common bile duct were classified as digestive tissue in our AHCA data (**Response Figure 21A**), as were colons (ascending colon, colon, duodenum, sigmoid colon, transverse colon), small intestines (duodenum, Ileum, Jejunum), liver, gallbladder, pancreas, stomach, and rectum in the Han *et al.*’s HCL dataset (**Response Figure 21B**). By contrast, all other tissues with detectable epithelial cells were considered as non-digestive tissue. Although esophagus is a part of digestive tract, epithelial cells from the tissue shared similar expression profiles with that in skin and trachea in both the AHCA and HCL datasets, indicating similar function of epithelial cells in esophagus compared to skin and trachea, but not other digestive organs (**Response Figure 21A, B**).

First, we investigated the contribution of TFs in regulating the heterogenous transcriptional profiles of epithelial cells in and between organs. SCENIC analysis revealed that the regulation of TFs was similar among cell clusters derived from a same tissue but was very different among cell clusters between tissues in both the AHCA and HCL dataset (**Response Figure 22**). Interestingly, the digestion-associated epithelial clusters exhibited similar activation of TFs, as did the cell clusters belong to non-digestive tissues including the trachea, skin, and esophagus (**Response Figure 22A**). Similar activation of TFs was observed between our AHCA and the HCL datasets for digestive-associated tissues (expect for pancreas) and non-digestive tissues (except for lung; **Response Figure 22B**). Moreover, we identified 204 TFs in our AHCA dataset, which overlap about 60% (120) of all cluster-specific TFs of the HCL dataset. Second, GO analysis revealed that distinct biological functions of cells between digestive tissue and non-digestive tissue. Metabolic process, energy synthesis, and digestion pathways were commonly observed in digestive tissue (**Response Figure 23A**), while *regulation of endopeptidase activity* and *epidermis development* were the strongly enriched pathways observed in non-digestive tissue (**Response Figure 23B**). Third, GSVA analysis also revealed significantly different pathways between digestive and non-digestive cell clusters, including multiple metabolic pathways with elevated activity in digestive cells and *TNFA signal via NFKB* in non-digestive epithelial cells (**Response Figure 21 C** and **D**). These observations indicate the diverse molecular profiles underlying the functional heterogeneity between the two groups of tissues, as well as the similarity in gene profiles among different tissues of same function. We note that most of the digestion-related organs developed from endoderm, which might explain their similarity in gene profiles and functions (Zorn AM *et al., Annu Rev Cell Dev Biol* 2009, 25:221-251). In addition, we suspected that the epithelial cells of same digestive functions might also share similar response to pathogens or stimulus (Gunther *et al., Semin Immunopathol* 2018, 40:555-565).

As suggested, we have removed genes encoding immunoglobulins from the downstream analysis in this revision. We have also updated the above information in the **Methods**, **Results**, and **Discussion** sections. Please kindly check at locations (to be updated).

**Response Figure 21**. Comparisons of gene profiles and functions of cells between digestive and non-digestive organs.

**A, B**. Phylogenetic tree of tissues in AHCA (**A**) or Han *et al.*’s HCL (**B**) dataset, based on their normalized mean expression values of epithelial cells (correlation distance metric, complete linkage). Only genes with ln(fold-change) above 0.25, *p.adjust* < 0.05 and pct.1 ≥ 0.2 in each organ were included in the analysis. Organs with less than 50 cells were excluded.

**C, D**. GSVA analysis showed the pathways (hallmark and curated metabolic pathways) with significantly different activation between non-digestive and digestive cell clusters in both the AHCA (**C**) and Han *et al.*’s HCL (**D**) dataset. Activation was determined using GSVA and comparison was tested with an R package limma.

**Response Figure 22**. Heatmap of scaled transcription factor activities.

**A, B**. Heatmap of the active scores for epithelial cell subtypes as numbered on top in our AHCA (**A**) and Han *et al.*’s HCL (**B**) datasets. Activation score was based on the expression regulated by transcription factors (TFs), as estimated using SCENIC analysis. Shown are the top 10 TFs having the highest difference in expression regulation estimates between each cluster and all other cells, tested with a Wilcoxon rank-sum test. TFs with similar activity across multiple digestive or non-digestive organ-derived clusters were outlined in green dashed boxes.

**Response Figure 23**. GO analysis results of each epithelial cell cluster.

**A, B**. Gene Ontology enrichment analysis results of each epithelial cell cluster in digestive organs (**A**) and non-digestive organs (**B**). Cell clusters were coloured according to their -log_10_(P values). Only the top 20 significant terms (*p-value* < 0.05) were shown.

**Third round of review**

**Reviewer 2**

We appreciate the effort made by the authors and would now support the publication of this work. See below final suggestions in dark red.

**Response:** We are very appreciative of your support to the publication of our work as well as thoughtful suggestions. In this response letter, please kindly refer to below points for our responses (in blue) to the suggestions; and we have synchronized the name of cell cluster with that in the main text and updated the locations of the corresponding content in the revised manuscript. Also, we have addressed and incorporated all the comments and suggestions in the revised manuscript.

**Please note that all comments from the Reviewer #1 had been fully addressed and we only keep the comments from the Reviewer #2 yet to be fully addressed, together with the original comment from the Reviewer #2 (in black), our *previous Response(R1)* (in *black* and *Italic*) in the first revision, additional comments from the Reviewer #2 (in underlined and black), and our new response (in Blue), for better following-up.

**Reviewer #2:**

Summary:
He et al have compiled a single-cell RNA-seq and VDJ-seq dataset including 15 different organs from a single individual. The authors describe cell types and patterns of B and T cell clone distributions across organs. While many of the findings are expected, the authors make some interesting observations, however, the lack of replicates and experimental validation weakens any conclusion regarding novel and/or rare cell types.

***Response(R1)****: Thanks for the positive comment. In this revision, we have provided additional analyses and experimental validation for the identification of novel and/or rare cell types, which could be found below as to address the Reviewer’s comments.*

Overall, the authors have addressed many of the issues raised by the reviewers and the manuscript has improved significantly upon revision, however, there are still major concerns that preclude its publication following Genome Biology standards. Language and terminology should be carefully revised throughout the manuscript. See below for further details.

**Response:** Once again, we have been grateful to the Reviewer for thoughtful suggestions that helped improve our manuscript significantly. We fully understand that *Genome Biology* is one of the top journals in research areas of biology and biomedicine and we have been trying our best to present this work to a broader readership so that it would contribute useful information to the scientific community.

In this revision, we have improved further our manuscript with additional analyses and validation experiments according to the Reviewer’s suggestions. Moreover, we have carefully revised the manuscript, with language editing by a native English speaker and terminology confirmation/revision.

Ok, thank you.

Major comments:

2) This study has profiled only one donor. Given the high biological variation in humans, the applicability of these findings to the broader population are under question.

***Response(R1):*** *Thanks for the pertinent comment. We fully understand the Reviewer’s concern if our findings could be applied to broader population in the context of biological variations among individuals. In our study, thousands of cells from multiple organs were analyzed, which enables us to understand the heterogeneous composition of cells in human organs and the development links among cell types, and to identify important molecules and their networking. Such as, similar with the report by The Tabula Muris Consortium et al. (Nature 2018, 562:367-372), we observed close clustering of cells from different organs (more than seven organs) for major cell types, including T cells, B cells, NK cells, fibroblasts, endothelial cells, smooth muscle cells, macrophages, and monocytes. We also identified several well characterized TFs regulating the different cell development, including TCF7, SELE, MYC, and KLF2 for T_N_ cells, TBX21, STAT1, and IRF1 for T_EFF_ cells, and PRDM1, XBP1, FOS, and IRF4 for plasma cells. The consistencies between our findings and previous findings suggest that our study based on large scale transcriptomes at single cell level provides common biological understanding at higher resolution.*

*Moreover, we used an independent set of single cell sequencing data derived from human organs, which was published very recently (Han et al. Nature 2020:581, 303-309), and we managed to replicate our findings. First, we identified important transcription factors regulating plasma cell development, such as XBP1, MYC, etc., which have similar activation patterns in both datasets (Fig. 3E and Figure S9F). Second, epithelial cells from different organs showed strong heterogeneity, while epithelial cells within the same tissue had similar gene expression profile and clustered together, in both datasets (Fig. 5A and Figure S11B). Third, we found that both digestive and non-digested tissues showed similar transcriptional factor activities in both datasets (Fig. 5G and Additional file 19: Figure S13B). In addition, we note that we only selected B cells and epithelial cells for replication analyses, because the recent published data has low sequencing depth and extensive contamination between different cell types (Han et al., Nature 2020:581, 303-309).*

*Furthermore, we validated our discovery of novel and/rare cell types using experiment as suggested by the Reviewer #2, including sweat gland epithelial cell in skin, COCH^+^ fibroblast in the skin and heart, and FibSmo in the bladder, heart, and rectum in independent samples (Figure S3C and Figure S4A). Please check more details regarding this at below Reviewer’s comment point 4 (Response(R1) Page 38).*

*Taken together, although our data was derived from one donor, some important findings based on large number of cells and multiple organs might be generally applicable. However, we do acknowledge that further investigations with more individual samples will solidify our findings and discover more novel cell types, key molecules, and interaction network.*

*We have added the replication analyses and experimental results as well as limitation in this revision. Please kindly check in the RESULTS section in Paragraph 2, Page 6; Paragraph 1, Page 11; Line 4-7, Paragraph 2, Page 14; Line 8-13 Paragraph 3, Page 14; Line 3-6, Paragraph 1, Page 15; Line 6-12, Paragraph 2, Page 15; and Line 5-9, Paragraph 1, Page 16.*

The points mentioned above are based on well-established human cellular biology, e.g. XBP1 in plasma cell differentiation. This reviewer was rather referring to validating novel findings in either newly generated or existing datasets. The incorporation of the Han et al HCL dataset is a valuable addition, however, the fact that this had to be restricted to only 2 cell types is sub-optimal. There are other available datasets of higher quality that could be used for validation of the COCH+ fibroblasts (e.g. heart datasets: Tucker et al. 2020 Circulation; Litvinukova et al. 2020 biorxiv).

Please, indicate what does FibSmo stand for.

**Response:** Thanks for the suggestions. As suggested, we performed additional analyses using several existing public datasets and multiplex immunofluorescence staining in more human tissue samples to validate our novel findings.

First, we validated the presence of *COCH*^+^ fibroblasts in multiple skin and heart tissues (**Response Figure 1**), using two skin datasets (Solé-Boldo *et al., Communications Biology* 2020, 3:188 and He *et al., Journal of Allergy and Clinical Immunology* 2020, 145:1615-1628 and) and one heart dataset (Tucker *et at., Circulation* 2020) that are publicly available. Please note that we were not able to access the suggested dataset from Litvinukova *et al*. We observed a clear clustering of *COCH*^+^ fibroblasts with co-expression of *MMP2* and *COCH* genes in the skin dataset of Solé-Boldo *et al.*’s (cluster 30; **Response Figure 1A:** left top and **B:** left). A total of 144 *COCH*^+^ fibroblasts (out of 7,298 fibroblasts and 7,879 other cells in the skin) were identified in 5 skin samples in the dataset, with maximum 103 cells for one sample. We also observed a small group of *COCH*^+^ fibroblasts with high expression of both *COCH* and *MMP2* in the skin dataset of He *et al.*’s (cluster 18; **Response Figure 1A:** right and **B:** middle). A total of 125 *COCH*^+^ fibroblasts (out of 5,657 fibroblasts and 19,669 other cells in the skin) were identified in 14 skin samples in the dataset, with maximum 31 cells for one sample. By contrast, 454 *COCH*^+^ fibroblasts cells scattered among a total of 62,292 fibroblasts from the 26 heart samples in Tucker *et al*.’s dataset, with maximum 45 cells for one sample (clusters 1, 2, 4, 6, 7, 10; **Response Figure 1A:** left bottom and **B:** right). Noteworthily, we further validated the presence of *COCH*^+^ fibroblasts in more human tissue samples using multiplex immunofluorescence staining assays (Please kindly refer to Reviewer #2’s comment 4 below).

**Response Figure 1.** Expression of *COCH* and *MMP2* in cells from the skin and heart samples.

**A**. Violin plots showed the normalized expression of *COCH* and *MMP2* in cells of different clusters from the skin (top) and heart (left bottom) tissues retrieved from public datasets as indicated. Clusters were numbered at x-axis, and the expression level was shown in y-axis. Each dot represents a single cell.

**B**. Feature plots showed the normalized expression of *COCH* and *MMP2* in cells from skin (left and middle) and heart (right) tissues retrieved from public datasets as indicated at the bottom.

Next, we observed similar gene signatures of *COCH^+^* fibroblasts between our AHCA dataset and Solé-Boldo *et al.*’s dataset, compared to all other cells in respective dataset (**Response Figure 2**). 31 out of 50 upregulated genes in *COCH^+^* fibroblasts were shared between the two datasets, among which *COCH* and *ASPN* are the two most significant ones (**Response Figure 2**). Both *COCH* and *ASPN* are secretory proteins in the extracellular matrix and have been involved in extracellular matrix remodelling (Cho *et al., J Mol Med (Berl)* 2012, 90:1321-1331; Goel *et al., PLoS One* 2011, 6:e23070; Ikegawa, *Curr Med Chem* 2008, 15:724-728), suggesting the important role of *COCH^+^* fibroblasts in maintaining tissue homeostasis.

Second, we validated the presence of *SCGB2A2*^+^ sweat gland cells in multiple skin samples from the above two datasets (**Response Figure 3**). We observed a clear clustering of *SCGB2A2*^+^ sweat gland cells with co-expression of *DCD*, *SCGB2A2*, *PIP*, *KRT19*, and *MUCL1* genes in the skin dataset of He *et al.*’s (cluster 25; **Response Figure 3**: left panels). A total of 346 *SCGB2A2*^+^ sweat gland cells (out of 25,326 cells in the skin) were identified in 14 skin samples in the dataset, ranging from 5 to 102 cells for each individual sample. By contrast, we only observed one *SCGB2A2*^+^ sweat gland cell with co-expression of *DCD*, *SCGB2A2*, *PIP*, *KRT19*, and *MUCL1* genes in Solé-Boldo *et al*.’s dataset (cluster 28; **Response Figure 3:** right panels), as limited epithelial cells (ranging from 99 to 969) were collected from each donor in the study.

**Response Figure 2.** Gene signature of *COCH*^+^ fibroblasts in skin.

**A, B**. Scatter plots showed the differentially expressed genes compared to other cells in skin samples from our AHCA or Solé-Boldo *et al*.’s datasets. Selected genes were labelled if ln(fold-change) greater than 4 and 3 for upregulation (in red), or smaller than 0.25 and 0.33 for downregulation (blue) in the AHCA and Solé-Boldo *et al*.’s datasets, respectively.

**C.** Venn diagram showed the sharing of upregulated genes in *COCH^+^* fibroblasts in comparison to all other cells.

**Response Figure 3.** Expression of *DCD*, *SCGB2A2*, *PIP*, *KRT19* and *MUCL1* genes in cells from skin samples.

**A**. Violin plots showed the normalized expression of *DCD*, *SCGB2A2*, *PIP*, *KRT19*, and *MUCL1* in cells of different clusters from the skin tissues retrieved from public datasets as indicated. Clusters were numbered at x-axis, and the expression level was shown in y-axis. Each dot represents a single cell. The clusters for *SCGB2A2*^+^ sweat gland cells were boxed.

**B**. Feature plots showed the normalized expression of *DCD*, *SCGB2A2*, *PIP*, *KRT19*, and *MUCL1* in cells from skin tissues retrieved from public datasets as indicated at the bottom.

Third, we validated the presence of *MMP2*^+^*ACTA2*^+^ cells (assigned as FibSmo cells) in multiple bladder samples from two independent public datasets (Yu *et al., J Am Soc Nephrol* 2019, 30:2159-2176; Han *et al., Nature* 2020, 581:303-309; **Response Figure 4**). We observed a clear clustering of FibSmo cells with co-expression of *MMP2* and *ACTA2* genes in both datasets. A total of 1,447 FibSmo cells (out of 13,490 cells in the bladder) were identified in 3 bladder samples in the Yu *et al*.’s dataset, ranging from 42 to 709 cells for each individual sample (**Response Figure 4:** left panels). Similarly, a total of 178 FibSmo (out of 1,193 stromal cells in the bladder) were identified in 2 bladder samples in the Han *et al.*’s dataset, with 24 and 154 cells in each sample (**Response Figure 4:** right panels). Moreover, we confirmed the presence of FibSmo cells in multiple human samples using immunofluorescence staining assays. Please kindly refer to the Reviewer’s comment 4 below.

In summary, we confirmed the presence of *COCH*^+^ fibroblasts, *SCGB2A2*^+^ sweat gland cells, and FibSmo cells in multiple human tissue samples using additional existing datasets and immunofluorescence staining assays.

We named FibSmo cells as a novel cell type with co-expression of *MMP2* and *ACTA2*, which are marker genes for fibroblast and smooth muscle cells, respectively. Although FibSmo cells share marker genes with the two cell types, they had distinct gene signatures (**Response Figure 5A** and **B**). FibSmo cells exhibit high and specific expression of *PLAT* and *ID1,* which were related to clotting and angiogenesis (Carmeliet *et al., Nature* 1994*,* 368:419-424; Volpert *et al., Cancer Cell* 2002, 2:473-483), and higher expression of *COL3A1* and *COL1A1* compared to other stromal cells, which have been involved in wound healing (Crane *et al., Wound Repair Regen* 2010, 18:409-416). In addition, the enrichment of BMP signal genes *BMP4* and *BMP5* was also observed in the FibSmo cells (**Response Figure 5B**). GO analysis revealed that FibSmo cells shared common stromal functions with fibroblasts and smooth muscle cells, such as *extracellular structure organization* and *tissue remodelling*, while FibSmo cells had enhanced biological functions in *response to wounding* and *response to growth factor*, which is consistent with the functions of their highly expressed signature genes above (**Response Figure 5C**).

In this revision, we have added the above validation results and a more detailed description of FibSmo cells in the main text (please kindly check at the lines 8-10 and the last sentence in the 2^nd^ paragraph, Page 6; the lines 3-5, 16-26 in the 1^st^ paragraph, Page 18) and the Appendix (**Supplementary Figure S4-S10**, **Supplementary Figure 23**, and **Additional file 7 Supplementary Notes**). We also updated the related **Methods** (please kindly check at the 3^rd^ paragraph, Page 31 to the 2^nd^ paragraph, Page 32; the 1^st^ paragraph, Page 33).

**Response Figure 4**. Expression of marker genes in cells from bladder samples.

**A**. Violin plots showed the normalized expression of *MMP2*, *ACTA2*, *BMP4*, *BMP5*, and *PLAT* in cells of different clusters from the bladder tissues retrieved from public datasets as indicated. Clusters were numbered at x-axis, and the expression level was shown in y-axis. Each dot represents a single cell. The clusters for FibSmo cells were boxed.

**B**. Feature plots showed the normalized expression of *MMP2*, *ACTA2*, *BMP4*, *BMP5*, and *PLAT* in cells from skin tissues retrieved from public datasets as indicated at the bottom.

**Response Figure 5.** Gene expression and estimated functions of fibroblast, FibSmo, and smooth muscle cells.

**A**. Heatmap showed the average expression level of top 150 genes enriched in fibroblast, FibSmo, and smooth muscle cells as indicated in rows. Each column line represents one gene and the expression level from high to low was coded as red to blue, respectively.

**B**. Violin plots showed the expression of marker genes of FibSmo in the three cell types as indicated at the bottom.

**C**. Bar plots showed the selected biological functions enriched in fibroblast, FibSmo, and smooth muscle cells. Key genes were listed for enhanced GO term of different cell types.

We appreciate the effort the authors have made to support their findings with pre-existing datasets and consider that this additional line of evidence makes the results more robust. Since the expression of ACTA2 and MMP2 was seen in multiple clusters of existing datasets, it would be useful if authors could include BMP4, BMP5, PLAT in the Response Figure 4.

**Response:** Thanks for the positive comment and the thoughtful suggestion. As suggested, we included the expression levels of *BMP4*, *BMP5*, and *PLAT* in the above **Response Figure** **4**. We observed consistently high expression of *BMP4*, *BMP5*, and *PLAT* in FibSmo cells in the validation datasets. We also updated the results in **Supplementary Figure 7** in the revised manuscript.

4) The identification of rare/novel cell types (e.g. Langerhans cells and COCH+ fibroblast subtypes) should be supported by replicates/validation.

***Response(R1):*** *Thanks for the comment. To address the comment, we performed immunofluorescence staining assays to confirm the presence of rare/novel cell types identified in our study. We managed to locate sweat gland epithelial cell in two independent skin samples, which were stained with makers KRT19, SCGB2A2, and PIP (Additional file 5: Figure S3C). Similarly, we validated the novel fibroblast subtype by staining markers COCH and MMP2 in the skin and heart samples from two independent individual donors (Figure S3C). Importantly, we confirmed the novel cell type FibSmo with markers MMP2 and ACTA2 in the bladder, heart, and rectum samples from two independent individual donors (Figure S4A). Please note that we don’t examine the presence of Langerhans cell in tissue samples, because this cell type has been widely reported (Merad et al., Nat Rev Immunol 2008, 8:935-947; Clayton et al., Front Immunol 2017, 8:1676).*

*We have added the above results in this revision. Please kindly check in Line 15-18, Paragraph 2, Page 6 in the RESULTS section.*

The immunofluorescence results in S3C and S4A show only one of the two donors and some of the stainings are dubious since they look like background signal, e.g. SCGB2A2). In addition, the genes chosen as specific to rare populations instead seem to be expressed across many cells imaged.

Did the authors perform appropriate IF controls? Did the authors also report the results of the second donor? Did the authors also detect these novel cell types in the Human Cell Landscape from Han et al 2020 or other datasets? Do the authors have any hypothesis as to which could be the function of the novel fibroblast subtypes?

**Response:** Thanks for the suggestions. Yes, we previously provided representative immunofluorescence (IF) images for only one donor in the previous Figure S3C and S4A, although the IF assay was done in samples from two donors. To address the Reviewer’s comments, we have performed IF assays with more samples. All the results from multiple donors were provided as Supplementary Figures.

For sweat gland epithelial cell, we repeated multiplex IF assays in the two donors (donors 1 and 2) as in our previous manuscript. Again, co-localization of SCGB2A2, KRT19, and PIP proteins were identified for the *SCGB2A2*^+^ sweat gland epithelial cells (**Response Figure 6**). We note that the expression of SCGB2A2 is profound in skin cells but is limited to a certain cell type. This is consistent with the observation in another skin sample (donor 3), which was stained with only SCGB2A2 as a positive control (**Response Figure 6**).

For FibSmo cells, we performed multiplex IF assays in additional samples and presented the representative IF images for all samples. We observed co-localization of MMP2 and ACTA2 proteins in numbers of FibSmo cells in bladder (4 donors), heart (2 donors), and rectum samples (2 donors; **Response Figure 7**). FibSmo shared common stromal functions with fibroblasts and smooth muscle cells, such as *extracellular structure organization* and *tissue remodelling*, while FibSmo cells had enhanced biological functions in *response to wounding* and *response to growth factor*, which is consistent with the functions of their highly expressed signature genes (**Response Figure 5C**). For more information regarding the gene expression signatures and functions, please kindly refer to the Reviewer’s comment 2 above.

For *COCH*^+^ fibroblasts, we also repeated the multiplex IF analysis in the previous two donors, which revealed co-localization of COCH and MMP2 staining in the 4 skin and heart samples (**Response Figure 8**).

In summary, the multiplex IF assays in additional samples confirmed the presence of *SCGB2A2*^+^ sweat gland epithelial cells, FibSmo cells, and *COCH*^+^ fibroblasts in human. Consistently, the presence of such cell types was also validated in human samples with publicly available single cell sequencing data. Please kindly refer to our response to the Reviewer’s point 2 above. We note that the identifications of these cell types were based on co-expression of multiple cell markers, which is not specifically expressed for a certain cell type. Therefore, it is plausible that we might observe a broad expression of one of these markers across different cell types. We acknowledge that further investigations could be done to identify more specific markers for these cell types.

**Response Figure 6**. Representative images of immunofluorescence staining in skin samples for sweat gland epithelial cells. Proteins used in the assays are indicated at the left, and the samples from different donors (1-3) were listed as columns. The red arrows indicated the co-expression of SCGB2A2, PIP, and KRT19 proteins in cells in donor 1 and 2. Donor 3 was severed as positive control of SCGB2A2 staining. The red arrows indicated the specifically expression of SCGB2A2 in certain cell types in the skin in donor 3.

**Response Figure 7**. Representative images of immunofluorescence staining for FibSmo cells in bladder, rectum, and heart tissues. Proteins used in the assays are indicated on top, and the bladder, heart, and rectum samples from different donors (1-4) were listed as rows. The red arrows indicated the co-expression of ACTA2 and MMP2 proteins in tissue cells. For each sample, the right panel highlighted in a box was imaged field different from the left panel on the same tissue slide.

**Response Figure 8**. Representative images of immunofluorescence staining for *COCH*^+^ fibroblast cells in skin and heart tissues. Proteins used in the assays are indicated on top, and the skin and heart samples from different donors (1-2) were listed as rows. The red arrows indicated the co-expression of COCH and MMP2 proteins in cells of samples as indicated at the left. For each sample, the right panel highlighted in a box was imaged field different from the left panel on the same tissue slide.

We thank the authors for the additional data. Still, it remains hard to interpret the data given the lack of negative controls and negative cells within the imaged field. The authors should ensure that the detected signals are specific with appropriate negative controls.

**Response**: Thanks for the comment and suggestion. We fully understand the Reviewer’s concern on the specificity of an immunostaining assay. In our study, we ensured that the immunofluorescence staining signals were specific to each protein of interest. We did observe specific staining of SCGB2A2, PIP, and KRT19 proteins in sweat gland epithelial cells (above **Response Figure 6**) and MMP2 protein in tissue cells (above **Response Figures 7** and **8**), with a clear distribution of both positive and negative cells in the same imaged fields. By contrast, we observed broad expression of ACTA2 and COCH in tissue samples. This might reflect the abundant endogenous expression of the proteins. Indeed, ACTA2 protein has been shown with broad expression in the bladder and rectum tissues according to the Human Protein Atlas portal (https://www.proteinatlas.org/ENSG00000107796-ACTA2/tissue/rectum; https://www.proteinatlas.org/ENSG00000107796-ACTA2/tissue/urinary+bladder). Moreover, we provided additional images for the same tissue slides for each sample, where we observed more negative staining of cells compared to the previous imaged fields (above **Response Figures 7** and **8:** right panels). Furthermore, we performed IF with negative controls and observed no staining signals of ACTA2 and COCH in the tissue samples (**Supplementary Figures 9** and **10**, reproduced as **Additional** **Response Figure 1** below). Together, these observations indicate that the staining of proteins of interest are specific.

**Additional** **Response Figure 1.** Representative images of negative controls for immunofluorescence staining assays. Multiplex IF assays were performed using the same protocols as in **Response** **Figures** **7** and **8** but without primary antibodies recognising COCH (**A**) and ACTA2 (**B**). Tissues of origins are shown on top. Tissue sections were derived from the identical tissue samples of Donor 1 as in above **Response Figures 7** and **8**.

5) The authors should provide a complete description of the tissue dissociation method. In addition, they should comment on how the use of different dissociation protocols could result in selective release of cells in different organs and/or induction of stress response.

***Response(R1):*** *Thanks for the suggestions. As suggested, we now provide a detailed description of tissue dissociation protocols in Additional file 30, which includes the procedures as well as the use of enzymes and treatment durations for each organ. We note that these disassociation protocols were established based on the previous publications and rounds of in-house tests on different pig and human tissues until we could achieve high cell viability, less digestive time and less residual tissue.*

*As pointed out by the Reviewer, cellular dissociation was a prerequisite technical manipulation for single cell studies, although dissociation procedures would cause disturbance of transcriptome at various degrees (Brink et al., Nat Methods 2017, 14:935-936). Enzymes and duration of dissociation procedures might be two important factors (Harris et al., Hippocampus 2019, 29:876-882; Brink et al., Nat Methods 2017, 14:935-936). A gentle enzyme and short time might help to minimize the disturbance of transcriptome or induction of stress response. Moreover, an organ consists different cell types and extra cellar matrix, which need different enzymes to dissociate them into single cells. It’s unlikely that one enzyme could fit for all organs or cell types. Long duration and high concentration of enzymes could have a higher degree of dissociation. However, it comes with higher level of damage on cells and disturbance of transcription. Collagenase could only degrade the triple-helical native collagen fibrils; Dispase cleaving fibronectin, type IV collagen and type V collagen are gentle and have limited damage to cells; and Trypsin could cleave polypeptide chain, but could damage the integrity of cell membranes and reduce cell viability when treating tissues with a long duration. Considering the properties of different enzymes and the heterogeneous cell types in organs, we have optimized the protocols to achieve better dissociation and higher cell viability for each organ. We observed well clustering of the major cell types independently, which derived from different organs, suggesting that the dissociation procedures had minimal effects on the transcriptional profiles for the majority of cells. For more evidence, please refer to our response to the below comment Point 12 (Response(R1) Pages 48-52). However, we could not rule out the possibility that the dissociation procedures might have impact on some certain cell types, which await further investigations.*

*As suggested, we made comments in the DISCUSSION section. Please kindly check in Paragraph 2, Page 22. We also provided detailed information for the dissociation procedures and the evaluation of potential effects of dissociation-induced stress on the transcriptome as Supplementary Methods (Additional file 30).*

Thank you for adding the detailed protocols. They could be summarized in a table as there are high similarities across organs. The table can display the exact conditions for each organ. Indicate the concentration of dispase, not just the volume. Correct “disassociation protocols” for “dissociation” and “HAPA1A” for “HSPA1A”.

**Response:** Thanks for the suggestions and pointing out the typos. As suggested, we provided a supplementary table to summarize dissociation protocols for each organ, including primary dissociation reagents, addition of CaCl_2_, dissociation buffer, incubation condition and extra dissociation. Please kindly check at **Supplementary Table 51**, which was reproduced as below. As such, in this revision we have deleted the literal protocols described in the previous submission. In addition, we have corrected the typos in this revision.

**Response Table 1**. The digestion protocols for each organ.

| **Tissue** | **Primary dissociation reagents** | **Addition of CaCl_2_** | **Dissociation buffer** | **Incubation condition** | **Extra dissociation (optional)** |
| --- | --- | --- | --- | --- | --- |
| **Blood** | 0.8% NH_4_Cl red blood cells lysis buffer | NO | RBC buffer | 10 min on ice | NO |
| **Bone marrow** |  |  |  |  |  |
| **Bladder** | 125 U Dispase/5 ml + 1 mg/ml Collagenase IV + 1 mg/ml Collagenase II + 50 ug/ml DNase I | 5 mM | DMEM + 10%FBS | 1 h + 10 min | 0.25% trypsin + EDTA 20 min for residue |
| **Common bile duct** |  |  |  | 40 min + 10 min |  |
| **Esophagus** |  |  |  | 1 h + 10 min |  |
| **Heart** | 50 U Dispase/5 ml + 1 mg/ml Collagenase IV + 1 mg/ml Collagenase II + 50 ug/ml DNase I | 5 mM | DMEM + 10%FBS | 40 min + 10 min |  |
| **Lymph node** |  |  |  |  |  |
| **Muscle (leg)** |  |  |  |  |  |
| **Spleen** | 0.5 mg/ml Collagenase IV + 0.5 mg/ml Collagenase II + 50 ug/ml DNase I | 5 mM | DMEM + 10%FBS | 30 min + 10 min |  |
| **Small intestine** | 0.05% Trypsin + EDTA+ 0.5 mg/ml Collagenase IV + 0.5 mg/ml Collagenase II + 50 ug/ml DNase I | 5 mM added after 20 min | DMEM | 1 h + 10 min |  |
| **Rectum** | 0.05% Trypsin + EDTA+ 1 mg/ml Collagenase IV + 1 mg/ml Collagenase II + 50 ug/ml DNase I | 5 mM added after 20 min | DMEM | 1 h + 10 min |  |
| **Stomach** |  |  |  |  |  |
| **Liver** | Step 1: 1 mM EGTA + 1xDPBS, Step 2: 1 mg/ml Collagenase II + 1 mg/ml Collagenase IV + 50 ug/ml DNase I | 5 mM added after 10 min | Step 1: 1x DPBS, Step 2: DMEM + 10%FBS | Step 1: 10 min, Step 2: 30 min + 10 min |  |
| **Skin** | Step 1: 0.25% Trypsin + EDTA, Step 2: 125 U Dispase/5 ml + 1 mg/ml Collagenase I + 1 mg/ml Collagenase V + 50 ug/ml DNase I | 5 mM added after 20 min | DMEM | Step 1: 20 min, Step 2: 1 h + 10 min |  |
| **Trachea** | Step1: 0.25% Trypsin + EDTA, Step 2: 125 U Dispase/5 ml + 1 mg/ml Collagenase IV + 1 mg/ml Collagenase II + 50 ug/ml DNase I | 5 mM added after 20 min | DMEM | Step 1: 20 min, Step 2: 1 h + 10 min |  |

Ok, thank you.

The assessment of stress-induced transcriptomic effects is valuable and the fact that the identified clusters are not tissue-specific or dissociation-dependent is convincing. Nonetheless, the second and third points raised in pages 9 and 10 of the additional file 10 (“…FOS, HSPA1A and SOCS3, were not consistently expressed in all cell types in muscle…” and “dissociation-related genes showed different expression pattern in a same organ where all cells were treated with a same dissociation procedure”) are not conclusive as to whether there is a stress-induced gene expression effect or not and it may be confusing to keep those points in the text.

**Response:** Thanks for the suggestion. In this revision, we have removed those points from the text. Also, we moved the assessment of stress-induced transcriptomic effects to the **Methods** and **Discussion** in this revision.

Ok, thank you.

7) The accuracy of cell type annotations throughout the manuscript is unclear.

***Response(R1):*** *Thanks for the comment. First, we handled our data and annotated cell* *types with analytic software and pipeline that have been applied in multiple studies (The Tabula Muris Consortium et al., Nature 2018, 562:367-372; Han et al., Nature 2020:581, 303-309). Second, we annotated all cell clusters with marker genes for each cell type, which are well-known or have been published previously (please kindly check the gene list and corresponding references in the Additional file 2: Table S3 in this revision). Specifically, for a subtype of major cell type, we named it “major cell XXX_high” based on the most specifically highly expressed marker gene XXX in the cluster in Additional file 2: Table S4-S19 in this revision (Table S3 in the previous version). Third, the paired TCR and BCR information confirmed the accuracy of T cell and B cell annotations, respectively, and the developmental trajectory coupled with TCR/BCR information further confirmed the accuracy of annotation for T/B cell subtypes. Fourth, our trajectory analyses revealed developmental relationship for immune cells, such as monocyte-macrophage, that are consistent with the previous understanding but at a higher single-cell resolution. Fifth, we observed that our findings are consistent with a very recent study in human organ samples (Han et al., Nature 2020:581, 303-309), in terms of the gene expression profiles of B cells and epithelial cells and the transcriptional factor activity between both digestive and non-digested tissues. Sixth, we identified multiple TFs for the regulation of cell development between cell types, of which some are well-known. For example, SPI1 are involved in the development of monocytes to macrophages. Moreover, some TFs for human B cells and plasma cells could be consistently found in a very recent study (Han et al. Nature 2020:581, 303-309). Lastly, as suggested by the Reviewer #2, we have also validated our discovery of novel/rare cell types using immunofluorescence staining assays. Taken together, these strongly suggest that the analytic procedures and the cell type annotations in our study are robust.*

See response below in point 8.

**Response:** Thanks.

8) The expression of marker genes should be shown in a heatmap or dot plot in the main figure.

***Response(R1):*** *Thanks for the suggestion. As suggested, we added t-SNE plots to show the expression of marker genes for major cell types in the main figure (Fig. 1C).*

This comment goes together with the one above (point 7) and the original suggestion was to show markers for each identified population in the form of a heatmap or dot plot. The authors have opted for showing feature plots of a subset of markers for some annotations, however, for other annotations it remains unclear which markers are they based on, e.g. which marker were used to distinguish central memory from naïve T cells or effectors from resident memory? Showing the expression of canonical marker genes for known populations in a transparent way is required. Showing the expression of marker genes for all populations would be a more complete and transparent way to present the data.

**Response:** Thanks for the suggestions. As suggested, we have provided marker genes and their expression for each cell types in this revision, with dot plots or violin plots. Please kindly check at main **Figure 1C**, **Figure 2C** and **D**. For example, in the manuscript **Figure 1** (**B** and **C**), reproduced as below **Response Figure 9**, we presented a dot plot showing the expression of a subset of markers for major cell types (**Response Figure 9B**).

We also added violin plots to show the canonical markers of CD4^+^ and CD8^+^ cells (Zhang *et al., Nature* 2018, 564:268-272; **Response Figures 10** and **11**). We note that *CD4* gene was only significantly detectable in several CD4^+^ T cell clusters (**Response Figure 10B**), which might be due to the low presentation of 10x Genomics technology, while *CD8A* and *CD8B* were broadly expressed in all CD8^+^ T cell clusters (**Response Figure 11B**). We were not able to distinguish T_N_ from T_CM_ using CD45RA^+^CD45RO^-^CCR7^+^ and CD45RA^-^CD45RO^+^CCR7^+^ for the two cell types, respectively (Schmueck-Henneresse *et al., J Immunol* 2015,194(11):5559-67), since our data generated using 10x Genomics is not feasible for the determination of different *CD45* isoforms. Therefore, in our study, we used *CCR7*, *SELL*, *TCF7*, and *LEF1* as markers for T_N_ (naive T cell) and T_CM_ (central memory T cell) according to previous literatures (Hashimoto *et al*., *Proc Natl Acad Sci USA* 2019, 116:24242-24251). A cell cluster with high expression of both *CCR7* and *SELL* (lnFC greater than 0.7 for CD4^+^ and 1.0 for CD8^+^) in the top 10 rankings of DEG was assigned as T_N_ cells*.* Otherwise, clusters with expression of *CCR7* and *SELL* were assigned as T_CM_ cells.

**Response Figure 9**. Cells distribution across different organs and the marker genes of each cluster.

**A**. t-SNE showed the distribution of cells from different tissues coloured according to their origins of organ. Each dot represents a cell. The labelled cell types were the predominant cell types in each cluster.

**B**. Dot plots showed the most highly expressed marker genes (x axis) of major cell types (y axis) in **A**. The depth of colours from white to blue and the size of dot represent the average expression from low to high and the percentages of cells expressing the gene, respectively.

We observed different expression of marker genes between effector T cells (T_EFF_) and resident memory T cells (T_RM_; **Response Figure 11**). *FGFBP2* and *TBX21* were common markers for CD4^+^ and CD8^+^ T_EFF_ cells*,* with additional specific markers *GNLY* and *FCGR3A* for CD4^+^ and CD8^+^ T_EFF_ cells, respectively; *MYADM*, *NR4A1*, *CXCR6*, and *ITGAE* were markers for CD4^+^ T_RM_ cells; and *ITGAE*, *XCL1*, and *XCL2* were markers for CD8*^+^* T_RM_ cells (**Response Figure 11**). Moreover, T_EFF_ from T_RM_ cells also showed distinct expression signatures (**Response Figure 12**). Only T_EFF_ cells (NKG7_T_h1_, TRBV4-2_T_EFF_, MT1E_T_EFF_, and GNLY_T_EFF_) exhibited simultaneously strong expression of effector or cytotoxic genes including *GZMA*, *GZMB*, *PRF1*, *GZMH*, and *NKG7* (Zhang *et al., Nature* 2018, 564:268-272; **Response Figure 12**). In addition, we didn’t use *CD69* in combination of *ITGAE* to classify T_RM_, because *CD69* was detected widely in both CD4^+^ and CD8^+^ T clusters.

In this revision, we have also added the above results in the manuscript. Please check at the main **Figure 2C** and **D**.

**Response Figure 10**. Cell clusters and expression of canonical markers for each CD4^+^ T cells.

**A**. t-SNE plot of 7,006 CD4^+^ T from 15 organ tissues. Each point represents a cell, coloured according to their origins of organ (top panel) or cluster (bottom panel) as indicated on the right legend.

**B**. Violin plots showed the normalized expression of marker genes for each CD4^+^ T cell cluster as indicated at the bottom. For each panel, y-axis shows the normalized expression level for a marker gene as indicated at the left. Marker genes were also grouped according to functional cell types.

**Response Figure 11**. Cell clusters and expression of canonical markers for each CD8^+^ T cells.

**A**. t-SNE plot of 11,286 CD8^+^ T from 15 organ tissues. Each point represented a cell, coloured according to their origins of organ (top panel) and cluster (bottom panel) as indicated on the right legend.

**B**. Violin plots showed the normalized expression of marker genes for each CD8^+^ T cell cluster as indicated at the bottom. For each panel, y-axis shows the normalized expression level for a marker gene as indicated at the left. Marker genes were also grouped according to functional cell types.

**Response Figure 12**. Normalized expression of effector molecules in CD4^+^ and CD8^+^ T cell clusters.

**A. B**. Violin plots showed the normalized expression of effector molecules for each CD4^+^ and CD8^+^ T cell cluster as indicated at the bottom. For each panel, y-axis shows the normalized expression level for an effector molecule as indicated at the left.

We thank the authors for adding these plots for marker genes. CCR7 and SELL are markers for both naïve and central memory T cells and this is how they are reported in the cited paper (Hashimoto et al., Proc Natl Acad Sci USA 2019, 116:24242-24251). We would therefore suggest the reannotation of these cells as naïve/central memory T cells as the difference between the two subtypes cannot be reliably established.

**Response:** Thanks for the thoughtful suggestion. Although both naïve and central memory T cells express *CCR7* and *SELL*, we noted higher expression of *CCR7* and *SELL* in naïve T (T_N_) cells compared to central memory T (T_CM_) cells. Please kindly refer to the Figure 3B in the original paper by Cano-Gamez *et al.* (*Nat Commun* 2020, 11:1801), which was reproduced as below **Additional** **Response Figure 2** for a better reference. In our study, a high expression of both *CCR7* and *SELL* (lnFC greater than 0.7 for CD4^+^ and 1.0 for CD8^+^) among the top 10 rankings of DEG in each cluster was used as criteria to distinguish T_N_ from T_CM_ cells. Indeed, we observed the initial state of differentiation at the starting root for T_N_ cells in the trajectory analyses (LINC00861_T_N_ and SELL_T_N_ for CD4^+^ and CD8^+^ T cells, respectively; **Response Figures 13** and **14**). We also observed intermediate state and terminal state for KLF2_T_CM_ and STMN1_T_CM_, respectively, in the developmental trajectories of CD4^+^ cells (**Response Figure 13**). However, for the other cell clusters, we had less evidence to group them accurately as either T_N_ or T_CM_. Therefore, we reannotated these cells as T_N_/T_CM_ according to the Reviewer’s suggestion. We have updated the relevant content in the revised manuscript, figures, tables, and supplementary materials. Please kindly check at the lines 5, 8-10 in the 2^nd^ paragraph, Page 7, and **Figure 2C** and **D** in the main text; **Supplementary Table S22-S27** and **Supplementary Figure 13** and **14**.

**Additional Response Figure 2.** Marker genes of each CD4^+^ T cell cluster. Colours encode the mean expression of each gene in each cluster. *For the purpose of quick reference, the figure was reproduced from the Figure 3B in the original paper by Cano-Gamez *et al*.

9) Automated doublet detection should be compared to manual exclusion of doublets. What do authors refer to as "unassigned" cell types (e.g. "Clusters due to cell doublets or unassigned cell types were identified and excluded manually <..>")?

***Response(R1):*** *Thanks for the comments. Initially, we had applied DoubletFinder to our data. We observed that only a few cells were inferred as doublets and some proportion of cells were still self-clustered and deviated from other cells, meaning that these are likely doublets overlooked by the automated software. Therefore, we applied a more stringent method, which was based on the similarity of the markers and gene expression profiles of cell types to determine doublets manually. As suggested, we compared two methods for the removal of doublets, namely the automated detection and manual exclusion, using muscle and heart as examples. We found that 15.76% and 10.8% of cells were identified as doublets manually in muscle and heart samples, respectively. By contrast, 5% of cells were inferred as doublets for both samples using the automated DoubletFinder. Moreover, the doublets identified manually cover 47.06% (216 out of 459) and 65.73% (234 out of 356) doublets identified by DoubletFinder in the muscle and heart tissues, respectively. In order to minimize the potential impact of these doublets on the downstream analyses, we have applied the manual method as a more conservative way to remove doublets in our dataset. For more detailed comparison results including figures, please kindly refer to our response to the comment raised by the Reviewer #1 (point 3 in Response(R1) Page 9-13). In this revision, we have added above descriptions in Supplementary Methods (Additional file 30).*

*We defined doublets as any cell with 1) expression of multiple markers of different cell types, 2) a gene expression profile overlapping with multiple cell types, and 3) more than twice median numbers of UMIs and genes compared to major cell types within each organ, which were removed manually. The “unassigned cells” were those with less confidence to define as doublets, meeting only one or two of the above three criteria. The “unassigned cells” formed clusters in the bladder, lymph node, muscle, skin and the merged data, independently. DoubletFinder could not identify these cells as normal or doublets. Therefore, we applied a conservative way by defining both mixed cells and "unassigned cells” as doublets and removing them from subsequent analyses. In this revision, we have updated the definition in the METHODS section. Please kindly check in the last sentence in Paragraph 1, Page 26.*

The authors of DoubletFinder recommend users to threshold doublet calling by calculating the mean-variance-normalized bimodality coefficient of pANN distributions produced during pN-pK parameter sweeps of the data. BCMVN can be used to identify the pK that separates singlets and doublets effectively. Have the authors optimized the threshold for this specific dataset? This may be contributing to the low doublet detection by DoubletFinder.

The authors mention that “the doublets identified manually cover 47.06% (216 out of 459) and 65.73% (234 out of 356) doublets identified by DoubletFinder in the muscle and heart tissues”. Since there is limited overlap between the two methods, the authors may want to combine the doublets detected by DoubletFinder with the manually detected ones. Correct “comparation” for “comparison”.

**Response:** Thanks for the suggestions. As suggested, we combined both “DoubletFinder” and then manual detection methods to identify and remove the doublets in each organ. For “DoubletFinder”, we set an estimated proportion of doublets as 5% and an optimized pK using BCMVN for each organ (**Response Table 2**). We then manually removed the potential doublets clusters in each organ as we mentioned in the previous submission. After that, we rerun all the downstream analyses and updated the results, related figures and tables in this revision. Compared to the previous submission, additional 4,275 ‘cells’ were determined as doublets and removed from the analyses, resulting in a total of 84,363 cells. We note that the removal of new doublets had minimal impact on our findings. In this revision, we have updated the methods in the **Methods** section and the relevant results in the main text. Please kindly check at the 2^nd^ paragraph, Page 27 to the 1^st^ paragraph, Page 28; and **Supplementary Table 51**. As such, we also removed the comparison of ‘DoubletFinder’ and the manual detection methods described in the previous supplementary methods. In addition, we corrected the typo ‘comparation’ to ‘comparison’. We sincerely apologized for this overlook.

**Response Table 2**. Optimal pK values for each organ.

| **Tissue** | **Optimal pK** |
| --- | --- |
| Bladder | 0.005 |
| Blood | 0.02 |
| Common bile duct | 0.29 |
| Esophagus | 0.005 |
| Heart | 0.005 |
| Liver | 0.005 |
| Lymph node | 0.005 |
| Marrow | 0.02 |
| Muscle | 0.01 |
| Rectum | 0.005 |
| Skin | 0.28 |
| Small intestine | 0.005 |
| Spleen | 0.01 |
| Stomach | 0.01 |
| Trachea | 0.005 |

Ok, thank you.

10) The pseudotime analysis of T cells and myeloid cells reflects transcriptional similarities. The authors overinterpret the connection of these trajectories to the developmental stage of cells, provide no new insights or lack of experimental validation or further supporting data. The analysis of gene expression dynamics along pseudotime could add value to this analysis.

***Response(R1):*** *Thanks for the suggestion. Initially, we had tried to analyze gene expression dynamics along pseudotime using monocle 2. We observed multiple branches and nodes in the trajectory trees for T cells and myeloid cells, however, we did not observe a clear dynamic change of genes related to the development. These might be explained by that monocle 2 could not handle cells with too many subtypes (Response(R1) Figure 10 A-C). The new version of monocle (monocle 3 alpha and 3) could handle more complicated trajectory analysis with more different developmental states of cells (http://cole-trapnell-lab.github.io/monocle-release/monocle3/). However, too many developmental nodes identified by monocle3 alpha make it hard to resolve gene expression dynamics along pseudotime like in monocle 2 with limited nodes.*

*Alternatively, we presented the expression of genes that are known to regulate the corresponding cells in the pseudotime plots as in Fig. 2C, D and Additional file 10: Figure S7G-H in this revision. In general, we observed consistency between the development of cells and the expression of marker genes regulating the developmental process. For examples, TCF7 and LEF1 are Wnt pathway effectors and were reportedly expressed at the highest levels in naïve and central memory cells and lower levels in transitional and effector memory cells (Billingsley et al., PLoS Pathog 2015, 11:e1004740). We observed high activity and expression of TCF7 and LEF1 in CD4^+^, CD8^+^ T_N_, and T_CM_ cell clusters (Fig. 2D, E, and Figure S7G, H). FOXP3 is a specific marker of Treg cells and necessary for their suppressive activity on T cells (Hori et al., Science 2003, 299:1057-1061). We observed that FOXP3 was only enriched in CD4^+^ Treg cluster in the pseudotime plot and had higher activity in this cluster (Fig. 2D). Similarly, we observed that SPI1 had higher activity in monocytes and had higher expression level in corresponding monocyte clusters (Fig. 4F, G).*

*We have updated the results in the main text. Please kindly check in the RESULTS section in Paragraph 2, Page 8; Additional file 10: Figure S7G, H; Paragraph 2, Page 13 and Fig. 4G.*

*Response(R1) Figure 10. Pseudo-time of CD4^+^, CD8^+^ T cells, and monocytes/macrophages*

*A. Pseudo-time of CD4^+^ inferred by Monocle2 (left panel). Each point corresponds to a single cell. Clusters information was shown at the right panel. Selected genes expression along the psedutime were shown in the right panel. TCF7 and LEF1 for T_CM_ and T_N_, STAT1 and IRF1 for T_EFF_, FOXP3 for T_reg_, and FOS for T_RM_.*

*B. Pseudo-time of CD8^+^ inferred by Monocle2 (left panel). Each point corresponds to a single cell. Clusters information was shown at the right panel. Selected genes expression along the psedutime were shown in the right panel. TCF7 and LEF1 for T_CM_ and T_N_, STAT1 and TBX21 for T_EFF_, and FOS and ATF3 for T_RM_.*

*C. Pseudo-time of monocytes and macrophages, inferred by Monocle2 (left panel). Each point corresponds to a single cell. Clusters information was shown at the right panel. Selected genes expression along the psedutime were shown in the right panel. RXRN and ELF2 for classical monocytes, TCF7L2 and STAT1 for non-classical monocytes and LIPA_macrophage, and JUN and PRDM1 for most of non-classical monocyte clusters.*

Validating known markers, such as FOXP3 in Tregs, is not the purpose of trajectory analysis. As it stands now, this trajectory analysis does not lead to new biological insights and it may lead to confusion. Regulatory T cells (8) in Fig. 2C are standing next to Tcm(3). Do the authors think this makes biological sense? What is their explanation for this? Also, the central memory CD4 T cells in cluster (1) are at the end of the trajectory. What is the authors’ hypothesis regarding this feature of their trajectory?

**Response:** Thanks for the inspirational questions and suggestions. As pointed out by the Reviewer, our trajectory analysis revealed the branching out of CTLA4_T_reg_ (previous Treg (8)) cells next to KLF2_T_CM_ (previous Tcm (3)) cells along the development trajectory from T_N_ cells to T_RM_ cells (**Response Figure 13**). We note a similar observation reported most recently in T cells (Cano-Gamez *et al., Nat Commun* 2020, 11:1801), where human naïve T cells developed towards central memory, then effector and finally highly effector memory T cells, with natural Treg cells branching out from central memory T cells. Please kindly refer to the Figure 3C in the original paper by Cano-Gamez *et a.*, which was reproduced as below **Response Figure 14** for a better reference. These observations might suggest the conversion/differentiation potential from central memory T cells to Treg cells, which has been demonstrated previously using *in vitro* models with TGF-β stimulation (Zhang *et al., PLoS One* 2013, 8(10):e77322).

**Response Figure 13**. Pseudo-time trajectory analysis of CD4^+^ T cells with high variable genes. Each dot represents a single cell and is coloured according to their clustering in the right legend. The inlet t-SNE plot shows each cell with a pseudo-time score from dark blue to yellow, indicating early and terminal states, respectively.

**Response Figure 14**. Branched pseudotime trajectory, each cell is coloured by its pseudotime value (left panel) or its cluster label (right panel). *For the purpose of quick reference, the figure was reproduced from Figure 3C in the original paper by Cano-Gamez *et al*.

Also, as pointed out by the Reviewer, a clear central memory T cell cluster (STMN1_T_CM_, previous Tcm (1)) is at the end of the trajectory, likely differentiated from tissue resident memory T cells (TNF_T_RM_, previous T_RM_ (2)) according to the pseudotime scores. Together with that STMN1_T_CM_ cells were collected from lymph nodes, this observation provides a clue to solve the puzzle whether tissue-homing memory T cells further differentiate or can migrate back to the lymphoid compartment (Lees *et al., Immunology*. 2010, 130(4): 463-470). Supportively, a very recent study using mice model demonstrated that T_RM_ cells in the skin could differentiate into T_CM_ and T_EM_ cells upon local reactivation, which then rejoined the circulation (Fonseca R *et al., Nat Immunol* 2020, 21:412-421). Taken together, these observations suggest that T_RM_ cells have developmental plasticity rather than representing a terminal stage of differentiation.

Compared to other T_CM_ clusters, STMN1_T_CM_ was at terminal development stage expressing mediate level of *FOS*/*JUN* and high level of *CTLA4*, an active marker of CD4^+^ T cells. To further investigate the development of the STMN1_T_CM_ cells, we performed trajectory analysis with TNF_T_RM_ and STMN1_T_CM_ clusters, which revealed that TNF_T_RM_ from two branches with early state gradually progressed towards STMN1_T_CM_ with terminal state (**Response Figure 15**). T_RM_ cells at the beginning of the trajectory expressed high levels of T_RM_ markers, such as *RUNX3*, *NR4A1*, chemokines (*CCL5*), and other T_RM_ associated genes, including *ID2*. By contrast, T_CM_ cells at the end of the trajectory was marked with expression of well-known T_CM_ molecules, such as *SELL* and *CCR7.* In addition, some other genes that are poorly characterized were also enriched in T_CM_ cluster, including genes encoding ribosomal proteins and *TXNIP*.

In this revision, we have added the above information in the **Discussion** section. Please check at the last three sentences in the last paragraph, Page 20; the last three sentences in the 1^st^ paragraph, Page 21.

The cited study (Cano-Gamez et al., Nat Commun 2020, 11:1801) reports data on a highly controlled system where naïve and memory T cells are polarized *in vitro* towards four helper phenotypes (Th1, Th2, Th17 and iTreg) as well as the effectorness gradient in resting CD4+ T cells derived from peripheral blood. The authors are careful not to claim that these cells are differentiating from central memory T cells, as this cannot be concluded based on this analysis. Their trajectory analysis is interpreted in terms of effectorness. We would therefore advice the authors to adjust their claims. Differentiation of Tregs from central memory T cells is possible, however, whether this is the case *in vivo* remains to be established with precise lineage tracing methods.

**Response:** Thanks for the thoughtful comment and suggestion. As suggested, we have adjusted the claims in our manuscript by not to overstate the potential differentiation of central memory T cells to Tregs. Please kindly check at the **Discussion** section, the last three sentences in the 1^st^ paragraph, Page 21, as read “In addition, we observed the branching out of CTLA4_T_reg_ cells next to KLF2_T_CM_ cells along the development trajectory from T_N_ cells to T_RM_ cells (**Figure 2E**). Given that previous *in vitro* studies have demonstrated a potential development from central memory T cells to Treg cells upon stimulation. This observation suggested that differentiation of Tregs from central memory T cells is possible, however, whether it holds true in vivo remains to be established with precise lineage tracing methods.”

The authors should note that Tgd are not CD8 T cells and MAIT are thought to develop via a different route in the thymus. Please, correct the data accordingly.

The authors should also take into account that gdT are not CD8 T cells, Tcm are CCR7+ and MAIT are thought to develop via a different route.

**Response:** Thanks for the suggestions. As suggested, we had excluded the γδ T cells in the previous revision. We further excluded MAIT cells from the trajectory analysis in this revision, which revealed similar differentiation trajectories for CD8^+^ T cells compared to that in previous submission. We observed that the trajectory tree rooted from T_N_ cells and sprouted out towards T_N/CM_, T_EM_, and T_RM_ branches for CD8^+^ T cell (**Response Figure 16**). T_EFF_, T_RM_, and IEL CD8^+^ T cells showed higher pseudo-time scores, suggesting their terminal development state (**Response Figure 16**).

In this revision, we have updated the above results accordingly. In addition, we used *CCR7*, *SELL*, *TCF7*, and *LEF1* as markers for naïve/central memory T cells (T_N/CM_). Please kindly refer to our response to the Reviewer’s point 8 above.

**Response Figure 15**. Pseudotime trajectory analysis and gene expression profiles of TNF_T_RM_ and STMN1_T_CM_.

**A**. Branched pseudotime trajectory with TNF_T_RM_ and STMN1_T_CM_. Each cell as dot is coloured according to a cluster label (left panel) or pseudotime value (right panel).

**B**. Expression heatmap of selected variable genes along the pseudotime trajectory (from Monocle). Each row represents a gene as indicated at the right; column bars represent cells ordered by pseudotime (from left to right) and different colours correspond to the scaled (Z-scored) expression of each gene in each cell.

**Response Figure 16**. Pseudo-time trajectory analysis of CD8^+^ T cells with high variable genes. Each dot represents a single cell and is coloured according to their clustering in the right legend. The inlet t-SNE plot showed each cell with a pseudo-time score from dark blue to yellow, indicating early and terminal states, respectively.

Ok, thank you.

Could the HSPA1A CD8+ population be a stress-induced artefact? Similar question for the Trm populations expressing FOS/JUN. How could the authors support the idea that these are not stress-induced genes?

**Response:** Thanks. We don’t think that *HSPA1A* CD8^+^ T population and the T_RM_ populations expressing *FOS*/*JUN* are stress-induced artefacts, although the upregulation of *HSPA1A*, *FOS*, and *JUN* has been reported in certain scenarios with stress stimulations such as enzyme digestion during dissociation process (van den Brink *et al., Nat Methods* 2017, 14:935-936).

First, we had provided lines of evidence to show that dissociation processing in our study might have minimal impacts on the expression of *HSPA1A*, *FOS*, and *JUN* as well as the transcriptome of majority of cells in our dataset. Please kindly refer to our previous ***Response(R1)*** to the Reviewer’s point 12 below. The Reviewer commented that “The assessment of stress-induced transcriptomic effects is valuable and the fact that the identified clusters are not tissue-specific or dissociation-dependent is convincing.” Please kindly refer to the **Reviewer’s comment at point 5** above.

Second, the majority of *HSPA1A* CD8^+^ T cells (86.2%; 1,072 out of 1,244) were detected in the common bile duct, showing a tissue-specific distribution as reported previously in a mouse study (Beura *et al.,* *Immunity* 2018, 48:327-338 e325). The study demonstrated that *Hspa1a* was only enriched in the female reproductive tract (FRT) CD8^+^ T_RM_, but not in the spleen T_RM_ and T_CM_ cells. Moreover, although similar dissociation conditions were applied to multiple organs (**Response** **Table 1**), high expression of *HSPA1A* was observed specifically in HSPA1A_T_RM_ and a few other cell clusters (**Response Figure 17**). Supportively, exclusively high expression of *Hspa1a* was observed in only one of two T follicular helper (TFH) like cell clusters but no other T cells types in the lung of mouse (Swarnalekha *et al., 2020 BioRxiv;* https://doi.org/10.1101/2020.02.28.963280). These observations suggest *HSPA1A* to be one of the specific markers for T cell subtypes.

Third, we suspected that high expression of *FOS* and *JUN* in CD8^+^ T_RM_ populations (**Response Figure 17**) might reflect their role in maintaining the cells rather than in response to the dissociation-related stress. *FOS* and *JUN* are members of AP-1 transcription factor, of which dimerization has been shown to play numerous roles in a wide range of cellular process, cell growth, and proliferation, etc. We did observe the upregulation and the high regulon’s activity of many AP-1 dimerization partners (such as *FOS*, *JUN*, *JUNB*, *FOSL2*, and et) for CD4^+^ (**Response Figure 18A** and **B**) and CD8^+^ T_RM_ cells (**Response Figure 17**, **Response Figure 18C**) in our dataset. It has been reported recently that AP-1 dimerization partners such as *Fosl2* and *Junb* were highly expressed in T_RM_ cells, and the knockdown of *Junb* resulted in impaired T_RM_ cell differentiation, confirming the key role of AP-1 dimerization regulating T_RM_ differentiation (Kurd *et at., 2020* BioRxiv; <https://doi.org/10.1101/2020.03.02.973578>). Given that *Fosl2* is able to regulate *Smad3* (Ciofani *et al., Cell* 2012, 151:289-303), a key component of the TGF*β* signalling pathway, *Fosl2* may also promote T_RM_ cell differentiation through effects on TGF*β* signalling. We note that high expression of *FOS* and *JUN* was only observed in most of T_RM_ cells, but not non-T_RM_ cells (**Response Figure 17, Response Figure 18A**) from the same organs that were undergone dissociation treatments with similar conditions. This observation, together with our previous observation that most organs had higher density of low total dissociation score (***Response(R1) Figure 11***), suggests that dissociation-related genes were not significantly induced in the majority of cells. Please also kindly refer to our previous response (***Response(R1))*** to the Reviewer’s point 12 below.

Lastly and importantly, we performed additional immunofluorescence assay with double staining of CD8A and FOS in multiple organs including common bile duct, liver, and small intestine from multiple donors, and confirmed the high expression of FOS in the CD8 cells before tissue dissociation (**Response Figure 19**).

In summary, these observations suggest that *HSPA1A* CD8^+^ T population and the T_RM_ populations expressing *FOS*/*JUN* are unlikely stress-induced artefacts. We have updated the data in the **Discussion** section in this revision. Please kindly check at the last sentence on Page 23 to the 1^st^ sentence on Page 24, and **Additional file 7 Supplementary Notes**.

**Response Figure 17.** The violin plots showed the normalized expression of *FOS*, *JUN*, and *HSPA1A* in CD8^+^ T cell clusters.

**Response Figure 18**. Gene expression and cluster-specific regulon activity of T cells.

**A**. Normalized expression of *FOS* and *JUN* in different CD4^+^ T cell clusters.

**B, C**. Heatmap of the active scores of T cell subtypes (**B** for CD4^+^ T, **C** for CD8^+^ T cells, respectively), as estimated using SCENIC analysis of transcription factors (TFs). Shown on the right are the top 10 TFs with the highest difference in the expression regulation estimates between each cluster (indicated on top) and all other cells, tested with a Wilcoxon rank-sum test. Filled colours from blue to red represent scaled TF activities from low to high.

**Response Figure 19**. Representative images of immunofluorescence staining of FOS and CD8A in small intestine, common bile duct, and liver tissues. Markers used are indicated on top. Samples were derived from donor as indicated at the left. Red arrows indicated the co-expression of proteins FOS and CD8A.

Ok, thank you.

12) The transcription factor signature in T cells that comprised FOS, FOSB, JUN, JUND, etc is concerning since these have been previously shown to be upregulated in response to tissue dissociation-induced stress (Brink et al, 2017 Nature Methods). The authors should explain how they can exclude this is not an artifact of the tissue processing.

***Response(R1):*** *Thanks for the suggestion. We fully understand the Reviewer’s concern about the potential effect of dissociation-induced stress on cells, which should be minimal on overall transcriptome. First, we evaluated the effect of tissue processing on cells in our data, using a method described previously (the Tabula Muris consortium et al., Nature 2018, 562:367-372). We observed that most organs had higher density of low total dissociation score, meaning that dissociation-related genes were not significantly induced in the majority of cells (Response(R1) Figure 11). Second, the typical dissociation-related satellite-cell genes (van den Brink SC et al., Nat Methods 2017, 14:935-936), such FOS, HSPA1A and SOCS3, were not consistently expressed in all cell types in muscle (Response(R1) Figure 12). HSPA1A had lower expression level in both two satellite-cell clusters than other clusters. SOCS3 only slightly expressed in one of two satellite-cell clusters. Third, dissociation-related genes showed different expression pattern in a same organ where all cells were treated with a same dissociation procedure (Response(R1) Figure 13). For example, in the small intestine and stomach, FOS, JUN, and JUNB broadly expressed in different cell clusters with various degree, while SOCS3 and HSPA1A only specifically highly expressed in adoptive cell, macrophage in the small intestine, and enriched in monocyte, fibroblast, and chief cell in the stomach. Fourth, we observed that same cell types from different organs treated with different dissociation procedures had similar expression pattern of the dissociation-related genes. Taking T, B, and plasma cells in the small intestine and stomach for example, each of them had similar expression pattern of those dissociation-related genes. Moreover, each major cell type from different organs was clustered together in t-SNE plot, suggesting similar transcription profiles in spite of different dissociation treatments (Fig. 1B).*

*Taken together, the above observations suggest that dissociation processing in our study might have a minimal impact on the transcriptome of majority of cells in our dataset. Nevertheless, we could not rule out the possibility that the dissociation procedures might have impact on some certain cell types, which await further investigations. Please kindly check at Paragraph 2, Page 22 in the DISSCUSSION section. We also provided detailed information for the dissociation procedures and the evaluation of potential effects of dissociation-induced stress on the transcriptome as Supplementary Methods (Additional file 30).*

*Response(R1) Figure 11. Dissociation scores for each organ. A score represents the magnitude of the first principal component of principal component analysis based on the expression of 140 human dissociation-associated genes of each organ (van den Brink SC et al., Nat Methods 2017, 14:935-936). The y axis shows the probability density of the normalized histogram.*

*Response(R1) Figure 12. Violin plot showed the normalized expression of typical dissociation-related genes in the muscle sample. Shown were SOCS3, FOS, and HAPA1A1.*

*Response(R1) Figure 13. Violin plot showed the normalized expression of typical dissociation-related genes in the small intestine and stomach samples. Shown were FOS, FOSB, JUN, SOCS3, and HAPA1A.*

Response above in point 5.

**Response:** Thanks. Please kindly refer to point 5 above.

14) Authors use pseudotime trajectory inference on circulating monocytes and macrophages pooled from all tissues, however, they fail to take into account that most tissue-resident macrophages are not derived from circulating monocytes but from embryonic progenitors (e.g. self-renewing embryonic macrophages in the skin, liver or gut). As mentioned above, the consideration of the biological context is essential for a robust interpretation of trajectory analysis.

***Response(R1):*** *Thanks for the thoughtful comments. We fully agree with the Reviewer that most tissue-resident macrophages are derived from embryonic progenitors and undergo self-maintenance via local expansion in the steady human organ, with minimal contribution from circulating monocytes (Hashimoto et al., Immunity 2013, 38:792-804; Jakubzick et al., Immunity 2013, 39:599-610; Sieweke MH et al., Science 2013, 342:1242974).*

*In our study, we performed trajectory analysis to investigate the potential developmental relationship between monocytes and macrophages. The developmental trajectory path from classical monocytes to tissue-resident macrophages suggests that classical monocytes could be a protentional origin of adult tissue-resident macrophages (Fig. 4E). One the other hand, high expression of proliferation markers including MKI67, PCNA, and TYMS were exclusively detected in the C12 macrophages (including those in bladder, common bile duct, esophagus, heart, and rectum). Moreover, GSEA analysis also revealed that most of the macrophage clusters had higher enrichment score of G2M checkpoint and E2F targets pathways (Additional file 14: Figure S10E). These observations suggest local expansion potential of tissue-resident macrophages. Therefore, our findings suggest tissue-resident macrophages in steady-state organs might have two origins, one from classical monocytes and another from self-expansion. However, with present data, it is hard to tell that self-expansion alone or slightly together with circulating monocytes contributes to the development of tissue-resident macrophages. Further investigations using cell labeling (Chen et al., Nat Commun 2018, 9:5065) or samples from donor who received bone marrow transplantation (Thomas et al., Science 1976, 192:1016-1018) may help address the question.*

*In this revision, although our observations suggest potential development relationship between circulating monocytes and tissue-resident macrophages, we acknowledge that “however, further investigations are awaited to address whether self-expansion alone or slightly together with circulating monocytes contributes to the development of tissue-resident macrophages, considering that most tissue-resident macrophages have been demonstrated originating from embryonic progenitor cells (Hashimoto et al., Immunity 2013, 38:792-804; Jakubzick et al., Immunity 2013, 39:599-610).” Please check in Lines 10-14, paragraph 2, Page 20 in the DISCUSSION section.*

Although authors have softened the language and incorporated the possibility that tissue-macrophages may be derived from embryonic cycling macrophages, the authors could have incorporated some comparisons with published fetal data, such as gut macrophages from Gao et al., 2018 (doi: 10.1038/s41556-018-0105-4). At minimum, the authors could provide novel pathways that change in monocyte-macrophage differentiation trajectory.

Thanks for revising this part. Still, the claim should be tone down and the header of the section should exclude the “origin of macrophages” part given that this analysis cannot reliably determine the origin of these cells but simply infer transcriptional similarities among those cells.

**Response:** Thanks for the suggestions. As suggested, we obtained the raw data from Gao *et al*.’s study (*Nature Cell Biology* 2018, 20:721–734) and identified 50 embryonic macrophages in 121 immune cells from fetal large-intestine samples. We further performed trajectory analysis inclusive the embryonic macrophages and 828 cells of monocytes and macrophages from rectum sample in our AHCA data (**Response Figure 20A**). Pseudotime analysis revealed that both embryonic and adult macrophages were at terminal state (**Response Figure 20A**). Moreover, we observed a clear differentiation trajectory of the macrophages in adult rectum from intermediate monocytes (CCL20_Intermediate_Mon) to tissue macrophages. Interestingly, the embryonic macrophages (Embryo_Mac) were found alongside the differentiation trajectory from the intermediate monocyte (FN1_Intermediate_Mon) to the tissue-macrophages (HIST1H4C_Mac), suggesting that embryonic macrophage may contribute to the tissue-macrophage through local expansion. Consistently, high expression of proliferation marker gene *PCNA* were detected in both the terminal macrophages (**Response Figure 20B**).

In this revision, we have added the above comparisons in the **Results** and **Discussion** sections as well as the related methods in the **Methods** section. Please kindly check at the 1^st^ paragraph, Page 13; the 1^st^ paragraph, Page 22; the last sentence in the 2^nd^ paragraph, Page 29. In addition, we removed “origin of macrophages” from the header as suggested.

**Response Figure 20**. Development trajectory of monocytes and macrophages in the intestine tissues.

**A**. Pseudo-time trajectory analysis of myeloid cells from rectum tissue in our AHCA dataset and macrophages from embryo large intestine in Gao *et al*.’s data. Each dot represents a single cell and is coloured according to their cluster. Only cell clusters with large than 50 cells in AHCA were included. The inlet t-SNE plot shows each cell with a pseudo-time score from dark blue to yellow, indicating early and terminal states, respectively.

**B**. Violin plots showed the normalized expression of proliferation marker genes (*MKI67* and *PCAN*) in each myeloid cell cluster as indicated at the x axis in the combined data of AHCA and Gao *et al*.’s samples.

Ok, thank you.

15) Epithelial cell analysis is focused on biology that has been previously described. The authors could use this dataset to further understand the core-similarities between epithelial cell subsets (e.g. secretory cells) across tissues as well as the key differences.

***Response(R1):*** *Thanks for the suggestion. As suggested, we performed additional analyses to examine the similarities of epithelial cells across different organs. First, we compared TFs in epithelial cell clusters, which revealed similar activation of TFs in most non-digestive clusters, as well as in digestive clusters (Fig. 5G). This was further confirmed using a recent published dataset (Additional file 19: Figure S13B). Second, pathway analysis revealed that multiple metabolic pathways were enriched in digestive clusters other than non-digestive cluster, suggesting the enhanced metabolic activity of digestive epithelial cells (Additional file 18: Figure S12C, D). Lastly, we performed ligand-receptor analysis for epithelial cells across different organs, which revealed that epithelial cells had the most frequent intra-cell interactions among all cell types, suggesting that epithelial cells could interact with each other intensively to regulate their biology functions in each organ (Additional file 24: Figure S17C and Additional file 19: Figure S21).*

*In this revision, we added the new results in the main text. Please kindly check in in Line 4-6, Paragraph 2, Page 14; Line 8-11, Paragraph 3, Page 14; Line 3-5, Paragraph 1, Page 15; Line 6-11, Paragraph 2, Page 15; Line 5-9, Paragraph 1, Page 16; Line 9-11, Paragraph 1, Page 21; and Line 16-18, Paragraph 2, Page 21.*

The authors should clarify which tissues do they classify as digestive tissue and what are the implications of this observation.

The detection of a “humoral immune response” GO term in epithelial cells suggests that there is a contamination in these cells. One possibility could be that ambient mRNA derived from lysed plasma cells led to the detection of plasma cell transcripts in these epithelial cells. Please, revise this point.

**Response:** Thanks for the thoughtful suggestions. According to the presence of digestive-related functions, stomach, small intestine, rectum, liver, and common bile duct were classified as digestive tissue in our AHCA data (**Response Figure 21A**), as were colons (ascending colon, colon, sigmoid colon, transverse colon), small intestines (duodenum, Ileum, Jejunum), liver, gallbladder, pancreas, stomach, and rectum in the Han *et al.*’s HCL dataset (**Response Figure 21B**). By contrast, all other tissues with detectable epithelial cells were considered as non-digestive tissue. Although esophagus is a part of digestive tract, epithelial cells from the tissue shared similar expression profiles with that in skin and trachea in both the AHCA and HCL datasets, indicating similar function of epithelial cells in esophagus compared to skin and trachea, but not other digestive organs (**Response Figure 21A, B**).

First, we investigated the contribution of TFs in regulating the heterogenous transcriptional profiles of epithelial cells in and between organs. SCENIC analysis revealed that the regulation of TFs was similar among cell clusters derived from a same tissue but was very different among cell clusters between tissues in both the AHCA and HCL dataset (**Response Figure 22**). Interestingly, the digestion-associated epithelial clusters exhibited similar activation of TFs, as did the cell clusters belong to non-digestive tissues including the trachea, skin, and esophagus (**Response Figure 22A**). Similar activation of TFs was observed between our AHCA and the HCL datasets for digestive-associated tissues (expect for pancreas) and non-digestive tissues (except for lung; **Response Figure 22B**). Moreover, we identified 204 TFs in our AHCA dataset, which overlap about 60% (120) of all cluster-specific TFs of the HCL dataset. Second, GO analysis revealed that distinct biological functions of cells between digestive tissue and non-digestive tissue. Metabolic process, energy synthesis, and digestion pathways were commonly observed in digestive tissue (**Response Figure 23A**), while *regulation of endopeptidase activity* and *epidermis development* were the strongly enriched pathways observed in non-digestive tissue (**Response Figure 23B**). Third, GSVA analysis also revealed significantly different pathways between digestive and non-digestive cell clusters, including multiple metabolic pathways with elevated activity in digestive cells and *TNFA signal via NFKB* in non-digestive epithelial cells (**Response Figure 21 C** and **D**). These observations indicate the diverse molecular profiles underlying the functional heterogeneity between the two groups of tissues, as well as the similarity in gene profiles among different tissues of same function. We note that most of the digestion-related organs developed from endoderm, which might explain their similarity in gene profiles and functions (Zorn AM *et al., Annu Rev Cell Dev Biol* 2009, 25:221-251). In addition, we suspected that the epithelial cells of same digestive functions might also share similar response to pathogens or stimulus (Gunther *et al., Semin Immunopathol* 2018, 40:555-565).

As suggested, we have removed genes encoding immunoglobulins from the downstream analysis in this revision. We have also updated the above information in the **Results**, **Discussion,** and **Methods**, sections. Please kindly check at the lines 7-9 in the 3^rd^ paragraph, Page 14; the lines 14-17 in the 1^st^ paragraph, Page 15; the lines 10-12 in the 2^nd^ paragraph, Page 16; the last three sentences in the 2^nd^ paragraph, Page 22; the lines 2, 3 in the 1^st^ paragraph, Page 29

Thanks. Please correct above the list of colon samples: duodenum is not colon (marked in dark red above).

**Response**: Thanks for pointing out the typo. Duodenum is a part of the small intestine as described previously. We have corrected the typo in the response letter. We ensure that duodenum has been included correctly as a part of the small intestine in our manuscript.

**Response Figure 21**. Comparisons of gene profiles and functions of cells between digestive and non-digestive organs.

**A, B**. Phylogenetic tree of tissues in AHCA (**A**) or Han *et al.*’s HCL (**B**) dataset, based on their normalized mean expression values of epithelial cells (correlation distance metric, complete linkage). Only genes with ln(fold-change) above 0.25, *p.adjust* < 0.05 and pct.1 ≥ 0.2 in each organ were included in the analysis. Organs with less than 50 cells were excluded.

**C, D**. GSVA analysis showed the pathways (hallmark and curated metabolic pathways) with significantly different activation between non-digestive and digestive cell clusters in both the AHCA (**C**) and Han *et al.*’s HCL (**D**) dataset. Activation was determined using GSVA and comparison was tested with an R package limma.

**Response Figure 22**. Heatmap of scaled transcription factor activities.

**A, B**. Heatmap of the active scores for epithelial cell subtypes as numbered on top in our AHCA (**A**) and Han *et al.*’s HCL (**B**) datasets. Activation score was based on the expression regulated by transcription factors (TFs), as estimated using SCENIC analysis. Shown are the top 10 TFs having the highest difference in expression regulation estimates between each cluster and all other cells, tested with a Wilcoxon rank-sum test. TFs with similar activity across multiple digestive or non-digestive organ-derived clusters were outlined in green dashed boxes.

**Response Figure 23**. GO analysis results of each epithelial cell cluster.

**A, B**. Gene Ontology enrichment analysis results of each epithelial cell cluster in digestive organs (**A**) and non-digestive organs (**B**). Cell clusters were coloured according to their -log_10_(*P*-values). Only the top 20 significant terms (*p-value* < 0.05) were shown.

**Authors Response**

**Point-by-point responses to the reviewers’ comments:**

**Point-to-point responses to reviewers’ comments**

We appreciate the effort made by the authors and would now support the publication of this work. See below final suggestions in dark red.

**Response:** We are very appreciative of your support to the publication of our work as well as thoughtful suggestions. In this response letter, please kindly refer to below points for our responses (in blue) to the suggestions; and we have synchronized the name of cell cluster with that in the main text and updated the locations of the corresponding content in the revised manuscript. Also, we have addressed and incorporated all the comments and suggestions in the revised manuscript.

**Please note that all comments from the Reviewer #1 had been fully addressed and we only keep the comments from the Reviewer #2 yet to be fully addressed, together with the original comment from the Reviewer #2 (in black), our *previous Response(R1)* (in *black* and *Italic*) in the first revision, additional comments from the Reviewer #2 (in underlined and black), and our new response (in Blue), for better following-up.

**Reviewer #2:**

Summary:
He et al have compiled a single-cell RNA-seq and VDJ-seq dataset including 15 different organs from a single individual. The authors describe cell types and patterns of B and T cell clone distributions across organs. While many of the findings are expected, the authors make some interesting observations, however, the lack of replicates and experimental validation weakens any conclusion regarding novel and/or rare cell types.

***Response(R1)****: Thanks for the positive comment. In this revision, we have provided additional analyses and experimental validation for the identification of novel and/or rare cell types, which could be found below as to address the Reviewer’s comments.*

Overall, the authors have addressed many of the issues raised by the reviewers and the manuscript has improved significantly upon revision, however, there are still major concerns that preclude its publication following Genome Biology standards. Language and terminology should be carefully revised throughout the manuscript. See below for further details.

**Response:** Once again, we have been grateful to the Reviewer for thoughtful suggestions that helped improve our manuscript significantly. We fully understand that *Genome Biology* is one of the top journals in research areas of biology and biomedicine and we have been trying our best to present this work to a broader readership so that it would contribute useful information to the scientific community.

In this revision, we have improved further our manuscript with additional analyses and validation experiments according to the Reviewer’s suggestions. Moreover, we have carefully revised the manuscript, with language editing by a native English speaker and terminology confirmation/revision.

Ok, thank you.

Major comments:

2) This study has profiled only one donor. Given the high biological variation in humans, the applicability of these findings to the broader population are under question.

***Response(R1):*** *Thanks for the pertinent comment. We fully understand the Reviewer’s concern if our findings could be applied to broader population in the context of biological variations among individuals. In our study, thousands of cells from multiple organs were analyzed, which enables us to understand the heterogeneous composition of cells in human organs and the development links among cell types, and to identify important molecules and their networking. Such as, similar with the report by The Tabula Muris Consortium et al. (Nature 2018, 562:367-372), we observed close clustering of cells from different organs (more than seven organs) for major cell types, including T cells, B cells, NK cells, fibroblasts, endothelial cells, smooth muscle cells, macrophages, and monocytes. We also identified several well characterized TFs regulating the different cell development, including TCF7, SELE, MYC, and KLF2 for T_N_ cells, TBX21, STAT1, and IRF1 for T_EFF_ cells, and PRDM1, XBP1, FOS, and IRF4 for plasma cells. The consistencies between our findings and previous findings suggest that our study based on large scale transcriptomes at single cell level provides common biological understanding at higher resolution.*

*Moreover, we used an independent set of single cell sequencing data derived from human organs, which was published very recently (Han et al. Nature 2020:581, 303-309), and we managed to replicate our findings. First, we identified important transcription factors regulating plasma cell development, such as XBP1, MYC, etc., which have similar activation patterns in both datasets (Fig. 3E and Figure S9F). Second, epithelial cells from different organs showed strong heterogeneity, while epithelial cells within the same tissue had similar gene expression profile and clustered together, in both datasets (Fig. 5A and Figure S11B). Third, we found that both digestive and non-digested tissues showed similar transcriptional factor activities in both datasets (Fig. 5G and Additional file 19: Figure S13B). In addition, we note that we only selected B cells and epithelial cells for replication analyses, because the recent published data has low sequencing depth and extensive contamination between different cell types (Han et al., Nature 2020:581, 303-309).*

*Furthermore, we validated our discovery of novel and/rare cell types using experiment as suggested by the Reviewer #2, including sweat gland epithelial cell in skin, COCH^+^ fibroblast in the skin and heart, and FibSmo in the bladder, heart, and rectum in independent samples (Figure S3C and Figure S4A). Please check more details regarding this at below Reviewer’s comment point 4 (Response(R1) Page 38).*

*Taken together, although our data was derived from one donor, some important findings based on large number of cells and multiple organs might be generally applicable. However, we do acknowledge that further investigations with more individual samples will solidify our findings and discover more novel cell types, key molecules, and interaction network.*

*We have added the replication analyses and experimental results as well as limitation in this revision. Please kindly check in the RESULTS section in Paragraph 2, Page 6; Paragraph 1, Page 11; Line 4-7, Paragraph 2, Page 14; Line 8-13 Paragraph 3, Page 14; Line 3-6, Paragraph 1, Page 15; Line 6-12, Paragraph 2, Page 15; and Line 5-9, Paragraph 1, Page 16.*

The points mentioned above are based on well-established human cellular biology, e.g. XBP1 in plasma cell differentiation. This reviewer was rather referring to validating novel findings in either newly generated or existing datasets. The incorporation of the Han et al HCL dataset is a valuable addition, however, the fact that this had to be restricted to only 2 cell types is sub-optimal. There are other available datasets of higher quality that could be used for validation of the COCH+ fibroblasts (e.g. heart datasets: Tucker et al. 2020 Circulation; Litvinukova et al. 2020 biorxiv).

Please, indicate what does FibSmo stand for.

**Response:** Thanks for the suggestions. As suggested, we performed additional analyses using several existing public datasets and multiplex immunofluorescence staining in more human tissue samples to validate our novel findings.

First, we validated the presence of *COCH*^+^ fibroblasts in multiple skin and heart tissues (**Response Figure 1**), using two skin datasets (Solé-Boldo *et al., Communications Biology* 2020, 3:188 and He *et al., Journal of Allergy and Clinical Immunology* 2020, 145:1615-1628 and) and one heart dataset (Tucker *et at., Circulation* 2020) that are publicly available. Please note that we were not able to access the suggested dataset from Litvinukova *et al*. We observed a clear clustering of *COCH*^+^ fibroblasts with co-expression of *MMP2* and *COCH* genes in the skin dataset of Solé-Boldo *et al.*’s (cluster 30; **Response Figure 1A:** left top and **B:** left). A total of 144 *COCH*^+^ fibroblasts (out of 7,298 fibroblasts and 7,879 other cells in the skin) were identified in 5 skin samples in the dataset, with maximum 103 cells for one sample. We also observed a small group of *COCH*^+^ fibroblasts with high expression of both *COCH* and *MMP2* in the skin dataset of He *et al.*’s (cluster 18; **Response Figure 1A:** right and **B:** middle). A total of 125 *COCH*^+^ fibroblasts (out of 5,657 fibroblasts and 19,669 other cells in the skin) were identified in 14 skin samples in the dataset, with maximum 31 cells for one sample. By contrast, 454 *COCH*^+^ fibroblasts cells scattered among a total of 62,292 fibroblasts from the 26 heart samples in Tucker *et al*.’s dataset, with maximum 45 cells for one sample (clusters 1, 2, 4, 6, 7, 10; **Response Figure 1A:** left bottom and **B:** right). Noteworthily, we further validated the presence of *COCH*^+^ fibroblasts in more human tissue samples using multiplex immunofluorescence staining assays (Please kindly refer to Reviewer #2’s comment 4 below).

**Response Figure 1.** Expression of *COCH* and *MMP2* in cells from the skin and heart samples.

**A**. Violin plots showed the normalized expression of *COCH* and *MMP2* in cells of different clusters from the skin (top) and heart (left bottom) tissues retrieved from public datasets as indicated. Clusters were numbered at x-axis, and the expression level was shown in y-axis. Each dot represents a single cell.

**B**. Feature plots showed the normalized expression of *COCH* and *MMP2* in cells from skin (left and middle) and heart (right) tissues retrieved from public datasets as indicated at the bottom.

Next, we observed similar gene signatures of *COCH^+^* fibroblasts between our AHCA dataset and Solé-Boldo *et al.*’s dataset, compared to all other cells in respective dataset (**Response Figure 2**). 31 out of 50 upregulated genes in *COCH^+^* fibroblasts were shared between the two datasets, among which *COCH* and *ASPN* are the two most significant ones (**Response Figure 2**). Both *COCH* and *ASPN* are secretory proteins in the extracellular matrix and have been involved in extracellular matrix remodelling (Cho *et al., J Mol Med (Berl)* 2012, 90:1321-1331; Goel *et al., PLoS One* 2011, 6:e23070; Ikegawa, *Curr Med Chem* 2008, 15:724-728), suggesting the important role of *COCH^+^* fibroblasts in maintaining tissue homeostasis.

Second, we validated the presence of *SCGB2A2*^+^ sweat gland cells in multiple skin samples from the above two datasets (**Response Figure 3**). We observed a clear clustering of *SCGB2A2*^+^ sweat gland cells with co-expression of *DCD*, *SCGB2A2*, *PIP*, *KRT19*, and *MUCL1* genes in the skin dataset of He *et al.*’s (cluster 25; **Response Figure 3**: left panels). A total of 346 *SCGB2A2*^+^ sweat gland cells (out of 25,326 cells in the skin) were identified in 14 skin samples in the dataset, ranging from 5 to 102 cells for each individual sample. By contrast, we only observed one *SCGB2A2*^+^ sweat gland cell with co-expression of *DCD*, *SCGB2A2*, *PIP*, *KRT19*, and *MUCL1* genes in Solé-Boldo *et al*.’s dataset (cluster 28; **Response Figure 3:** right panels), as limited epithelial cells (ranging from 99 to 969) were collected from each donor in the study.

**Response Figure 2.** Gene signature of *COCH*^+^ fibroblasts in skin.

**A, B**. Scatter plots showed the differentially expressed genes compared to other cells in skin samples from our AHCA or Solé-Boldo *et al*.’s datasets. Selected genes were labelled if ln(fold-change) greater than 4 and 3 for upregulation (in red), or smaller than 0.25 and 0.33 for downregulation (blue) in the AHCA and Solé-Boldo *et al*.’s datasets, respectively.

**C.** Venn diagram showed the sharing of upregulated genes in *COCH^+^* fibroblasts in comparison to all other cells.

**Response Figure 3.** Expression of *DCD*, *SCGB2A2*, *PIP*, *KRT19* and *MUCL1* genes in cells from skin samples.

**A**. Violin plots showed the normalized expression of *DCD*, *SCGB2A2*, *PIP*, *KRT19*, and *MUCL1* in cells of different clusters from the skin tissues retrieved from public datasets as indicated. Clusters were numbered at x-axis, and the expression level was shown in y-axis. Each dot represents a single cell. The clusters for *SCGB2A2*^+^ sweat gland cells were boxed.

**B**. Feature plots showed the normalized expression of *DCD*, *SCGB2A2*, *PIP*, *KRT19*, and *MUCL1* in cells from skin tissues retrieved from public datasets as indicated at the bottom.

Third, we validated the presence of *MMP2*^+^*ACTA2*^+^ cells (assigned as FibSmo cells) in multiple bladder samples from two independent public datasets (Yu *et al., J Am Soc Nephrol* 2019, 30:2159-2176; Han *et al., Nature* 2020, 581:303-309; **Response Figure 4**). We observed a clear clustering of FibSmo cells with co-expression of *MMP2* and *ACTA2* genes in both datasets. A total of 1,447 FibSmo cells (out of 13,490 cells in the bladder) were identified in 3 bladder samples in the Yu *et al*.’s dataset, ranging from 42 to 709 cells for each individual sample (**Response Figure 4:** left panels). Similarly, a total of 178 FibSmo (out of 1,193 stromal cells in the bladder) were identified in 2 bladder samples in the Han *et al.*’s dataset, with 24 and 154 cells in each sample (**Response Figure 4:** right panels). Moreover, we confirmed the presence of FibSmo cells in multiple human samples using immunofluorescence staining assays. Please kindly refer to the Reviewer’s comment 4 below.

In summary, we confirmed the presence of *COCH*^+^ fibroblasts, *SCGB2A2*^+^ sweat gland cells, and FibSmo cells in multiple human tissue samples using additional existing datasets and immunofluorescence staining assays.

We named FibSmo cells as a novel cell type with co-expression of *MMP2* and *ACTA2*, which are marker genes for fibroblast and smooth muscle cells, respectively. Although FibSmo cells share marker genes with the two cell types, they had distinct gene signatures (**Response Figure 5A** and **B**). FibSmo cells exhibit high and specific expression of *PLAT* and *ID1,* which were related to clotting and angiogenesis (Carmeliet *et al., Nature* 1994*,* 368:419-424; Volpert *et al., Cancer Cell* 2002, 2:473-483), and higher expression of *COL3A1* and *COL1A1* compared to other stromal cells, which have been involved in wound healing (Crane *et al., Wound Repair Regen* 2010, 18:409-416). In addition, the enrichment of BMP signal genes *BMP4* and *BMP5* was also observed in the FibSmo cells (**Response Figure 5B**). GO analysis revealed that FibSmo cells shared common stromal functions with fibroblasts and smooth muscle cells, such as *extracellular structure organization* and *tissue remodelling*, while FibSmo cells had enhanced biological functions in *response to wounding* and *response to growth factor*, which is consistent with the functions of their highly expressed signature genes above (**Response Figure 5C**).

In this revision, we have added the above validation results and a more detailed description of FibSmo cells in the main text (please kindly check at the lines 8-10 and the last sentence in the 2^nd^ paragraph, Page 6; the lines 3-5, 16-26 in the 1^st^ paragraph, Page 18) and the Appendix (**Supplementary Figure S4-S10**, **Supplementary Figure 23**, and **Additional file 7 Supplementary Notes**). We also updated the related **Methods** (please kindly check at the 3^rd^ paragraph, Page 31 to the 2^nd^ paragraph, Page 32; the 1^st^ paragraph, Page 33).

**Response Figure 4**. Expression of marker genes in cells from bladder samples.

**A**. Violin plots showed the normalized expression of *MMP2*, *ACTA2*, *BMP4*, *BMP5*, and *PLAT* in cells of different clusters from the bladder tissues retrieved from public datasets as indicated. Clusters were numbered at x-axis, and the expression level was shown in y-axis. Each dot represents a single cell. The clusters for FibSmo cells were boxed.

**B**. Feature plots showed the normalized expression of *MMP2*, *ACTA2*, *BMP4*, *BMP5*, and *PLAT* in cells from skin tissues retrieved from public datasets as indicated at the bottom.

**Response Figure 5.** Gene expression and estimated functions of fibroblast, FibSmo, and smooth muscle cells.

**A**. Heatmap showed the average expression level of top 150 genes enriched in fibroblast, FibSmo, and smooth muscle cells as indicated in rows. Each column line represents one gene and the expression level from high to low was coded as red to blue, respectively.

**B**. Violin plots showed the expression of marker genes of FibSmo in the three cell types as indicated at the bottom.

**C**. Bar plots showed the selected biological functions enriched in fibroblast, FibSmo, and smooth muscle cells. Key genes were listed for enhanced GO term of different cell types.

We appreciate the effort the authors have made to support their findings with pre-existing datasets and consider that this additional line of evidence makes the results more robust. Since the expression of ACTA2 and MMP2 was seen in multiple clusters of existing datasets, it would be useful if authors could include BMP4, BMP5, PLAT in the Response Figure 4.

**Response:** Thanks for the positive comment and the thoughtful suggestion. As suggested, we included the expression levels of *BMP4*, *BMP5*, and *PLAT* in the above **Response Figure** **4**. We observed consistently high expression of *BMP4*, *BMP5*, and *PLAT* in FibSmo cells in the validation datasets. We also updated the results in **Supplementary Figure 7** in the revised manuscript.

4) The identification of rare/novel cell types (e.g. Langerhans cells and COCH+ fibroblast subtypes) should be supported by replicates/validation.

***Response(R1):*** *Thanks for the comment. To address the comment, we performed immunofluorescence staining assays to confirm the presence of rare/novel cell types identified in our study. We managed to locate sweat gland epithelial cell in two independent skin samples, which were stained with makers KRT19, SCGB2A2, and PIP (Additional file 5: Figure S3C). Similarly, we validated the novel fibroblast subtype by staining markers COCH and MMP2 in the skin and heart samples from two independent individual donors (Figure S3C). Importantly, we confirmed the novel cell type FibSmo with markers MMP2 and ACTA2 in the bladder, heart, and rectum samples from two independent individual donors (Figure S4A). Please note that we don’t examine the presence of Langerhans cell in tissue samples, because this cell type has been widely reported (Merad et al., Nat Rev Immunol 2008, 8:935-947; Clayton et al., Front Immunol 2017, 8:1676).*

*We have added the above results in this revision. Please kindly check in Line 15-18, Paragraph 2, Page 6 in the RESULTS section.*

The immunofluorescence results in S3C and S4A show only one of the two donors and some of the stainings are dubious since they look like background signal, e.g. SCGB2A2). In addition, the genes chosen as specific to rare populations instead seem to be expressed across many cells imaged.

Did the authors perform appropriate IF controls? Did the authors also report the results of the second donor? Did the authors also detect these novel cell types in the Human Cell Landscape from Han et al 2020 or other datasets? Do the authors have any hypothesis as to which could be the function of the novel fibroblast subtypes?

**Response:** Thanks for the suggestions. Yes, we previously provided representative immunofluorescence (IF) images for only one donor in the previous Figure S3C and S4A, although the IF assay was done in samples from two donors. To address the Reviewer’s comments, we have performed IF assays with more samples. All the results from multiple donors were provided as Supplementary Figures.

For sweat gland epithelial cell, we repeated multiplex IF assays in the two donors (donors 1 and 2) as in our previous manuscript. Again, co-localization of SCGB2A2, KRT19, and PIP proteins were identified for the *SCGB2A2*^+^ sweat gland epithelial cells (**Response Figure 6**). We note that the expression of SCGB2A2 is profound in skin cells but is limited to a certain cell type. This is consistent with the observation in another skin sample (donor 3), which was stained with only SCGB2A2 as a positive control (**Response Figure 6**).

For FibSmo cells, we performed multiplex IF assays in additional samples and presented the representative IF images for all samples. We observed co-localization of MMP2 and ACTA2 proteins in numbers of FibSmo cells in bladder (4 donors), heart (2 donors), and rectum samples (2 donors; **Response Figure 7**). FibSmo shared common stromal functions with fibroblasts and smooth muscle cells, such as *extracellular structure organization* and *tissue remodelling*, while FibSmo cells had enhanced biological functions in *response to wounding* and *response to growth factor*, which is consistent with the functions of their highly expressed signature genes (**Response Figure 5C**). For more information regarding the gene expression signatures and functions, please kindly refer to the Reviewer’s comment 2 above.

For *COCH*^+^ fibroblasts, we also repeated the multiplex IF analysis in the previous two donors, which revealed co-localization of COCH and MMP2 staining in the 4 skin and heart samples (**Response Figure 8**).

In summary, the multiplex IF assays in additional samples confirmed the presence of *SCGB2A2*^+^ sweat gland epithelial cells, FibSmo cells, and *COCH*^+^ fibroblasts in human. Consistently, the presence of such cell types was also validated in human samples with publicly available single cell sequencing data. Please kindly refer to our response to the Reviewer’s point 2 above. We note that the identifications of these cell types were based on co-expression of multiple cell markers, which is not specifically expressed for a certain cell type. Therefore, it is plausible that we might observe a broad expression of one of these markers across different cell types. We acknowledge that further investigations could be done to identify more specific markers for these cell types.

**Response Figure 6**. Representative images of immunofluorescence staining in skin samples for sweat gland epithelial cells. Proteins used in the assays are indicated at the left, and the samples from different donors (1-3) were listed as columns. The red arrows indicated the co-expression of SCGB2A2, PIP, and KRT19 proteins in cells in donor 1 and 2. Donor 3 was severed as positive control of SCGB2A2 staining. The red arrows indicated the specifically expression of SCGB2A2 in certain cell types in the skin in donor 3.

**Response Figure 7**. Representative images of immunofluorescence staining for FibSmo cells in bladder, rectum, and heart tissues. Proteins used in the assays are indicated on top, and the bladder, heart, and rectum samples from different donors (1-4) were listed as rows. The red arrows indicated the co-expression of ACTA2 and MMP2 proteins in tissue cells. For each sample, the right panel highlighted in a box was imaged field different from the left panel on the same tissue slide.

**Response Figure 8**. Representative images of immunofluorescence staining for *COCH*^+^ fibroblast cells in skin and heart tissues. Proteins used in the assays are indicated on top, and the skin and heart samples from different donors (1-2) were listed as rows. The red arrows indicated the co-expression of COCH and MMP2 proteins in cells of samples as indicated at the left. For each sample, the right panel highlighted in a box was imaged field different from the left panel on the same tissue slide.

We thank the authors for the additional data. Still, it remains hard to interpret the data given the lack of negative controls and negative cells within the imaged field. The authors should ensure that the detected signals are specific with appropriate negative controls.

**Response**: Thanks for the comment and suggestion. We fully understand the Reviewer’s concern on the specificity of an immunostaining assay. In our study, we ensured that the immunofluorescence staining signals were specific to each protein of interest. We did observe specific staining of SCGB2A2, PIP, and KRT19 proteins in sweat gland epithelial cells (above **Response Figure 6**) and MMP2 protein in tissue cells (above **Response Figures 7** and **8**), with a clear distribution of both positive and negative cells in the same imaged fields. By contrast, we observed broad expression of ACTA2 and COCH in tissue samples. This might reflect the abundant endogenous expression of the proteins. Indeed, ACTA2 protein has been shown with broad expression in the bladder and rectum tissues according to the Human Protein Atlas portal (https://www.proteinatlas.org/ENSG00000107796-ACTA2/tissue/rectum; https://www.proteinatlas.org/ENSG00000107796-ACTA2/tissue/urinary+bladder). Moreover, we provided additional images for the same tissue slides for each sample, where we observed more negative staining of cells compared to the previous imaged fields (above **Response Figures 7** and **8:** right panels). Furthermore, we performed IF with negative controls and observed no staining signals of ACTA2 and COCH in the tissue samples (**Supplementary Figures 9** and **10**, reproduced as **Additional** **Response Figure 1** below). Together, these observations indicate that the staining of proteins of interest are specific.

**Additional** **Response Figure 1.** Representative images of negative controls for immunofluorescence staining assays. Multiplex IF assays were performed using the same protocols as in **Response** **Figures** **7** and **8** but without primary antibodies recognising COCH (**A**) and ACTA2 (**B**). Tissues of origins are shown on top. Tissue sections were derived from the identical tissue samples of Donor 1 as in above **Response Figures 7** and **8**.

5) The authors should provide a complete description of the tissue dissociation method. In addition, they should comment on how the use of different dissociation protocols could result in selective release of cells in different organs and/or induction of stress response.

***Response(R1):*** *Thanks for the suggestions. As suggested, we now provide a detailed description of tissue dissociation protocols in Additional file 30, which includes the procedures as well as the use of enzymes and treatment durations for each organ. We note that these disassociation protocols were established based on the previous publications and rounds of in-house tests on different pig and human tissues until we could achieve high cell viability, less digestive time and less residual tissue.*

*As pointed out by the Reviewer, cellular dissociation was a prerequisite technical manipulation for single cell studies, although dissociation procedures would cause disturbance of transcriptome at various degrees (Brink et al., Nat Methods 2017, 14:935-936). Enzymes and duration of dissociation procedures might be two important factors (Harris et al., Hippocampus 2019, 29:876-882; Brink et al., Nat Methods 2017, 14:935-936). A gentle enzyme and short time might help to minimize the disturbance of transcriptome or induction of stress response. Moreover, an organ consists different cell types and extra cellar matrix, which need different enzymes to dissociate them into single cells. It’s unlikely that one enzyme could fit for all organs or cell types. Long duration and high concentration of enzymes could have a higher degree of dissociation. However, it comes with higher level of damage on cells and disturbance of transcription. Collagenase could only degrade the triple-helical native collagen fibrils; Dispase cleaving fibronectin, type IV collagen and type V collagen are gentle and have limited damage to cells; and Trypsin could cleave polypeptide chain, but could damage the integrity of cell membranes and reduce cell viability when treating tissues with a long duration. Considering the properties of different enzymes and the heterogeneous cell types in organs, we have optimized the protocols to achieve better dissociation and higher cell viability for each organ. We observed well clustering of the major cell types independently, which derived from different organs, suggesting that the dissociation procedures had minimal effects on the transcriptional profiles for the majority of cells. For more evidence, please refer to our response to the below comment Point 12 (Response(R1) Pages 48-52). However, we could not rule out the possibility that the dissociation procedures might have impact on some certain cell types, which await further investigations.*

*As suggested, we made comments in the DISCUSSION section. Please kindly check in Paragraph 2, Page 22. We also provided detailed information for the dissociation procedures and the evaluation of potential effects of dissociation-induced stress on the transcriptome as Supplementary Methods (Additional file 30).*

Thank you for adding the detailed protocols. They could be summarized in a table as there are high similarities across organs. The table can display the exact conditions for each organ. Indicate the concentration of dispase, not just the volume. Correct “disassociation protocols” for “dissociation” and “HAPA1A” for “HSPA1A”.

**Response:** Thanks for the suggestions and pointing out the typos. As suggested, we provided a supplementary table to summarize dissociation protocols for each organ, including primary dissociation reagents, addition of CaCl_2_, dissociation buffer, incubation condition and extra dissociation. Please kindly check at **Supplementary Table 51**, which was reproduced as below. As such, in this revision we have deleted the literal protocols described in the previous submission. In addition, we have corrected the typos in this revision.

**Response Table 1**. The digestion protocols for each organ.

| **Tissue** | **Primary dissociation reagents** | **Addition of CaCl_2_** | **Dissociation buffer** | **Incubation condition** | **Extra dissociation (optional)** |
| --- | --- | --- | --- | --- | --- |
| **Blood** | 0.8% NH_4_Cl red blood cells lysis buffer | NO | RBC buffer | 10 min on ice | NO |
| **Bone marrow** |  |  |  |  |  |
| **Bladder** | 125 U Dispase/5 ml + 1 mg/ml Collagenase IV + 1 mg/ml Collagenase II + 50 ug/ml DNase I | 5 mM | DMEM + 10%FBS | 1 h + 10 min | 0.25% trypsin + EDTA 20 min for residue |
| **Common bile duct** |  |  |  | 40 min + 10 min |  |
| **Esophagus** |  |  |  | 1 h + 10 min |  |
| **Heart** | 50 U Dispase/5 ml + 1 mg/ml Collagenase IV + 1 mg/ml Collagenase II + 50 ug/ml DNase I | 5 mM | DMEM + 10%FBS | 40 min + 10 min |  |
| **Lymph node** |  |  |  |  |  |
| **Muscle (leg)** |  |  |  |  |  |
| **Spleen** | 0.5 mg/ml Collagenase IV + 0.5 mg/ml Collagenase II + 50 ug/ml DNase I | 5 mM | DMEM + 10%FBS | 30 min + 10 min |  |
| **Small intestine** | 0.05% Trypsin + EDTA+ 0.5 mg/ml Collagenase IV + 0.5 mg/ml Collagenase II + 50 ug/ml DNase I | 5 mM added after 20 min | DMEM | 1 h + 10 min |  |
| **Rectum** | 0.05% Trypsin + EDTA+ 1 mg/ml Collagenase IV + 1 mg/ml Collagenase II + 50 ug/ml DNase I | 5 mM added after 20 min | DMEM | 1 h + 10 min |  |
| **Stomach** |  |  |  |  |  |
| **Liver** | Step 1: 1 mM EGTA + 1xDPBS, Step 2: 1 mg/ml Collagenase II + 1 mg/ml Collagenase IV + 50 ug/ml DNase I | 5 mM added after 10 min | Step 1: 1x DPBS, Step 2: DMEM + 10%FBS | Step 1: 10 min, Step 2: 30 min + 10 min |  |
| **Skin** | Step 1: 0.25% Trypsin + EDTA, Step 2: 125 U Dispase/5 ml + 1 mg/ml Collagenase I + 1 mg/ml Collagenase V + 50 ug/ml DNase I | 5 mM added after 20 min | DMEM | Step 1: 20 min, Step 2: 1 h + 10 min |  |
| **Trachea** | Step1: 0.25% Trypsin + EDTA, Step 2: 125 U Dispase/5 ml + 1 mg/ml Collagenase IV + 1 mg/ml Collagenase II + 50 ug/ml DNase I | 5 mM added after 20 min | DMEM | Step 1: 20 min, Step 2: 1 h + 10 min |  |

Ok, thank you.

The assessment of stress-induced transcriptomic effects is valuable and the fact that the identified clusters are not tissue-specific or dissociation-dependent is convincing. Nonetheless, the second and third points raised in pages 9 and 10 of the additional file 10 (“…FOS, HSPA1A and SOCS3, were not consistently expressed in all cell types in muscle…” and “dissociation-related genes showed different expression pattern in a same organ where all cells were treated with a same dissociation procedure”) are not conclusive as to whether there is a stress-induced gene expression effect or not and it may be confusing to keep those points in the text.

**Response:** Thanks for the suggestion. In this revision, we have removed those points from the text. Also, we moved the assessment of stress-induced transcriptomic effects to the **Methods** and **Discussion** in this revision.

Ok, thank you.

7) The accuracy of cell type annotations throughout the manuscript is unclear.

***Response(R1):*** *Thanks for the comment. First, we handled our data and annotated cell* *types with analytic software and pipeline that have been applied in multiple studies (The Tabula Muris Consortium et al., Nature 2018, 562:367-372; Han et al., Nature 2020:581, 303-309). Second, we annotated all cell clusters with marker genes for each cell type, which are well-known or have been published previously (please kindly check the gene list and corresponding references in the Additional file 2: Table S3 in this revision). Specifically, for a subtype of major cell type, we named it “major cell XXX_high” based on the most specifically highly expressed marker gene XXX in the cluster in Additional file 2: Table S4-S19 in this revision (Table S3 in the previous version). Third, the paired TCR and BCR information confirmed the accuracy of T cell and B cell annotations, respectively, and the developmental trajectory coupled with TCR/BCR information further confirmed the accuracy of annotation for T/B cell subtypes. Fourth, our trajectory analyses revealed developmental relationship for immune cells, such as monocyte-macrophage, that are consistent with the previous understanding but at a higher single-cell resolution. Fifth, we observed that our findings are consistent with a very recent study in human organ samples (Han et al., Nature 2020:581, 303-309), in terms of the gene expression profiles of B cells and epithelial cells and the transcriptional factor activity between both digestive and non-digested tissues. Sixth, we identified multiple TFs for the regulation of cell development between cell types, of which some are well-known. For example, SPI1 are involved in the development of monocytes to macrophages. Moreover, some TFs for human B cells and plasma cells could be consistently found in a very recent study (Han et al. Nature 2020:581, 303-309). Lastly, as suggested by the Reviewer #2, we have also validated our discovery of novel/rare cell types using immunofluorescence staining assays. Taken together, these strongly suggest that the analytic procedures and the cell type annotations in our study are robust.*

See response below in point 8.

**Response:** Thanks.

8) The expression of marker genes should be shown in a heatmap or dot plot in the main figure.

***Response(R1):*** *Thanks for the suggestion. As suggested, we added t-SNE plots to show the expression of marker genes for major cell types in the main figure (Fig. 1C).*

This comment goes together with the one above (point 7) and the original suggestion was to show markers for each identified population in the form of a heatmap or dot plot. The authors have opted for showing feature plots of a subset of markers for some annotations, however, for other annotations it remains unclear which markers are they based on, e.g. which marker were used to distinguish central memory from naïve T cells or effectors from resident memory? Showing the expression of canonical marker genes for known populations in a transparent way is required. Showing the expression of marker genes for all populations would be a more complete and transparent way to present the data.

**Response:** Thanks for the suggestions. As suggested, we have provided marker genes and their expression for each cell types in this revision, with dot plots or violin plots. Please kindly check at main **Figure 1C**, **Figure 2C** and **D**. For example, in the manuscript **Figure 1** (**B** and **C**), reproduced as below **Response Figure 9**, we presented a dot plot showing the expression of a subset of markers for major cell types (**Response Figure 9B**).

We also added violin plots to show the canonical markers of CD4^+^ and CD8^+^ cells (Zhang *et al., Nature* 2018, 564:268-272; **Response Figures 10** and **11**). We note that *CD4* gene was only significantly detectable in several CD4^+^ T cell clusters (**Response Figure 10B**), which might be due to the low presentation of 10x Genomics technology, while *CD8A* and *CD8B* were broadly expressed in all CD8^+^ T cell clusters (**Response Figure 11B**). We were not able to distinguish T_N_ from T_CM_ using CD45RA^+^CD45RO^-^CCR7^+^ and CD45RA^-^CD45RO^+^CCR7^+^ for the two cell types, respectively (Schmueck-Henneresse *et al., J Immunol* 2015,194(11):5559-67), since our data generated using 10x Genomics is not feasible for the determination of different *CD45* isoforms. Therefore, in our study, we used *CCR7*, *SELL*, *TCF7*, and *LEF1* as markers for T_N_ (naive T cell) and T_CM_ (central memory T cell) according to previous literatures (Hashimoto *et al*., *Proc Natl Acad Sci USA* 2019, 116:24242-24251). A cell cluster with high expression of both *CCR7* and *SELL* (lnFC greater than 0.7 for CD4^+^ and 1.0 for CD8^+^) in the top 10 rankings of DEG was assigned as T_N_ cells*.* Otherwise, clusters with expression of *CCR7* and *SELL* were assigned as T_CM_ cells.

**Response Figure 9**. Cells distribution across different organs and the marker genes of each cluster.

**A**. t-SNE showed the distribution of cells from different tissues coloured according to their origins of organ. Each dot represents a cell. The labelled cell types were the predominant cell types in each cluster.

**B**. Dot plots showed the most highly expressed marker genes (x axis) of major cell types (y axis) in **A**. The depth of colours from white to blue and the size of dot represent the average expression from low to high and the percentages of cells expressing the gene, respectively.

We observed different expression of marker genes between effector T cells (T_EFF_) and resident memory T cells (T_RM_; **Response Figure 11**). *FGFBP2* and *TBX21* were common markers for CD4^+^ and CD8^+^ T_EFF_ cells*,* with additional specific markers *GNLY* and *FCGR3A* for CD4^+^ and CD8^+^ T_EFF_ cells, respectively; *MYADM*, *NR4A1*, *CXCR6*, and *ITGAE* were markers for CD4^+^ T_RM_ cells; and *ITGAE*, *XCL1*, and *XCL2* were markers for CD8*^+^* T_RM_ cells (**Response Figure 11**). Moreover, T_EFF_ from T_RM_ cells also showed distinct expression signatures (**Response Figure 12**). Only T_EFF_ cells (NKG7_T_h1_, TRBV4-2_T_EFF_, MT1E_T_EFF_, and GNLY_T_EFF_) exhibited simultaneously strong expression of effector or cytotoxic genes including *GZMA*, *GZMB*, *PRF1*, *GZMH*, and *NKG7* (Zhang *et al., Nature* 2018, 564:268-272; **Response Figure 12**). In addition, we didn’t use *CD69* in combination of *ITGAE* to classify T_RM_, because *CD69* was detected widely in both CD4^+^ and CD8^+^ T clusters.

In this revision, we have also added the above results in the manuscript. Please check at the main **Figure 2C** and **D**.

**Response Figure 10**. Cell clusters and expression of canonical markers for each CD4^+^ T cells.

**A**. t-SNE plot of 7,006 CD4^+^ T from 15 organ tissues. Each point represents a cell, coloured according to their origins of organ (top panel) or cluster (bottom panel) as indicated on the right legend.

**B**. Violin plots showed the normalized expression of marker genes for each CD4^+^ T cell cluster as indicated at the bottom. For each panel, y-axis shows the normalized expression level for a marker gene as indicated at the left. Marker genes were also grouped according to functional cell types.

**Response Figure 11**. Cell clusters and expression of canonical markers for each CD8^+^ T cells.

**A**. t-SNE plot of 11,286 CD8^+^ T from 15 organ tissues. Each point represented a cell, coloured according to their origins of organ (top panel) and cluster (bottom panel) as indicated on the right legend.

**B**. Violin plots showed the normalized expression of marker genes for each CD8^+^ T cell cluster as indicated at the bottom. For each panel, y-axis shows the normalized expression level for a marker gene as indicated at the left. Marker genes were also grouped according to functional cell types.

**Response Figure 12**. Normalized expression of effector molecules in CD4^+^ and CD8^+^ T cell clusters.

**A. B**. Violin plots showed the normalized expression of effector molecules for each CD4^+^ and CD8^+^ T cell cluster as indicated at the bottom. For each panel, y-axis shows the normalized expression level for an effector molecule as indicated at the left.

We thank the authors for adding these plots for marker genes. CCR7 and SELL are markers for both naïve and central memory T cells and this is how they are reported in the cited paper (Hashimoto et al., Proc Natl Acad Sci USA 2019, 116:24242-24251). We would therefore suggest the reannotation of these cells as naïve/central memory T cells as the difference between the two subtypes cannot be reliably established.

**Response:** Thanks for the thoughtful suggestion. Although both naïve and central memory T cells express *CCR7* and *SELL*, we noted higher expression of *CCR7* and *SELL* in naïve T (T_N_) cells compared to central memory T (T_CM_) cells. Please kindly refer to the Figure 3B in the original paper by Cano-Gamez *et al.* (*Nat Commun* 2020, 11:1801), which was reproduced as below **Additional** **Response Figure 2** for a better reference. In our study, a high expression of both *CCR7* and *SELL* (lnFC greater than 0.7 for CD4^+^ and 1.0 for CD8^+^) among the top 10 rankings of DEG in each cluster was used as criteria to distinguish T_N_ from T_CM_ cells. Indeed, we observed the initial state of differentiation at the starting root for T_N_ cells in the trajectory analyses (LINC00861_T_N_ and SELL_T_N_ for CD4^+^ and CD8^+^ T cells, respectively; **Response Figures 13** and **14**). We also observed intermediate state and terminal state for KLF2_T_CM_ and STMN1_T_CM_, respectively, in the developmental trajectories of CD4^+^ cells (**Response Figure 13**). However, for the other cell clusters, we had less evidence to group them accurately as either T_N_ or T_CM_. Therefore, we reannotated these cells as T_N_/T_CM_ according to the Reviewer’s suggestion. We have updated the relevant content in the revised manuscript, figures, tables, and supplementary materials. Please kindly check at the lines 5, 8-10 in the 2^nd^ paragraph, Page 7, and **Figure 2C** and **D** in the main text; **Supplementary Table S22-S27** and **Supplementary Figure 13** and **14**.

**Additional Response Figure 2.** Marker genes of each CD4^+^ T cell cluster. Colours encode the mean expression of each gene in each cluster. *For the purpose of quick reference, the figure was reproduced from the Figure 3B in the original paper by Cano-Gamez *et al*.

9) Automated doublet detection should be compared to manual exclusion of doublets. What do authors refer to as "unassigned" cell types (e.g. "Clusters due to cell doublets or unassigned cell types were identified and excluded manually <..>")?

***Response(R1):*** *Thanks for the comments. Initially, we had applied DoubletFinder to our data. We observed that only a few cells were inferred as doublets and some proportion of cells were still self-clustered and deviated from other cells, meaning that these are likely doublets overlooked by the automated software. Therefore, we applied a more stringent method, which was based on the similarity of the markers and gene expression profiles of cell types to determine doublets manually. As suggested, we compared two methods for the removal of doublets, namely the automated detection and manual exclusion, using muscle and heart as examples. We found that 15.76% and 10.8% of cells were identified as doublets manually in muscle and heart samples, respectively. By contrast, 5% of cells were inferred as doublets for both samples using the automated DoubletFinder. Moreover, the doublets identified manually cover 47.06% (216 out of 459) and 65.73% (234 out of 356) doublets identified by DoubletFinder in the muscle and heart tissues, respectively. In order to minimize the potential impact of these doublets on the downstream analyses, we have applied the manual method as a more conservative way to remove doublets in our dataset. For more detailed comparison results including figures, please kindly refer to our response to the comment raised by the Reviewer #1 (point 3 in Response(R1) Page 9-13). In this revision, we have added above descriptions in Supplementary Methods (Additional file 30).*

*We defined doublets as any cell with 1) expression of multiple markers of different cell types, 2) a gene expression profile overlapping with multiple cell types, and 3) more than twice median numbers of UMIs and genes compared to major cell types within each organ, which were removed manually. The “unassigned cells” were those with less confidence to define as doublets, meeting only one or two of the above three criteria. The “unassigned cells” formed clusters in the bladder, lymph node, muscle, skin and the merged data, independently. DoubletFinder could not identify these cells as normal or doublets. Therefore, we applied a conservative way by defining both mixed cells and "unassigned cells” as doublets and removing them from subsequent analyses. In this revision, we have updated the definition in the METHODS section. Please kindly check in the last sentence in Paragraph 1, Page 26.*

The authors of DoubletFinder recommend users to threshold doublet calling by calculating the mean-variance-normalized bimodality coefficient of pANN distributions produced during pN-pK parameter sweeps of the data. BCMVN can be used to identify the pK that separates singlets and doublets effectively. Have the authors optimized the threshold for this specific dataset? This may be contributing to the low doublet detection by DoubletFinder.

The authors mention that “the doublets identified manually cover 47.06% (216 out of 459) and 65.73% (234 out of 356) doublets identified by DoubletFinder in the muscle and heart tissues”. Since there is limited overlap between the two methods, the authors may want to combine the doublets detected by DoubletFinder with the manually detected ones. Correct “comparation” for “comparison”.

**Response:** Thanks for the suggestions. As suggested, we combined both “DoubletFinder” and then manual detection methods to identify and remove the doublets in each organ. For “DoubletFinder”, we set an estimated proportion of doublets as 5% and an optimized pK using BCMVN for each organ (**Response Table 2**). We then manually removed the potential doublets clusters in each organ as we mentioned in the previous submission. After that, we rerun all the downstream analyses and updated the results, related figures and tables in this revision. Compared to the previous submission, additional 4,275 ‘cells’ were determined as doublets and removed from the analyses, resulting in a total of 84,363 cells. We note that the removal of new doublets had minimal impact on our findings. In this revision, we have updated the methods in the **Methods** section and the relevant results in the main text. Please kindly check at the 2^nd^ paragraph, Page 27 to the 1^st^ paragraph, Page 28; and **Supplementary Table 51**. As such, we also removed the comparison of ‘DoubletFinder’ and the manual detection methods described in the previous supplementary methods. In addition, we corrected the typo ‘comparation’ to ‘comparison’. We sincerely apologized for this overlook.

**Response Table 2**. Optimal pK values for each organ.

| **Tissue** | **Optimal pK** |
| --- | --- |
| Bladder | 0.005 |
| Blood | 0.02 |
| Common bile duct | 0.29 |
| Esophagus | 0.005 |
| Heart | 0.005 |
| Liver | 0.005 |
| Lymph node | 0.005 |
| Marrow | 0.02 |
| Muscle | 0.01 |
| Rectum | 0.005 |
| Skin | 0.28 |
| Small intestine | 0.005 |
| Spleen | 0.01 |
| Stomach | 0.01 |
| Trachea | 0.005 |

Ok, thank you.

10) The pseudotime analysis of T cells and myeloid cells reflects transcriptional similarities. The authors overinterpret the connection of these trajectories to the developmental stage of cells, provide no new insights or lack of experimental validation or further supporting data. The analysis of gene expression dynamics along pseudotime could add value to this analysis.

***Response(R1):*** *Thanks for the suggestion. Initially, we had tried to analyze gene expression dynamics along pseudotime using monocle 2. We observed multiple branches and nodes in the trajectory trees for T cells and myeloid cells, however, we did not observe a clear dynamic change of genes related to the development. These might be explained by that monocle 2 could not handle cells with too many subtypes (Response(R1) Figure 10 A-C). The new version of monocle (monocle 3 alpha and 3) could handle more complicated trajectory analysis with more different developmental states of cells (http://cole-trapnell-lab.github.io/monocle-release/monocle3/). However, too many developmental nodes identified by monocle3 alpha make it hard to resolve gene expression dynamics along pseudotime like in monocle 2 with limited nodes.*

*Alternatively, we presented the expression of genes that are known to regulate the corresponding cells in the pseudotime plots as in Fig. 2C, D and Additional file 10: Figure S7G-H in this revision. In general, we observed consistency between the development of cells and the expression of marker genes regulating the developmental process. For examples, TCF7 and LEF1 are Wnt pathway effectors and were reportedly expressed at the highest levels in naïve and central memory cells and lower levels in transitional and effector memory cells (Billingsley et al., PLoS Pathog 2015, 11:e1004740). We observed high activity and expression of TCF7 and LEF1 in CD4^+^, CD8^+^ T_N_, and T_CM_ cell clusters (Fig. 2D, E, and Figure S7G, H). FOXP3 is a specific marker of Treg cells and necessary for their suppressive activity on T cells (Hori et al., Science 2003, 299:1057-1061). We observed that FOXP3 was only enriched in CD4^+^ Treg cluster in the pseudotime plot and had higher activity in this cluster (Fig. 2D). Similarly, we observed that SPI1 had higher activity in monocytes and had higher expression level in corresponding monocyte clusters (Fig. 4F, G).*

*We have updated the results in the main text. Please kindly check in the RESULTS section in Paragraph 2, Page 8; Additional file 10: Figure S7G, H; Paragraph 2, Page 13 and Fig. 4G.*

*Response(R1) Figure 10. Pseudo-time of CD4^+^, CD8^+^ T cells, and monocytes/macrophages*

*A. Pseudo-time of CD4^+^ inferred by Monocle2 (left panel). Each point corresponds to a single cell. Clusters information was shown at the right panel. Selected genes expression along the psedutime were shown in the right panel. TCF7 and LEF1 for T_CM_ and T_N_, STAT1 and IRF1 for T_EFF_, FOXP3 for T_reg_, and FOS for T_RM_.*

*B. Pseudo-time of CD8^+^ inferred by Monocle2 (left panel). Each point corresponds to a single cell. Clusters information was shown at the right panel. Selected genes expression along the psedutime were shown in the right panel. TCF7 and LEF1 for T_CM_ and T_N_, STAT1 and TBX21 for T_EFF_, and FOS and ATF3 for T_RM_.*

*C. Pseudo-time of monocytes and macrophages, inferred by Monocle2 (left panel). Each point corresponds to a single cell. Clusters information was shown at the right panel. Selected genes expression along the psedutime were shown in the right panel. RXRN and ELF2 for classical monocytes, TCF7L2 and STAT1 for non-classical monocytes and LIPA_macrophage, and JUN and PRDM1 for most of non-classical monocyte clusters.*

Validating known markers, such as FOXP3 in Tregs, is not the purpose of trajectory analysis. As it stands now, this trajectory analysis does not lead to new biological insights and it may lead to confusion. Regulatory T cells (8) in Fig. 2C are standing next to Tcm(3). Do the authors think this makes biological sense? What is their explanation for this? Also, the central memory CD4 T cells in cluster (1) are at the end of the trajectory. What is the authors’ hypothesis regarding this feature of their trajectory?

**Response:** Thanks for the inspirational questions and suggestions. As pointed out by the Reviewer, our trajectory analysis revealed the branching out of CTLA4_T_reg_ (previous Treg (8)) cells next to KLF2_T_CM_ (previous Tcm (3)) cells along the development trajectory from T_N_ cells to T_RM_ cells (**Response Figure 13**). We note a similar observation reported most recently in T cells (Cano-Gamez *et al., Nat Commun* 2020, 11:1801), where human naïve T cells developed towards central memory, then effector and finally highly effector memory T cells, with natural Treg cells branching out from central memory T cells. Please kindly refer to the Figure 3C in the original paper by Cano-Gamez *et a.*, which was reproduced as below **Response Figure 14** for a better reference. These observations might suggest the conversion/differentiation potential from central memory T cells to Treg cells, which has been demonstrated previously using *in vitro* models with TGF-β stimulation (Zhang *et al., PLoS One* 2013, 8(10):e77322).

**Response Figure 13**. Pseudo-time trajectory analysis of CD4^+^ T cells with high variable genes. Each dot represents a single cell and is coloured according to their clustering in the right legend. The inlet t-SNE plot shows each cell with a pseudo-time score from dark blue to yellow, indicating early and terminal states, respectively.

**Response Figure 14**. Branched pseudotime trajectory, each cell is coloured by its pseudotime value (left panel) or its cluster label (right panel). *For the purpose of quick reference, the figure was reproduced from Figure 3C in the original paper by Cano-Gamez *et al*.

Also, as pointed out by the Reviewer, a clear central memory T cell cluster (STMN1_T_CM_, previous Tcm (1)) is at the end of the trajectory, likely differentiated from tissue resident memory T cells (TNF_T_RM_, previous T_RM_ (2)) according to the pseudotime scores. Together with that STMN1_T_CM_ cells were collected from lymph nodes, this observation provides a clue to solve the puzzle whether tissue-homing memory T cells further differentiate or can migrate back to the lymphoid compartment (Lees *et al., Immunology*. 2010, 130(4): 463-470). Supportively, a very recent study using mice model demonstrated that T_RM_ cells in the skin could differentiate into T_CM_ and T_EM_ cells upon local reactivation, which then rejoined the circulation (Fonseca R *et al., Nat Immunol* 2020, 21:412-421). Taken together, these observations suggest that T_RM_ cells have developmental plasticity rather than representing a terminal stage of differentiation.

Compared to other T_CM_ clusters, STMN1_T_CM_ was at terminal development stage expressing mediate level of *FOS*/*JUN* and high level of *CTLA4*, an active marker of CD4^+^ T cells. To further investigate the development of the STMN1_T_CM_ cells, we performed trajectory analysis with TNF_T_RM_ and STMN1_T_CM_ clusters, which revealed that TNF_T_RM_ from two branches with early state gradually progressed towards STMN1_T_CM_ with terminal state (**Response Figure 15**). T_RM_ cells at the beginning of the trajectory expressed high levels of T_RM_ markers, such as *RUNX3*, *NR4A1*, chemokines (*CCL5*), and other T_RM_ associated genes, including *ID2*. By contrast, T_CM_ cells at the end of the trajectory was marked with expression of well-known T_CM_ molecules, such as *SELL* and *CCR7.* In addition, some other genes that are poorly characterized were also enriched in T_CM_ cluster, including genes encoding ribosomal proteins and *TXNIP*.

In this revision, we have added the above information in the **Discussion** section. Please check at the last three sentences in the last paragraph, Page 20; the last three sentences in the 1^st^ paragraph, Page 21.

The cited study (Cano-Gamez et al., Nat Commun 2020, 11:1801) reports data on a highly controlled system where naïve and memory T cells are polarized *in vitro* towards four helper phenotypes (Th1, Th2, Th17 and iTreg) as well as the effectorness gradient in resting CD4+ T cells derived from peripheral blood. The authors are careful not to claim that these cells are differentiating from central memory T cells, as this cannot be concluded based on this analysis. Their trajectory analysis is interpreted in terms of effectorness. We would therefore advice the authors to adjust their claims. Differentiation of Tregs from central memory T cells is possible, however, whether this is the case *in vivo* remains to be established with precise lineage tracing methods.

**Response:** Thanks for the thoughtful comment and suggestion. As suggested, we have adjusted the claims in our manuscript by not to overstate the potential differentiation of central memory T cells to Tregs. Please kindly check at the **Discussion** section, the last three sentences in the 1^st^ paragraph, Page 21, as read “In addition, we observed the branching out of CTLA4_T_reg_ cells next to KLF2_T_CM_ cells along the development trajectory from T_N_ cells to T_RM_ cells (**Figure 2E**). Given that previous *in vitro* studies have demonstrated a potential development from central memory T cells to Treg cells upon stimulation. This observation suggested that differentiation of Tregs from central memory T cells is possible, however, whether it holds true in vivo remains to be established with precise lineage tracing methods.”

The authors should note that Tgd are not CD8 T cells and MAIT are thought to develop via a different route in the thymus. Please, correct the data accordingly.

The authors should also take into account that gdT are not CD8 T cells, Tcm are CCR7+ and MAIT are thought to develop via a different route.

**Response:** Thanks for the suggestions. As suggested, we had excluded the γδ T cells in the previous revision. We further excluded MAIT cells from the trajectory analysis in this revision, which revealed similar differentiation trajectories for CD8^+^ T cells compared to that in previous submission. We observed that the trajectory tree rooted from T_N_ cells and sprouted out towards T_N/CM_, T_EM_, and T_RM_ branches for CD8^+^ T cell (**Response Figure 16**). T_EFF_, T_RM_, and IEL CD8^+^ T cells showed higher pseudo-time scores, suggesting their terminal development state (**Response Figure 16**).

In this revision, we have updated the above results accordingly. In addition, we used *CCR7*, *SELL*, *TCF7*, and *LEF1* as markers for naïve/central memory T cells (T_N/CM_). Please kindly refer to our response to the Reviewer’s point 8 above.

**Response Figure 15**. Pseudotime trajectory analysis and gene expression profiles of TNF_T_RM_ and STMN1_T_CM_.

**A**. Branched pseudotime trajectory with TNF_T_RM_ and STMN1_T_CM_. Each cell as dot is coloured according to a cluster label (left panel) or pseudotime value (right panel).

**B**. Expression heatmap of selected variable genes along the pseudotime trajectory (from Monocle). Each row represents a gene as indicated at the right; column bars represent cells ordered by pseudotime (from left to right) and different colours correspond to the scaled (Z-scored) expression of each gene in each cell.

**Response Figure 16**. Pseudo-time trajectory analysis of CD8^+^ T cells with high variable genes. Each dot represents a single cell and is coloured according to their clustering in the right legend. The inlet t-SNE plot showed each cell with a pseudo-time score from dark blue to yellow, indicating early and terminal states, respectively.

Ok, thank you.

Could the HSPA1A CD8+ population be a stress-induced artefact? Similar question for the Trm populations expressing FOS/JUN. How could the authors support the idea that these are not stress-induced genes?

**Response:** Thanks. We don’t think that *HSPA1A* CD8^+^ T population and the T_RM_ populations expressing *FOS*/*JUN* are stress-induced artefacts, although the upregulation of *HSPA1A*, *FOS*, and *JUN* has been reported in certain scenarios with stress stimulations such as enzyme digestion during dissociation process (van den Brink *et al., Nat Methods* 2017, 14:935-936).

First, we had provided lines of evidence to show that dissociation processing in our study might have minimal impacts on the expression of *HSPA1A*, *FOS*, and *JUN* as well as the transcriptome of majority of cells in our dataset. Please kindly refer to our previous ***Response(R1)*** to the Reviewer’s point 12 below. The Reviewer commented that “The assessment of stress-induced transcriptomic effects is valuable and the fact that the identified clusters are not tissue-specific or dissociation-dependent is convincing.” Please kindly refer to the **Reviewer’s comment at point 5** above.

Second, the majority of *HSPA1A* CD8^+^ T cells (86.2%; 1,072 out of 1,244) were detected in the common bile duct, showing a tissue-specific distribution as reported previously in a mouse study (Beura *et al.,* *Immunity* 2018, 48:327-338 e325). The study demonstrated that *Hspa1a* was only enriched in the female reproductive tract (FRT) CD8^+^ T_RM_, but not in the spleen T_RM_ and T_CM_ cells. Moreover, although similar dissociation conditions were applied to multiple organs (**Response** **Table 1**), high expression of *HSPA1A* was observed specifically in HSPA1A_T_RM_ and a few other cell clusters (**Response Figure 17**). Supportively, exclusively high expression of *Hspa1a* was observed in only one of two T follicular helper (TFH) like cell clusters but no other T cells types in the lung of mouse (Swarnalekha *et al., 2020 BioRxiv;* https://doi.org/10.1101/2020.02.28.963280). These observations suggest *HSPA1A* to be one of the specific markers for T cell subtypes.

Third, we suspected that high expression of *FOS* and *JUN* in CD8^+^ T_RM_ populations (**Response Figure 17**) might reflect their role in maintaining the cells rather than in response to the dissociation-related stress. *FOS* and *JUN* are members of AP-1 transcription factor, of which dimerization has been shown to play numerous roles in a wide range of cellular process, cell growth, and proliferation, etc. We did observe the upregulation and the high regulon’s activity of many AP-1 dimerization partners (such as *FOS*, *JUN*, *JUNB*, *FOSL2*, and et) for CD4^+^ (**Response Figure 18A** and **B**) and CD8^+^ T_RM_ cells (**Response Figure 17**, **Response Figure 18C**) in our dataset. It has been reported recently that AP-1 dimerization partners such as *Fosl2* and *Junb* were highly expressed in T_RM_ cells, and the knockdown of *Junb* resulted in impaired T_RM_ cell differentiation, confirming the key role of AP-1 dimerization regulating T_RM_ differentiation (Kurd *et at., 2020* BioRxiv; <https://doi.org/10.1101/2020.03.02.973578>). Given that *Fosl2* is able to regulate *Smad3* (Ciofani *et al., Cell* 2012, 151:289-303), a key component of the TGF*β* signalling pathway, *Fosl2* may also promote T_RM_ cell differentiation through effects on TGF*β* signalling. We note that high expression of *FOS* and *JUN* was only observed in most of T_RM_ cells, but not non-T_RM_ cells (**Response Figure 17, Response Figure 18A**) from the same organs that were undergone dissociation treatments with similar conditions. This observation, together with our previous observation that most organs had higher density of low total dissociation score (***Response(R1) Figure 11***), suggests that dissociation-related genes were not significantly induced in the majority of cells. Please also kindly refer to our previous response (***Response(R1))*** to the Reviewer’s point 12 below.

Lastly and importantly, we performed additional immunofluorescence assay with double staining of CD8A and FOS in multiple organs including common bile duct, liver, and small intestine from multiple donors, and confirmed the high expression of FOS in the CD8 cells before tissue dissociation (**Response Figure 19**).

In summary, these observations suggest that *HSPA1A* CD8^+^ T population and the T_RM_ populations expressing *FOS*/*JUN* are unlikely stress-induced artefacts. We have updated the data in the **Discussion** section in this revision. Please kindly check at the last sentence on Page 23 to the 1^st^ sentence on Page 24, and **Additional file 7 Supplementary Notes**.

**Response Figure 17.** The violin plots showed the normalized expression of *FOS*, *JUN*, and *HSPA1A* in CD8^+^ T cell clusters.

**Response Figure 18**. Gene expression and cluster-specific regulon activity of T cells.

**A**. Normalized expression of *FOS* and *JUN* in different CD4^+^ T cell clusters.

**B, C**. Heatmap of the active scores of T cell subtypes (**B** for CD4^+^ T, **C** for CD8^+^ T cells, respectively), as estimated using SCENIC analysis of transcription factors (TFs). Shown on the right are the top 10 TFs with the highest difference in the expression regulation estimates between each cluster (indicated on top) and all other cells, tested with a Wilcoxon rank-sum test. Filled colours from blue to red represent scaled TF activities from low to high.

**Response Figure 19**. Representative images of immunofluorescence staining of FOS and CD8A in small intestine, common bile duct, and liver tissues. Markers used are indicated on top. Samples were derived from donor as indicated at the left. Red arrows indicated the co-expression of proteins FOS and CD8A.

Ok, thank you.

12) The transcription factor signature in T cells that comprised FOS, FOSB, JUN, JUND, etc is concerning since these have been previously shown to be upregulated in response to tissue dissociation-induced stress (Brink et al, 2017 Nature Methods). The authors should explain how they can exclude this is not an artifact of the tissue processing.

***Response(R1):*** *Thanks for the suggestion. We fully understand the Reviewer’s concern about the potential effect of dissociation-induced stress on cells, which should be minimal on overall transcriptome. First, we evaluated the effect of tissue processing on cells in our data, using a method described previously (the Tabula Muris consortium et al., Nature 2018, 562:367-372). We observed that most organs had higher density of low total dissociation score, meaning that dissociation-related genes were not significantly induced in the majority of cells (Response(R1) Figure 11). Second, the typical dissociation-related satellite-cell genes (van den Brink SC et al., Nat Methods 2017, 14:935-936), such FOS, HSPA1A and SOCS3, were not consistently expressed in all cell types in muscle (Response(R1) Figure 12). HSPA1A had lower expression level in both two satellite-cell clusters than other clusters. SOCS3 only slightly expressed in one of two satellite-cell clusters. Third, dissociation-related genes showed different expression pattern in a same organ where all cells were treated with a same dissociation procedure (Response(R1) Figure 13). For example, in the small intestine and stomach, FOS, JUN, and JUNB broadly expressed in different cell clusters with various degree, while SOCS3 and HSPA1A only specifically highly expressed in adoptive cell, macrophage in the small intestine, and enriched in monocyte, fibroblast, and chief cell in the stomach. Fourth, we observed that same cell types from different organs treated with different dissociation procedures had similar expression pattern of the dissociation-related genes. Taking T, B, and plasma cells in the small intestine and stomach for example, each of them had similar expression pattern of those dissociation-related genes. Moreover, each major cell type from different organs was clustered together in t-SNE plot, suggesting similar transcription profiles in spite of different dissociation treatments (Fig. 1B).*

*Taken together, the above observations suggest that dissociation processing in our study might have a minimal impact on the transcriptome of majority of cells in our dataset. Nevertheless, we could not rule out the possibility that the dissociation procedures might have impact on some certain cell types, which await further investigations. Please kindly check at Paragraph 2, Page 22 in the DISSCUSSION section. We also provided detailed information for the dissociation procedures and the evaluation of potential effects of dissociation-induced stress on the transcriptome as Supplementary Methods (Additional file 30).*

*Response(R1) Figure 11. Dissociation scores for each organ. A score represents the magnitude of the first principal component of principal component analysis based on the expression of 140 human dissociation-associated genes of each organ (van den Brink SC et al., Nat Methods 2017, 14:935-936). The y axis shows the probability density of the normalized histogram.*

*Response(R1) Figure 12. Violin plot showed the normalized expression of typical dissociation-related genes in the muscle sample. Shown were SOCS3, FOS, and HAPA1A1.*

*Response(R1) Figure 13. Violin plot showed the normalized expression of typical dissociation-related genes in the small intestine and stomach samples. Shown were FOS, FOSB, JUN, SOCS3, and HAPA1A.*

Response above in point 5.

**Response:** Thanks. Please kindly refer to point 5 above.

14) Authors use pseudotime trajectory inference on circulating monocytes and macrophages pooled from all tissues, however, they fail to take into account that most tissue-resident macrophages are not derived from circulating monocytes but from embryonic progenitors (e.g. self-renewing embryonic macrophages in the skin, liver or gut). As mentioned above, the consideration of the biological context is essential for a robust interpretation of trajectory analysis.

***Response(R1):*** *Thanks for the thoughtful comments. We fully agree with the Reviewer that most tissue-resident macrophages are derived from embryonic progenitors and undergo self-maintenance via local expansion in the steady human organ, with minimal contribution from circulating monocytes (Hashimoto et al., Immunity 2013, 38:792-804; Jakubzick et al., Immunity 2013, 39:599-610; Sieweke MH et al., Science 2013, 342:1242974).*

*In our study, we performed trajectory analysis to investigate the potential developmental relationship between monocytes and macrophages. The developmental trajectory path from classical monocytes to tissue-resident macrophages suggests that classical monocytes could be a protentional origin of adult tissue-resident macrophages (Fig. 4E). One the other hand, high expression of proliferation markers including MKI67, PCNA, and TYMS were exclusively detected in the C12 macrophages (including those in bladder, common bile duct, esophagus, heart, and rectum). Moreover, GSEA analysis also revealed that most of the macrophage clusters had higher enrichment score of G2M checkpoint and E2F targets pathways (Additional file 14: Figure S10E). These observations suggest local expansion potential of tissue-resident macrophages. Therefore, our findings suggest tissue-resident macrophages in steady-state organs might have two origins, one from classical monocytes and another from self-expansion. However, with present data, it is hard to tell that self-expansion alone or slightly together with circulating monocytes contributes to the development of tissue-resident macrophages. Further investigations using cell labeling (Chen et al., Nat Commun 2018, 9:5065) or samples from donor who received bone marrow transplantation (Thomas et al., Science 1976, 192:1016-1018) may help address the question.*

*In this revision, although our observations suggest potential development relationship between circulating monocytes and tissue-resident macrophages, we acknowledge that “however, further investigations are awaited to address whether self-expansion alone or slightly together with circulating monocytes contributes to the development of tissue-resident macrophages, considering that most tissue-resident macrophages have been demonstrated originating from embryonic progenitor cells (Hashimoto et al., Immunity 2013, 38:792-804; Jakubzick et al., Immunity 2013, 39:599-610).” Please check in Lines 10-14, paragraph 2, Page 20 in the DISCUSSION section.*

Although authors have softened the language and incorporated the possibility that tissue-macrophages may be derived from embryonic cycling macrophages, the authors could have incorporated some comparisons with published fetal data, such as gut macrophages from Gao et al., 2018 (doi: 10.1038/s41556-018-0105-4). At minimum, the authors could provide novel pathways that change in monocyte-macrophage differentiation trajectory.

Thanks for revising this part. Still, the claim should be tone down and the header of the section should exclude the “origin of macrophages” part given that this analysis cannot reliably determine the origin of these cells but simply infer transcriptional similarities among those cells.

**Response:** Thanks for the suggestions. As suggested, we obtained the raw data from Gao *et al*.’s study (*Nature Cell Biology* 2018, 20:721–734) and identified 50 embryonic macrophages in 121 immune cells from fetal large-intestine samples. We further performed trajectory analysis inclusive the embryonic macrophages and 828 cells of monocytes and macrophages from rectum sample in our AHCA data (**Response Figure 20A**). Pseudotime analysis revealed that both embryonic and adult macrophages were at terminal state (**Response Figure 20A**). Moreover, we observed a clear differentiation trajectory of the macrophages in adult rectum from intermediate monocytes (CCL20_Intermediate_Mon) to tissue macrophages. Interestingly, the embryonic macrophages (Embryo_Mac) were found alongside the differentiation trajectory from the intermediate monocyte (FN1_Intermediate_Mon) to the tissue-macrophages (HIST1H4C_Mac), suggesting that embryonic macrophage may contribute to the tissue-macrophage through local expansion. Consistently, high expression of proliferation marker gene *PCNA* were detected in both the terminal macrophages (**Response Figure 20B**).

In this revision, we have added the above comparisons in the **Results** and **Discussion** sections as well as the related methods in the **Methods** section. Please kindly check at the 1^st^ paragraph, Page 13; the 1^st^ paragraph, Page 22; the last sentence in the 2^nd^ paragraph, Page 29. In addition, we removed “origin of macrophages” from the header as suggested.

**Response Figure 20**. Development trajectory of monocytes and macrophages in the intestine tissues.

**A**. Pseudo-time trajectory analysis of myeloid cells from rectum tissue in our AHCA dataset and macrophages from embryo large intestine in Gao *et al*.’s data. Each dot represents a single cell and is coloured according to their cluster. Only cell clusters with large than 50 cells in AHCA were included. The inlet t-SNE plot shows each cell with a pseudo-time score from dark blue to yellow, indicating early and terminal states, respectively.

**B**. Violin plots showed the normalized expression of proliferation marker genes (*MKI67* and *PCAN*) in each myeloid cell cluster as indicated at the x axis in the combined data of AHCA and Gao *et al*.’s samples.

Ok, thank you.

15) Epithelial cell analysis is focused on biology that has been previously described. The authors could use this dataset to further understand the core-similarities between epithelial cell subsets (e.g. secretory cells) across tissues as well as the key differences.

***Response(R1):*** *Thanks for the suggestion. As suggested, we performed additional analyses to examine the similarities of epithelial cells across different organs. First, we compared TFs in epithelial cell clusters, which revealed similar activation of TFs in most non-digestive clusters, as well as in digestive clusters (Fig. 5G). This was further confirmed using a recent published dataset (Additional file 19: Figure S13B). Second, pathway analysis revealed that multiple metabolic pathways were enriched in digestive clusters other than non-digestive cluster, suggesting the enhanced metabolic activity of digestive epithelial cells (Additional file 18: Figure S12C, D). Lastly, we performed ligand-receptor analysis for epithelial cells across different organs, which revealed that epithelial cells had the most frequent intra-cell interactions among all cell types, suggesting that epithelial cells could interact with each other intensively to regulate their biology functions in each organ (Additional file 24: Figure S17C and Additional file 19: Figure S21).*

*In this revision, we added the new results in the main text. Please kindly check in in Line 4-6, Paragraph 2, Page 14; Line 8-11, Paragraph 3, Page 14; Line 3-5, Paragraph 1, Page 15; Line 6-11, Paragraph 2, Page 15; Line 5-9, Paragraph 1, Page 16; Line 9-11, Paragraph 1, Page 21; and Line 16-18, Paragraph 2, Page 21.*

The authors should clarify which tissues do they classify as digestive tissue and what are the implications of this observation.

The detection of a “humoral immune response” GO term in epithelial cells suggests that there is a contamination in these cells. One possibility could be that ambient mRNA derived from lysed plasma cells led to the detection of plasma cell transcripts in these epithelial cells. Please, revise this point.

**Response:** Thanks for the thoughtful suggestions. According to the presence of digestive-related functions, stomach, small intestine, rectum, liver, and common bile duct were classified as digestive tissue in our AHCA data (**Response Figure 21A**), as were colons (ascending colon, colon, sigmoid colon, transverse colon), small intestines (duodenum, Ileum, Jejunum), liver, gallbladder, pancreas, stomach, and rectum in the Han *et al.*’s HCL dataset (**Response Figure 21B**). By contrast, all other tissues with detectable epithelial cells were considered as non-digestive tissue. Although esophagus is a part of digestive tract, epithelial cells from the tissue shared similar expression profiles with that in skin and trachea in both the AHCA and HCL datasets, indicating similar function of epithelial cells in esophagus compared to skin and trachea, but not other digestive organs (**Response Figure 21A, B**).

First, we investigated the contribution of TFs in regulating the heterogenous transcriptional profiles of epithelial cells in and between organs. SCENIC analysis revealed that the regulation of TFs was similar among cell clusters derived from a same tissue but was very different among cell clusters between tissues in both the AHCA and HCL dataset (**Response Figure 22**). Interestingly, the digestion-associated epithelial clusters exhibited similar activation of TFs, as did the cell clusters belong to non-digestive tissues including the trachea, skin, and esophagus (**Response Figure 22A**). Similar activation of TFs was observed between our AHCA and the HCL datasets for digestive-associated tissues (expect for pancreas) and non-digestive tissues (except for lung; **Response Figure 22B**). Moreover, we identified 204 TFs in our AHCA dataset, which overlap about 60% (120) of all cluster-specific TFs of the HCL dataset. Second, GO analysis revealed that distinct biological functions of cells between digestive tissue and non-digestive tissue. Metabolic process, energy synthesis, and digestion pathways were commonly observed in digestive tissue (**Response Figure 23A**), while *regulation of endopeptidase activity* and *epidermis development* were the strongly enriched pathways observed in non-digestive tissue (**Response Figure 23B**). Third, GSVA analysis also revealed significantly different pathways between digestive and non-digestive cell clusters, including multiple metabolic pathways with elevated activity in digestive cells and *TNFA signal via NFKB* in non-digestive epithelial cells (**Response Figure 21 C** and **D**). These observations indicate the diverse molecular profiles underlying the functional heterogeneity between the two groups of tissues, as well as the similarity in gene profiles among different tissues of same function. We note that most of the digestion-related organs developed from endoderm, which might explain their similarity in gene profiles and functions (Zorn AM *et al., Annu Rev Cell Dev Biol* 2009, 25:221-251). In addition, we suspected that the epithelial cells of same digestive functions might also share similar response to pathogens or stimulus (Gunther *et al., Semin Immunopathol* 2018, 40:555-565).

As suggested, we have removed genes encoding immunoglobulins from the downstream analysis in this revision. We have also updated the above information in the **Results**, **Discussion,** and **Methods**, sections. Please kindly check at the lines 7-9 in the 3^rd^ paragraph, Page 14; the lines 14-17 in the 1^st^ paragraph, Page 15; the lines 10-12 in the 2^nd^ paragraph, Page 16; the last three sentences in the 2^nd^ paragraph, Page 22; the lines 2, 3 in the 1^st^ paragraph, Page 29

Thanks. Please correct above the list of colon samples: duodenum is not colon (marked in dark red above).

**Response**: Thanks for pointing out the typo. Duodenum is a part of the small intestine as described previously. We have corrected the typo in the response letter. We ensure that duodenum has been included correctly as a part of the small intestine in our manuscript.

**Response Figure 21**. Comparisons of gene profiles and functions of cells between digestive and non-digestive organs.

**A, B**. Phylogenetic tree of tissues in AHCA (**A**) or Han *et al.*’s HCL (**B**) dataset, based on their normalized mean expression values of epithelial cells (correlation distance metric, complete linkage). Only genes with ln(fold-change) above 0.25, *p.adjust* < 0.05 and pct.1 ≥ 0.2 in each organ were included in the analysis. Organs with less than 50 cells were excluded.

**C, D**. GSVA analysis showed the pathways (hallmark and curated metabolic pathways) with significantly different activation between non-digestive and digestive cell clusters in both the AHCA (**C**) and Han *et al.*’s HCL (**D**) dataset. Activation was determined using GSVA and comparison was tested with an R package limma.

**Third round of review:**

**Reviewer 3**

We now support the publication of this work. We thank the authors for addressing the final raised points about IF experiments, nomenclature and conclusions drawn from their observations.

­

**Response Figure 22**. Heatmap of scaled transcription factor activities.

**A, B**. Heatmap of the active scores for epithelial cell subtypes as numbered on top in our AHCA (**A**) and Han *et al.*’s HCL (**B**) datasets. Activation score was based on the expression regulated by transcription factors (TFs), as estimated using SCENIC analysis. Shown are the top 10 TFs having the highest difference in expression regulation estimates between each cluster and all other cells, tested with a Wilcoxon rank-sum test. TFs with similar activity across multiple digestive or non-digestive organ-derived clusters were outlined in green dashed boxes.

**Response Figure 23**. GO analysis results of each epithelial cell cluster.

**A, B**. Gene Ontology enrichment analysis results of each epithelial cell cluster in digestive organs (**A**) and non-digestive organs (**B**). Cell clusters were coloured according to their -log_10_(*P*-values). Only the top 20 significant terms (*p-value* < 0.05) were shown.
